# Spatio-temporal distribution of Crimean-Congo Hemorrhagic Fever and its relationship with climate factors in Pakistan: A decade-long experience from tertiary care laboratory network

**DOI:** 10.1371/journal.pone.0320495

**Published:** 2025-05-12

**Authors:** Muhammad Abbas Abid, Joveria Farooqi, Najia Ghanchi, Rabiya Owais, Ayesha Sadiqa, Humaira Shafaq, Erum Khan

**Affiliations:** Department of Pathology & Laboratory Medicine, Aga Khan University, Karachi, Pakistan; Nigerian Institute of Medical Research, NIGERIA

## Abstract

Pakistan is at high-risk for Crimean-Congo Hemorrhagic Fever (CCHF) outbreaks due to its geographic location and disease burden in bordering countries. We aimed to study the spatio-temporal distribution of human CCHF cases in Pakistan and to observe its correlation with temperature, precipitation and seasonal variation. Retrospective data of test requests generated for CCHF at country-wide patient sample collection points (n = 307) across Pakistan from 2012 to 2023 was extracted for analysis. Average monthly temperature and precipitation data was used in the Poisson regression method to examine the effect on the number of cases. A total of 2,559 patients were clinically suspected of CCHF in 39 cities with 547 confirmed positive for CCHF in 10 cities using real-time PCR assay, with a positivity rate of 21.37% and a male predominance (84.6%). Most of the confirmed cases (57.6%, n = 315) were detected between 2016 and 2019 while 97.4% (n = 533) detected in 3 cities. Highest number of cases were reported during summer (p < 0.001) with 41.13% of confirmed cases reported in the months of August and September. A positive correlation of suspected cases with temperature was observed in Karachi and Quetta with a lag of zero months (p = 0.000), and a negative correlation was observed with precipitation for Karachi and Peshawar with a lag of 2 months (p = 0.000). Case fatality rate for CCHF patients admitted at Aga Khan University Hospital was 45.8%. CCHF is on the rise in Pakistan. Positive cases are concentrated within 3 cities where human and animal migration rates are high. Outbreak situations occur when multiple factors coincide. Seasonal and climatic patterns can be used as predictors of disease by policymakers for strict implementation on animal regulation, transport, and surveillance of animal migration to curtail outbreak situations in Pakistan.

## Introduction

Vector-borne diseases (VBD) pose an emerging global health threat. An estimated 80% global population is at risk of contracting at least one VBD in their lifetime and is responsible for more than 700,000 deaths annually [1,2]. The prevalence of VBD has continuously increased over the recent decades [3]. Factors such as climate change, rapid urbanization, and socio-economic inequalities have been the primary factors driving this increase. Other risk factors include natural disasters, humanitarian crisis, armed conflicts and mass human and animal migration [4].

Crimean-Congo Hemorrhagic Fever (CCHF) is a tick-borne zoonotic disease caused by Crimean-Congo Hemorrhagic Orthonairovirus (CCHFV) belonging to family *Nairoviridae*, genus *Orthonairovirus* [5]. CCHF has the most extensive geographic distribution of the medically important tick-borne viral diseases, and a case fatality rate reported as high as 80% in humans [6]. Wild animals are known to play a significant role in sylvatic CCHFV transmission to humans including hunters, wildlife workers and wet market workers [7]. Virus amplification and transmission to humans is promoted by domestic animals and *Hyalomma* ticks [8]. CCHFV has an enzootic cycle between ticks and mammals. The geographical distribution of CCHFV mimics that of the *Hyalomma* tick species [9].

Weather and environmental factors, including seasonal variation and temperature, affect vector distribution and hence, affect the transmission of disease [9]. *Hyalomma* ticks spend most of their life cycle in natural environments outside hosts, and tick distribution is greatly affected by ecological factors, such as climatic conditions [10]. Temperature and precipitation are the primary factors affecting tick distribution [11]. For example, *Hyalomma marginatum* requires a temperature sum as high as 3000–4000 °C in 1 year to complete its life cycle. Similarly, *Hyalomma marginatum* larva nymph molting occurs faster at higher temperatures (28 °C) as compared to lower temperatures (18 °C) [12,13]. Being a tropical country, the environmental and vegetational characteristics of Pakistan provide the right conditions for the propagation of the *Hyalomma* tick species.

Data related to tick fauna and its distribution in Pakistan is quite limited. However, studies from the Punjab region of Pakistan report a rich tick fauna, with the highest prevalence that of *Hyalomma* (15%) followed by *Boophilus* (12%), *Haemaphysalis* (5%) and *Rhipicephalus* (3%). These ticks propagate predominantly on small mammals and birds. Most of the rural population in Pakistan rely on livestock, particularly small ruminants, for dairy and meat products [14]. There are estimated 78.2 million goats and 31.2 million sheep raised in Pakistan for human consumption. Hence, there is constant human-animal interaction throughout Pakistan [15].

Most of the Pakistan has arid region with dry temperatures, that *Hyalomma* ticks prefer for its propagation [16]. With rising global temperatures, the duration of hot and dry weather has also increased in Pakistan, risking excessive propagation of tick vector that carry the CCHFV [17]. In addition, Pakistan is increasingly witnessing effects of the El Nino climate phenomenon, that contributes to increased temperature and reduced cloud covers disrupting normal monsoon rainfall patterns. These El Nino changes lead to either intense drought-like conditions, or excessive rainfall leading to flash floods, as were witnessed in Sindh region in 2022 [18,19]. These weather patterns disrupt the normal habitat, often bringing humans and animals in close concentration and hence increasing risk of exposure. Alteration in landscape and changes in agricultural land due to climate change may also influence survival and reproduction of ticks that may trigger outbreak situations in endemic regions in Pakistan.

Pakistan is categorized as a country with 5–49 cases of CCHF per year [20]. However, since it is bordered by Iran and Afghanistan on the west, categorized by WHO as countries with >50 cases per year, Pakistan remains at a high risk for CCHF outbreaks [21]. Additionally Pakistan has a long porous border with Afghanistan, making Pakistan a host to one of the highest number of refugees in the world with constant influx of humans and potentially infected animals, creating a high risk of CCHF outbreak [22]. Cases are often related to occupational exposure in farmers, abattoir workers, veterinarians, and healthcare workers [5].

There is little data about spatial and temporal distribution of CCHF in Pakistan. Although, CCHF is one of the reportable diseases in Pakistan, however due to limited clinical and diagnostic capacity to detect positive patients, most of the cases come to government attention during an outbreak situation. Further, the correlation of seasonal and climatic factors with CCHF cases has not been thoroughly studied in Pakistan. We aimed to study the spatio-temporal distribution of human CCHF cases, using geographic correlates of over 300 laboratory sample collection points (CPs) spread over 100 cities across Pakistan from 2012 to 2023. We also aimed to analyze its correlation with seasonal change and climatic factors using meteorological data of temperature and precipitation during that period.

## Methods

### Study site

**Fig 1:**
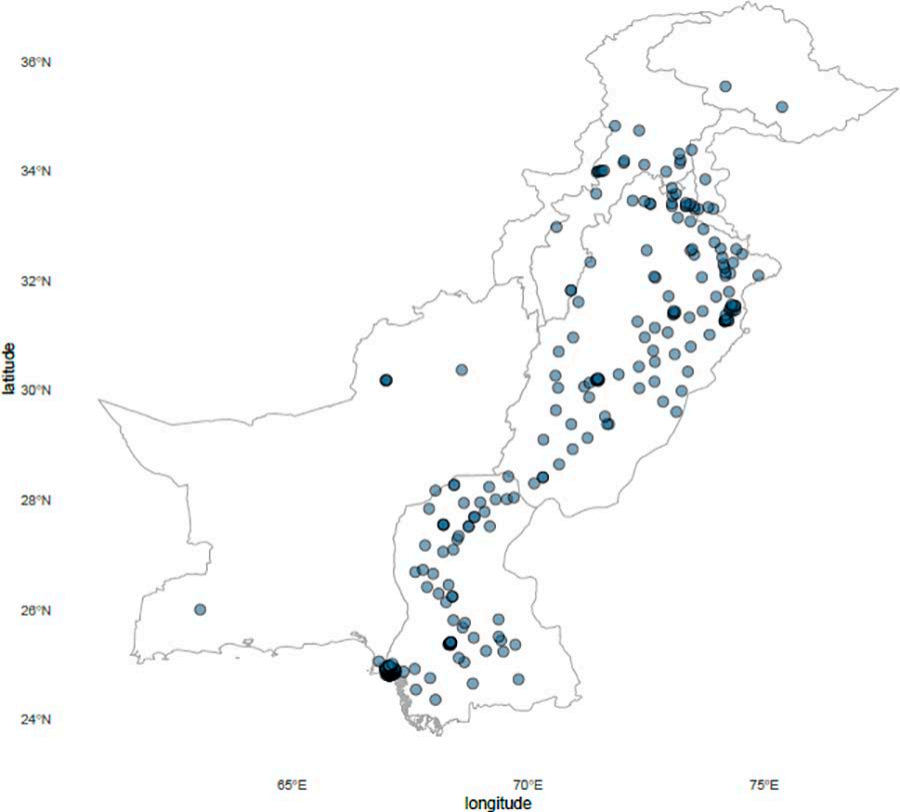
Distribution of Aga Khan University Clinical Laboratories collection points across Pakistan. Geographic distribution of 307 collection points of Aga Khan University Clinical Laboratories across Pakistan. The collection points are in all 5 provinces of the country, in and around all major cities to have a maximum catchment area. (Map made in R; package ‘sf’. Base map: GADM. Data Source: Aga Khan University Hospital Clinical Laboratories).

The study was carried out in the Section of Microbiology, Department of Pathology and Laboratory Medicine, The Aga Khan University (AKU), Pakistan. The Aga Khan University Hospital (AKUH) Clinical Laboratory is College of American Pathologists (CAP) accredited, largest diagnostic laboratory systems in Pakistan. It works on Hub-Spoke model with one central lab located in Karachi, south end of the country. It has an extensive network consisting of more than 300 sample collection points (CPs) spread across 100 major cities and towns in all 5 provinces of Pakistan (Fig 1). Sample collected from each CP correlates with the geographical address of the patient’s residence/ health care facility and is the site of disease acquisition in most cases. Samples collected from the outreach CPs are transported to the main lab using strict protocols that include all biosafety considerations.

### Data collection

Retrospective data of test requests for CCHF generated by the lab from all the CPs across Pakistan from January 1st, 2012 to May 31st, 2023, was extracted for analysis, along with the relevant demographic details. The laboratory testing was performed using reverse transcriptase-PCR (RT-PCR) assay within the Section of Molecular Pathology at AKU. Data was categorized yearly to study the city-wise distribution and demographic variables. All samples received for testing were considered suspected CCHFV cases. Samples that tested positive by RT-PCR were considered confirmed for this study purpose.

Monthly climate data of the cities of interest was extracted from the online climate database of Tokyo Climate Center of the Japan Meteorologic Agency, part of the World Meteorologic Organization (WMO) Regional Climate Center in RA II.

### Ethical statement

The study received exemption from the AKU Ethics Review Committee (ERC): (ERC # 2024-10067-29384)

### Analysis

Data was extracted, aggregated, cleaned, and compiled using Python and visualized using Microsoft Power BI and R. Statistical analysis was conducted using STATA (STATA 17, StataCorp, College Station, TX). Kruskal Wallis H Test was used to check the distribution of data and Chi-Square was used to determine differences in various groups. Poisson regression was applied to observe the effect of climatic factors on positive cases in the cities with the highest number of cases with a lag of up to 6 months. A p-value of < 0.05 was considered statistically significant. Spatial distribution was recorded using the geo-location of the sample collection unit where the patient test request was generated with suspected CCHFV infection.

## Results

### Demographic and geographic distribution of suspected and confirmed CCHF cases

**Fig 2:**
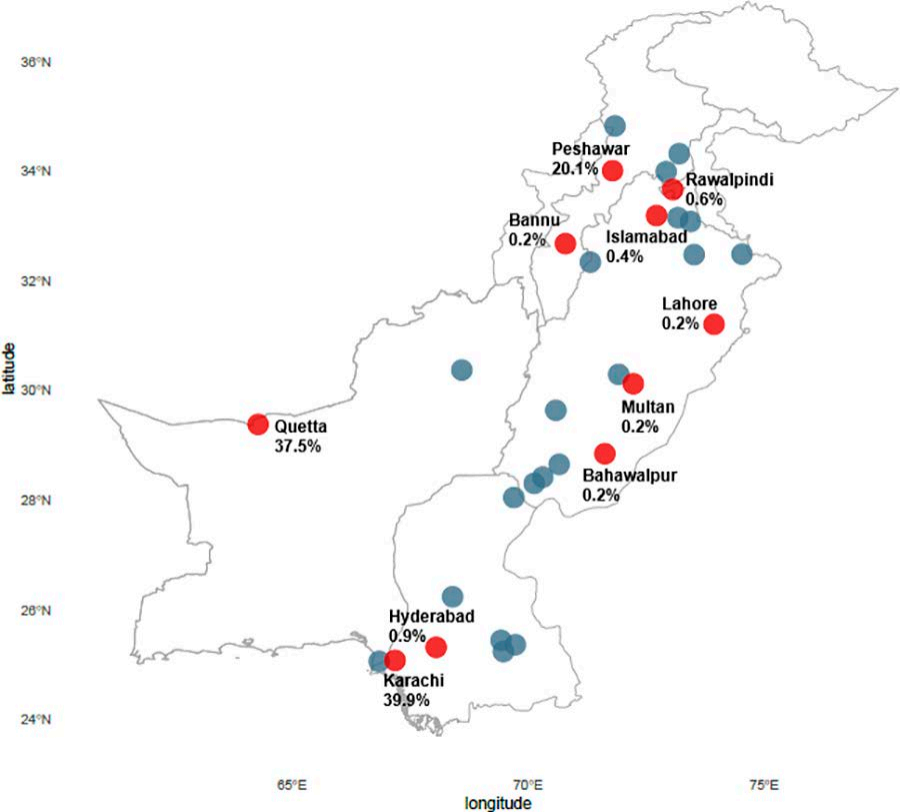
Distribution of suspected and confirmed CCHF cases across Pakistan. Geographic distribution of suspected and laboratory confirmed CCHF cases across Pakistan (2012 -2023). Samples of suspected cases were received from 39 cities of Pakistan. The blue circles represent the cities from where only suspected cases were received with no confirmed case. The red circles represent the 10 cities from where confirmed cases were reported. The cities from where the confirmed cases were reported are labelled, along with the percentage share of the total number of confirmed cases. (Map made in R; package ‘sf’. Base map: GADM. Data Source: Aga Khan University Hospital Clinical Laboratories).

**Fig 3:**
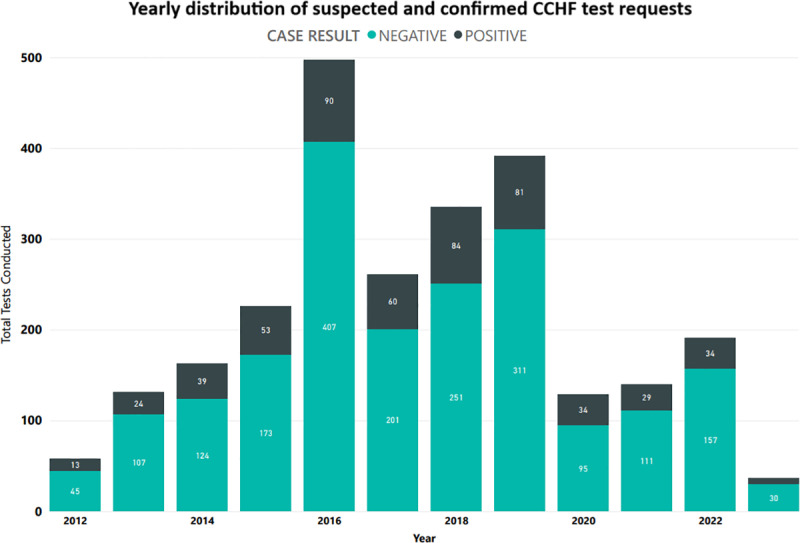
Temporal distribution of CCHF cases in Pakistan. Temporal distribution of suspected and confirmed CCHF cases based on Aga Khan University clinical lab tests (2012-2023). The bars represent the total number of cases received each year. The bars shaded light green represent negative test results and bars shaded dark green represent confirmed positive test results.

A total of 2,559 test requests were generated for suspected CCHF from 39 different cities spread across 5 provinces of the country (Fig 2). Of the total test requests generated, 76.7% (n = 1,962) were for male patients and the median age was 30 years (IQR, 21–42). The age range for suspected cases was 1 month to 90 years. The highest number of suspected cases (91.5%, n = 2,342) were reported from the following 3 cities: Karachi (54.4%, n = 1,393), Quetta (21.38%, n = 547) and Peshawar (15.7%, n = 402). The highest number of suspected cases were seen in the year 2016 (n = 497), followed by 2019 (n = 392), 2018 (n = 335), and 2017 (n = 261). Fig 3 displayed the year-wise distribution of suspected cases (S1 File). When comparing data for 3 cities with the highest number of suspected cases, 56.5% (n = 1,323) test requests were for age 18–38 years, 23.0% (n = 538) for 39–59 years, 13.0% (n = 304) for less than 18 years, and 7.6% (n = 177) for above 60 years, with significant difference between the age groups (p = 0.02).

Of the total suspected cases, 547 were confirmed for CCHF virus, with a positivity rate of 21.37%. Of the total confirmed cases, 84.6% (n = 463) were male. The median age was 32 years (IQR, 25–45). Confirmed cases were reported in 10 different cities, with 97.4% (n = 533) in the following 3 cities: 39.9% (n = 218) in Karachi, 37.5% (n = 205) in Quetta, and 20.1% (n = 110) in Peshawar. Fig 3 displays the temporal distribution of confirmed cases throughout the country. The highest number of confirmed cases were seen in 2016 (n = 90), followed by 2018 (n = 84), 2019 (n = 81), and 2017 (n = 60) (Fig 3).

**Fig 4:**
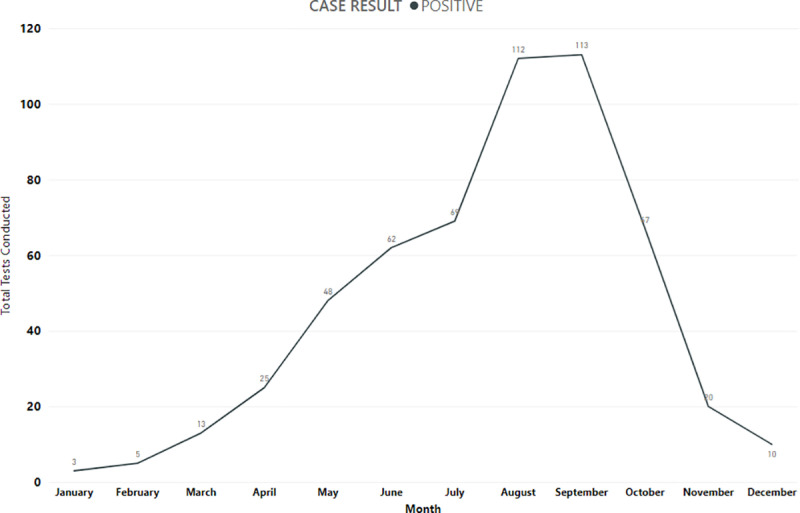
Month-wise distribution of confirmed CCHF cases in Pakistan. Month-wise distribution of confirmed CCHF cases across Pakistan (2012–2023). The highest number of confirmed CCHF cases were detected during August and September while the least number of positive cases were detected during January and February.

The highest number of confirmed cases were detected in the month of September (n = 113) and August (n = 112), whereas the least number of cases were detected in January (n = 3) over the entire study period. Fig 4 shows the number of positive cases detected month-wise.

The highest number of suspected cases were reported in autumn (n = 1058), and the highest number of confirmed cases were reported in summer (n = 243). Seasonal variation in suspected and confirmed cases is detailed in Table 1.

**Table 1:** Seasonal distribution of suspected and confirmed CCHF cases (2012–2023) in Pakistan. The highest number of cases were suspected in autumn, whereas the highest number of confirmed cases were detected in summer. Winters recorded the lowest number of suspected and confirmed cases.

	Total	Spring	Summer	Autum	Winter	p-value
Result	N = 2,559	N = 422	N = 861	N = 1,058	N = 218	
Negative	2,012 (78.6%)	336 (79.6%)	618 (71.8%)	858 (81.1%)	200 (91.7%)	<0.001
Positive	547 (21.4%)	86 (20.4%)	243 (28.2%)	200 (18.9%)	18 (8.3%)	

The highest number of confirmed cases within the 3 cities with the highest case burden (Karachi, Quetta, Peshawar) was seen in summer (28.2%, n = 243), followed by spring (20.4%, n = 86), autumn (18.9%, n = 200), and winter (8.3%, n = 18) (p=<0.001).

### Effect of climatic factors (temperature and precipitation) on suspected and confirmed CCHF cases

In the Poisson regression analysis, a positive correlation for temperature with the number of suspected cases was observed with the highest correlation for Karachi and Quetta for a lag of zero months (Temperature 0; p = 0.000). The correlation with temperature 0 in Peshawar was not statistically significant. A negative correlation was observed with precipitation with the highest significant correlation was for Precipitation 4 for Karachi and Peshawar (p = 0.000) and precipitation 1 for Quetta (p = 0.001). The significant observations in the Poisson regression analysis for the 3 cities are shown in Table 2.

**Table 2:** Results of the Poisson regression analysis of temperature and precipitation with the number of suspected cases for the 3 cities with the highest number of cases (Karachi, Quetta, Peshawar).

	Coefficient	Exp (Coefficient)	Std. err.	z	P>|z|	[95% conf. interval]	
**Karachi**							
Temperature 0	.119446	1.126872	.0147859	9.10	0.000	1.098262	1.156228
Precipitation 0	.0023893	1.002392	.0005562	4.31	0.000	1.001303	1.003483
Temperature 2	.093493	1.098003	.0137692	7.46	0.000	1.071345	1.125324
Precipitation 2	-.0025457	.9974575	.0005505	-4.61	0.000	.9963792	.9985371
Temperature 4	.0926112	1.097035	.0109739	9.26	0.000	1.075736	1.118756
Precipitation 4	-.0031889	.9968161	.0009087	-3.50	0.000	.9950368	.9985987
Precipitation 5	-.0030994	.9969054	.0010963	-2.82	0.005	.9947589	.9990565
Temperature 6	.0576958	1.059393	.0115581	5.29	0.000	1.03698	1.08229
**Quetta**							
Temperature 0	.0788176	1.082007	.0102334	8.33	0.000	1.062135	1.102252
Precipitation 0	-.0075581	.9924703	.0024107	-3.11	0.002	.9877567	.9972064
Precipitation 1	-.0071524	.9928731	.0020832	-3.41	0.001	.9887984	.9969645
Temperature 3	.0318987	1.032413	.0072705	4.53	0.000	1.018261	1.046762
Temperature 5	.0162709	1.016404	.0080536	2.05	0.040	1.000741	1.032312
Temperature 6	.0055326	1.005548	.0012576	4.42	0.000	1.003086	1.008016
**Peshawar**							
Temperature 2	.0529502	1.054377	.0104676	5.33	0.000	1.034059	1.075094
Temperature 5	-.0309687	.9695059	.0081219	-3.70	0.000	.9537173	.985556
Temperature 6	.0151847	1.015301	.0047118	3.27	0.001	1.006107	1.024578
Precipitation 2	.006536	1.006557	.0016677	3.94	0.000	1.003294	1.009831
Precipitation 4	-.0079138	.9921175	.0019408	-4.05	0.000	.9883208	.9959287

**Fig 5:**
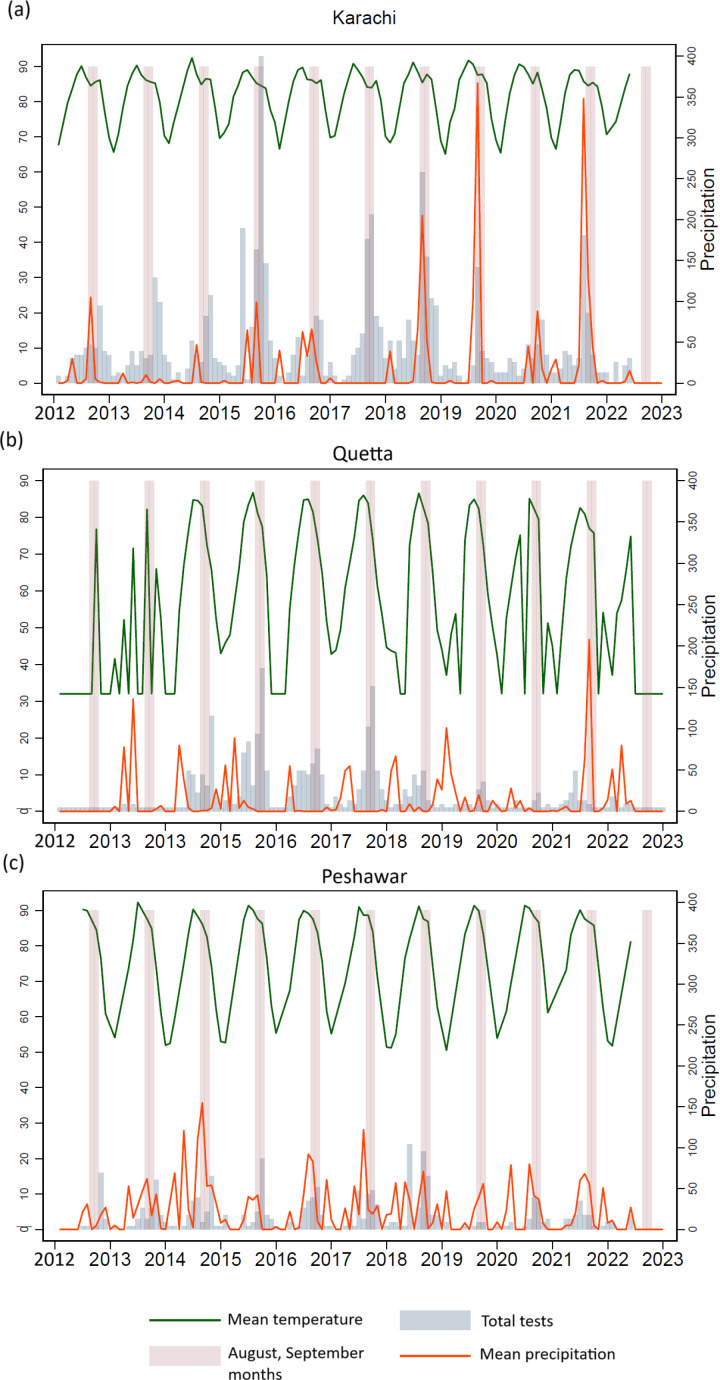
Effect of temperature, precipitation and seasonality on the number of suspected CCHF cases in 3 cities of Pakistan. Linear distribution of suspected and confirmed CCHF cases from 2012 to 2023 in (a) Karachi, (b) Quetta and (c) Peshawar. The red line represents the monthly mean precipitation (mm) whereas, the green line represents the monthly mean temperature (°F). The pink bars highlight the months of August and September.

When plotting the total number of suspected and confirmed cases each month across timeline for 3 cities with the highest CCHF burden (Karachi, Quetta, Peshawar) for the entire study period, a noticble finding was clustering of these cases for the months of August and September as shown in Fig 5.

### Survival of CCHF in-patients at AKUH

In-patient data was available for 107 patients who were admitted to AKUH, Karachi. Out of these, 11 patients left against medical advice hence, their survival data was unavailable. For the remaining 96 cases, 44 patients died in hospital, while 52 were discharged after achieving clinical stability, making a case-fatality rate of 45.8%. Survival data for patients not admitted to AKUH was not available.

## Discussion

There is limited knowledge about the actual burden and distribution of CCHF in Pakistan [23]. This study was an attempt to assess the geographical distribution and seasonal trend of CCHF using data bank of the largest clinical laboratory in Pakistan. We report a total of 547 confirmed positive cases of CCHF over an eleven and a half-year period.

The highest number of cases were reported from 2015–2019, with no year reporting zero case. In 2016, Pakistan witnessed the largest number of positive CCHF cases (n = 90) as a possible outbreak situation. Our findings are substantiated by other similar reports from Pakistan; Haider et al. reported an outbreak in 2016 with 22 confirmed cases at their facility in the city of Quetta [24]. Similarly, Karim et al. reported 86 confirmed cases of CCHF in the same year from all provinces of Pakistan [25]. Most of these outbreaks could be associated with cattle-herding and livestock movement, however due to lack of resources, these factors have not been scientifically studied hence, these risks remain unidentified. Detailed studies at the host-pathogen-vector-environment interface are therefore required to delineate the underlying mechanisms and risk factors to prevent such outbreaks.

We found three cities Karachi, Quetta, and Peshawar to be the epicenters for CCHF infections. These cities accounted for 91.5% of total suspected cases and 97.4% of total confirmed cases. The cities of Quetta and Peshawar are along the Pakistan-Iran-Afghanistan border with both cities hosting a high number of refugees, especially from Afghanistan. The long porous border with Afghanistan makes Pakistan a host to one of the highest number of refugees in the world with a constant influx of humans and potentially infected animals, creating a high risk of CCHF outbreaks [22]. In addition, routing of cattle from Iran and Afghanistan to the rest of the country, specially Karachi is a constant feature [26].

The city of Karachi, where CCHF cases were the highest, is the largest and the most densely populated city of Pakistan, and the 12^th^ largest city in the world with a population of over 17.6 million [27]. It is host to the highest number of refugees and immigrants in Pakistan, including both domestic and international [28]. Another potential factor is a constant animal herd movement, with almost half a million cows, goats and camels sacrificed during the annual festival of Eid ul Adha [29]. These animals are transported to Karachi from all over the country and are handled and sold in various cattle markets within and outside the city limits. Poor implementation of regulation of livestock movement and inadequate animal health-checks make tracking and surveillance of infected animals’ inter-city movement difficult. Strong policy implementation efforts are desperately required. In fact, our data supports this view as we noticed a major reduction in positive cases in 2020, possibly due to COVID-19 lockdown and movement restrictions. This indicates the effectiveness of policy implementation based on the number of cases. After 2020, the number of cases shows a rising trend that will likely continue to increase unless preventive measures are implemented by the governing agencies and concrete steps are taken to control spread.

Transmission and distribution of CCHF follows the distribution of the *Hyalomma* tick species. Environmental factors, such as climate and geographic features affect tick population, and hence influence the disease spread [30]. Temperature and precipitation are the primary climatic factors that affect tick distribution. In our study, we report a statistically significant positive correlation between suspected cases and temperature with a zero-month lag for Karachi and Quetta. *Hyalomma marginatum* requires a temperature sum of 3000–4000 °C in 1 year to complete its lifecycle, with larva nymph molting occurring faster at higher temperatures (28 °C) as compared to lower temperatures (18 °C) [12,13]. Choubdar et al. reported that tick abundance was 1.4 times higher in hot season as compared to cold season in Iran. They also reported that tick abundance was higher for *H. dromedarii* and *H. anatolicum* species in years with high mean annual temperatures [16]. An increase in CCHF cases during higher temperatures could be attributed to several factors. Greater tick activity is observed under higher temperatures as adult *Hyalomma spp*. are highly motile in seeking out mammalian hosts in higher temperatures (questing). Higher temperatures result in more hours spent outdoors by humans (longer daylight hours). Increased agriculture and livestock grazing provides increased chances for human-tick contact [31]. Combining these factors leads to increased vector population as well as increased vector-to-host transmission during higher temperatures.

Our findings highlighted a significant negative correlation between suspected cases and precipitation with a 4-month lag for Karachi and Peshawar. Similar findings have been reported by Choubdar et al. who reported negative association between annual mean precipitation and tick abundance or tick species community in Iran. They reported that a reduction in precipitation favored *Hyalomma tick* abundance, especially *H. dromedarii* and *H. anatolicum* [16]. Higher rainfall provides unfavorable conditions for *Hyalomma spp*. tick development and host seeking activity [32]. A reverse impact of precipitation on CCHF incidence was also reported in Iran and Senegal [33,34].

We also observed significant seasonal variation in suspected as well as confirmed CCHF cases. A significantly higher number of suspected and confirmed cases were observed in summer and autumn, while the least number of suspected and confirmed cases were observed in winter (p < 0.001). Abbas et al. also reported high seasonal variation in CCHF cases and reported average monthly ambient temperature as a significant predictor of CCHF hospitalizations [35]. But their study was limited to the province of Baluchistan and was limited by the number of positive cases in the 3-year period for which it was performed. Ansari et al. carried out a similar study in Iran and reported that mean temperature, accumulated rainfall, and maximum relative humidity were significantly correlated with the monthly incidence of CCHF [33]. These climatic and environmental risk factors need to be studied in detail under local context, hence research studies cutting across human, animal and vector interface are required to understand this complex interaction and identify evidence-based preventive strategies for spread of CCHF in Pakistan.

We found the CCHF case-fatality rate for patients admitted to AKUH to be 45.8%. WHO reports a global CCHF case-fatality ratio of 10–40% [21]. The cause of high fatality observed in our study can be attributed to late diagnosis, limited diagnostic facilities, and a dearth of medical facilities that can handle CCHF patients in primary and secondary care hospitals, due to which patients have to travel long distances to a major tertiary-care facility that can effectively treat and isolate CCHF patients. This results in delayed presentation of patients with bleeding diathesis resulting in outbreaks among health care professionals due to poor hospital infection control policies. As of November 3^rd^, 2023, 112 cases had been reported in Pakistan in the year (NIH-Pakistan), majority from the province of Baluchistan [36]. Many of these cases were reported in healthcare workers who were involved in the treatment of CCHF patients admitted to a local hospital in Quetta [37].

Limitations of this study include being a single center study, however, since the clinical laboratory at AKUH has largest network of sample collection units that has footprint across the country and has representation in all major cities of Pakistan, our laboratory data can be considered as national representative. Another limitation is that patients’ exact home addresses could not be traced, however, the geographic corelates were town-specific and represent the area where the patient appeared for sampling. The climate data used was reported as monthly means. More rigorous climate data can help perform better correlation and forecasting studies to understand the effect of climatic factors on the number of cases. To our knowledge, this is the single largest study for the distribution of CCHF confirmed cases in Pakistan, with data from all 5 provinces for a period of over 11 years.

## Conclusion

CCHF has been on the rise in Pakistan. Most of the cases are reported from within 3 major cities of Pakistan where human and animal migration are high. CCHF cases were positively associated with summer season and increased temperature, while negatively associated with precipitation. Acute outbreaks occur when multiple factors overlap. CCHF prevalence in livestock and screening of ticks and nymphs for the presence of virus would provide better estimation of disease burden and identifying high-risk areas. These climatic and environmental risk factors need to be studied in detail under local context, hence research studies cutting across human, animal and vector interface are required to understand this complex interaction and identify evidence-based preventive strategies for spread of CCHF in Pakistan.

## Supporting information

S1 File Crimean-Congo Hemorrhagic Fever Virus test requests received from 2012 to 2023 at the Aga Khan University, Hospital Clinical Laboratories, Pakistan. (PDF)

S2 File Monthly mean temperature and precipitation for the cities of Karachi, Quetta and Peshawar extracted from the online climate database of Tokyo Climate Center of the Japan Meteorologic Agency, part of the World Meteorologic Organization (WMO) Regional Climate Center in RA II. https://ds.data.jma.go.jp/tcc/tcc/products/climate/climatview/list.php?r=0&y=2025&m=1&s=1&e=0&k=0
(PDF)
