# Correction: Spatio-temporal distribution of Crimean-Congo Hemorrhagic Fever and its relationship with climate factors in Pakistan: A decade-long experience from tertiary care laboratory network

**DOI:** 10.1371/journal.pone.0341256

**Published:** 2026-01-20

**Authors:** Muhammad Abbas Abid, Joveria Farooqi, Najia Ghanchi, Rabiya Owais, Ayesha Sadiqa, Humaira Shafaq, Erum Khan

The Acknowledgment statement for this article is incorrect. The correct Acknowledgement statement is as follows: Research reported in this publication was supported by the Fogarty International Center of the National Institutes of Health (D43TW012733−02 to EK). The content is solely the responsibility of the authors and does not necessarily represent the official views of the National Institutes of Health.
