# Supplementary material for: Spatio-temporal distribution of Crimean-Congo Hemorrhagic Fever and its relationship with climate factors in Pakistan: A decade-long experience from tertiary care laboratory network
Source: PLoS One. 2025 May 12;20(5):e0320495. doi: 10.1371/journal.pone.0320495 (PMC12068632; doi:10.1371/journal.pone.0320495)
Supplement: S1 File — (PDF) [file pone.0320495.s001.pdf]

Supplementary File 1

Crimean-Congo Hemorrhagic Fever Virus test requests received from 2012 to 2023 at the Aga Khan University Hospital Clinical Laboratories, Pakistan.

S. No	Year	Ordered Test	Province	City	Test Result
0	2016	CCHF BY PCR	SINDH	Karachi	NEGATIVE
1	2018	CCHF BY PCR	SINDH	Karachi	NEGATIVE
2	2016	CCHF BY PCR	SINDH	Karachi	NEGATIVE
3	2015	CCHF BY PCR	SINDH	Karachi	NEGATIVE
4	2016	CCHF BY PCR	SINDH	Karachi	NEGATIVE
5	2014	CCHF BY PCR	SINDH	Karachi	NEGATIVE
6	2015	CCHF BY PCR	SINDH	Karachi	NEGATIVE
7	2016	CCHF BY PCR	SINDH	Karachi	NEGATIVE
8	2019	CCHF BY PCR	SINDH	Karachi	NEGATIVE
9	2014	CCHF BY PCR	SINDH	Karachi	NEGATIVE
10	2016	CCHF BY PCR	SINDH	Karachi	NEGATIVE
11	2019	CCHF BY PCR	SINDH	Karachi	NEGATIVE
12	2013	CCHF BY PCR	SINDH	Karachi	NEGATIVE
13	2022	CCHF BY PCR	SINDH	Karachi	NEGATIVE
14	2019	CCHF BY PCR	SINDH	Karachi	NEGATIVE
15	2017	CCHF BY PCR	SINDH	Karachi	NEGATIVE
16	2016	CCHF BY PCR	SINDH	Karachi	NEGATIVE
17	2016	CCHF BY PCR	SINDH	Karachi	NEGATIVE
18	2019	CCHF BY PCR	SINDH	Karachi	NEGATIVE
19	2018	CCHF BY PCR	SINDH	Karachi	NEGATIVE
20	2016	CCHF BY PCR	SINDH	Karachi	NEGATIVE
21	2015	CCHF BY PCR	SINDH	Karachi	NEGATIVE
22	2016	CCHF BY PCR	SINDH	Karachi	NEGATIVE
23	2016	CCHF BY PCR	SINDH	Karachi	NEGATIVE
24	2013	CCHF BY PCR	SINDH	Karachi	NEGATIVE
25	2014	CCHF BY PCR	SINDH	Karachi	NEGATIVE
26	2016	CCHF BY PCR	SINDH	Karachi	NEGATIVE
27	2013	CCHF BY PCR	SINDH	Karachi	NEGATIVE
28	2015	CCHF BY PCR	SINDH	Karachi	NEGATIVE
29	2016	CCHF BY PCR	SINDH	Karachi	NEGATIVE
30	2016	CCHF BY PCR	SINDH	Karachi	NEGATIVE
31	2016	CCHF BY PCR	SINDH	Karachi	NEGATIVE
32	2012	CCHF BY PCR	SINDH	Karachi	NEGATIVE
33	2015	CCHF BY PCR	SINDH	Karachi	NEGATIVE
34	2016	CCHF BY PCR	SINDH	Karachi	NEGATIVE
35	2016	CCHF BY PCR	SINDH	Karachi	NEGATIVE
36	2022	CCHF BY PCR	SINDH	Karachi	NEGATIVE
37	2016	CCHF BY PCR	SINDH	Karachi	NEGATIVE

38	2017	CCHF BY PCR	SINDH	Karachi	NEGATIVE
39	2016	CCHF BY PCR	SINDH	Karachi	NEGATIVE
40	2018	CCHF BY PCR	SINDH	Karachi	NEGATIVE
41	2016	CCHF BY PCR	SINDH	Karachi	NEGATIVE
42	2016	CCHF BY PCR	SINDH	Karachi	POSITIVE
43	2016	CCHF BY PCR	SINDH	Karachi	NEGATIVE
44	2018	CCHF BY PCR	SINDH	Karachi	NEGATIVE
45	2018	CCHF BY PCR	SINDH	Karachi	NEGATIVE
46	2017	CCHF BY PCR	SINDH	Karachi	NEGATIVE
47	2015	CCHF BY PCR	SINDH	Karachi	NEGATIVE
48	2014	CCHF BY PCR	SINDH	Karachi	POSITIVE
49	2017	CCHF BY PCR	SINDH	Karachi	NEGATIVE
50	2016	CCHF BY PCR	SINDH	Karachi	NEGATIVE
51	2022	CCHF BY PCR	SINDH	Karachi	NEGATIVE
52	2015	CCHF BY PCR	SINDH	Karachi	NEGATIVE
53	2016	CCHF BY PCR	SINDH	Karachi	NEGATIVE
54	2019	CCHF BY PCR	SINDH	Karachi	NEGATIVE
55	2016	CCHF BY PCR	SINDH	Karachi	NEGATIVE
56	2015	CCHF BY PCR	SINDH	Karachi	NEGATIVE
57	2012	CCHF BY PCR	SINDH	Karachi	NEGATIVE
58	2012	CCHF BY PCR	SINDH	Karachi	POSITIVE
59	2013	CCHF BY PCR	SINDH	Karachi	NEGATIVE
60	2012	CCHF BY PCR	SINDH	Karachi	NEGATIVE
61	2012	CCHF BY PCR	SINDH	Karachi	POSITIVE
62	2012	CCHF BY PCR	SINDH	Karachi	NEGATIVE
63	2012	CCHF BY PCR	SINDH	Karachi	NEGATIVE
64	2012	CCHF BY PCR	SINDH	Karachi	NEGATIVE
65	2012	CCHF BY PCR	SINDH	Karachi	NEGATIVE
66	2012	CCHF BY PCR	SINDH	Karachi	NEGATIVE
67	2012	CCHF BY PCR	SINDH	Karachi	NEGATIVE
68	2012	CCHF BY PCR	SINDH	Karachi	POSITIVE
69	2012	CCHF BY PCR	SINDH	Karachi	NEGATIVE
70	2012	CCHF BY PCR	SINDH	Karachi	POSITIVE
71	2012	CCHF BY PCR	SINDH	Karachi	NEGATIVE
72	2012	CCHF BY PCR	SINDH	Karachi	POSITIVE
73	2012	CCHF BY PCR	SINDH	Karachi	NEGATIVE
74	2017	CCHF BY PCR	SINDH	Karachi	NEGATIVE
75	2012	CCHF BY PCR	SINDH	Karachi	NEGATIVE
76	2012	CCHF BY PCR	SINDH	Karachi	NEGATIVE
77	2012	CCHF BY PCR	SINDH	Karachi	NEGATIVE
78	2012	CCHF BY PCR	SINDH	Karachi	NEGATIVE
79	2012	CCHF BY PCR	SINDH	Karachi	NEGATIVE
80	2012	CCHF BY PCR	SINDH	Karachi	NEGATIVE
81	2012	CCHF BY PCR	SINDH	Karachi	NEGATIVE
82	2013	CCHF BY PCR	SINDH	Karachi	NEGATIVE
83	2013	CCHF BY PCR	SINDH	Karachi	NEGATIVE

84	2013	CCHF BY PCR	SINDH	Karachi	POSITIVE
85	2013	CCHF BY PCR	SINDH	Karachi	NEGATIVE
86	2013	CCHF BY PCR	SINDH	Karachi	NEGATIVE
87	2013	CCHF BY PCR	SINDH	Karachi	NEGATIVE
88	2013	CCHF BY PCR	SINDH	Karachi	NEGATIVE
89	2013	CCHF BY PCR	SINDH	Karachi	NEGATIVE
90	2013	CCHF BY PCR	SINDH	Karachi	NEGATIVE
91	2013	CCHF BY PCR	SINDH	Karachi	POSITIVE
92	2013	CCHF BY PCR	SINDH	Karachi	NEGATIVE
93	2013	CCHF BY PCR	SINDH	Karachi	NEGATIVE
94	2013	CCHF BY PCR	SINDH	Karachi	NEGATIVE
95	2013	CCHF BY PCR	SINDH	Karachi	NEGATIVE
96	2013	CCHF BY PCR	SINDH	Karachi	NEGATIVE
97	2013	CCHF BY PCR	SINDH	Karachi	NEGATIVE
98	2013	CCHF BY PCR	SINDH	Karachi	NEGATIVE
99	2013	CCHF BY PCR	SINDH	Karachi	NEGATIVE
100	2013	CCHF BY PCR	SINDH	Karachi	NEGATIVE
101	2013	CCHF BY PCR	SINDH	Karachi	NEGATIVE
102	2013	CCHF BY PCR	SINDH	Karachi	NEGATIVE
103	2013	CCHF BY PCR	SINDH	Karachi	NEGATIVE
104	2013	CCHF BY PCR	SINDH	Karachi	NEGATIVE
105	2013	CCHF BY PCR	SINDH	Karachi	NEGATIVE
106	2013	CCHF BY PCR	SINDH	Karachi	NEGATIVE
107	2013	CCHF BY PCR	SINDH	Karachi	NEGATIVE
108	2013	CCHF BY PCR	SINDH	Karachi	NEGATIVE
109	2013	CCHF BY PCR	SINDH	Karachi	NEGATIVE
110	2013	CCHF BY PCR	SINDH	Karachi	NEGATIVE
111	2013	CCHF BY PCR	SINDH	Karachi	NEGATIVE
112	2013	CCHF BY PCR	SINDH	Karachi	POSITIVE
113	2013	CCHF BY PCR	SINDH	Karachi	NEGATIVE
114	2013	CCHF BY PCR	SINDH	Karachi	POSITIVE
115	2013	CCHF BY PCR	SINDH	Karachi	NEGATIVE
116	2013	CCHF BY PCR	SINDH	Karachi	NEGATIVE
117	2013	CCHF BY PCR	SINDH	Karachi	NEGATIVE
118	2014	CCHF BY PCR	SINDH	Karachi	POSITIVE
119	2013	CCHF BY PCR	SINDH	Karachi	NEGATIVE
120	2013	CCHF BY PCR	SINDH	Karachi	NEGATIVE
121	2013	CCHF BY PCR	SINDH	Karachi	NEGATIVE
122	2013	CCHF BY PCR	SINDH	Karachi	NEGATIVE
123	2013	CCHF BY PCR	SINDH	Karachi	NEGATIVE
124	2013	CCHF BY PCR	SINDH	Karachi	POSITIVE
125	2013	CCHF BY PCR	SINDH	Karachi	NEGATIVE
126	2014	CCHF BY PCR	SINDH	Karachi	NEGATIVE
127	2014	CCHF BY PCR	SINDH	Karachi	NEGATIVE
128	2014	CCHF BY PCR	SINDH	Karachi	POSITIVE
129	2014	CCHF BY PCR	SINDH	Karachi	NEGATIVE

130	2014	CCHF BY PCR	SINDH	Karachi	NEGATIVE
131	2014	CCHF BY PCR	SINDH	Karachi	NEGATIVE
132	2014	CCHF BY PCR	SINDH	Karachi	NEGATIVE
133	2014	CCHF BY PCR	SINDH	Karachi	POSITIVE
134	2014	CCHF BY PCR	SINDH	Karachi	POSITIVE
135	2014	CCHF BY PCR	SINDH	Karachi	POSITIVE
136	2014	CCHF BY PCR	SINDH	Karachi	NEGATIVE
137	2014	CCHF BY PCR	SINDH	Karachi	NEGATIVE
138	2014	CCHF BY PCR	SINDH	Karachi	NEGATIVE
139	2014	CCHF BY PCR	SINDH	Karachi	NEGATIVE
140	2014	CCHF BY PCR	SINDH	Karachi	NEGATIVE
141	2014	CCHF BY PCR	SINDH	Karachi	NEGATIVE
142	2014	CCHF BY PCR	SINDH	Karachi	NEGATIVE
143	2014	CCHF BY PCR	SINDH	Karachi	NEGATIVE
144	2014	CCHF BY PCR	SINDH	Karachi	NEGATIVE
145	2014	CCHF BY PCR	SINDH	Karachi	NEGATIVE
146	2014	CCHF BY PCR	SINDH	Karachi	NEGATIVE
147	2014	CCHF BY PCR	SINDH	Karachi	NEGATIVE
148	2014	CCHF BY PCR	SINDH	Karachi	POSITIVE
149	2014	CCHF BY PCR	SINDH	Karachi	POSITIVE
150	2014	CCHF BY PCR	SINDH	Karachi	NEGATIVE
151	2014	CCHF BY PCR	SINDH	Karachi	NEGATIVE
152	2014	CCHF BY PCR	SINDH	Karachi	NEGATIVE
153	2017	CCHF BY PCR	SINDH	Karachi	NEGATIVE
154	2014	CCHF BY PCR	SINDH	Karachi	NEGATIVE
155	2014	CCHF BY PCR	SINDH	Karachi	NEGATIVE
156	2014	CCHF BY PCR	SINDH	Karachi	POSITIVE
157	2014	CCHF BY PCR	SINDH	Karachi	NEGATIVE
158	2014	CCHF BY PCR	SINDH	Karachi	NEGATIVE
159	2014	CCHF BY PCR	SINDH	Karachi	NEGATIVE
160	2014	CCHF BY PCR	SINDH	Karachi	NEGATIVE
161	2014	CCHF BY PCR	SINDH	Karachi	POSITIVE
162	2014	CCHF BY PCR	SINDH	Karachi	NEGATIVE
163	2014	CCHF BY PCR	SINDH	Karachi	NEGATIVE
164	2014	CCHF BY PCR	SINDH	Karachi	NEGATIVE
165	2014	CCHF BY PCR	SINDH	Karachi	NEGATIVE
166	2014	CCHF BY PCR	SINDH	Karachi	NEGATIVE
167	2014	CCHF BY PCR	SINDH	Karachi	NEGATIVE
168	2014	CCHF BY PCR	SINDH	Karachi	NEGATIVE
169	2014	CCHF BY PCR	SINDH	Karachi	NEGATIVE
170	2014	CCHF BY PCR	SINDH	Karachi	NEGATIVE
171	2014	CCHF BY PCR	SINDH	Karachi	NEGATIVE
172	2014	CCHF BY PCR	SINDH	Karachi	POSITIVE
173	2014	CCHF BY PCR	SINDH	Karachi	POSITIVE
174	2014	CCHF BY PCR	SINDH	Karachi	NEGATIVE
175	2016	CCHF BY PCR	SINDH	Karachi	NEGATIVE

176	2016	CCHF BY PCR	SINDH	Karachi	NEGATIVE
177	2015	CCHF BY PCR	SINDH	Karachi	NEGATIVE
178	2015	CCHF BY PCR	SINDH	Karachi	NEGATIVE
179	2015	CCHF BY PCR	SINDH	Karachi	NEGATIVE
180	2016	CCHF BY PCR	SINDH	Karachi	NEGATIVE
181	2015	CCHF BY PCR	SINDH	Karachi	NEGATIVE
182	2016	CCHF BY PCR	SINDH	Karachi	NEGATIVE
183	2015	CCHF BY PCR	SINDH	Karachi	NEGATIVE
184	2016	CCHF BY PCR	SINDH	Karachi	NEGATIVE
185	2015	CCHF BY PCR	SINDH	Karachi	NEGATIVE
186	2015	CCHF BY PCR	SINDH	Karachi	NEGATIVE
187	2015	CCHF BY PCR	SINDH	Karachi	NEGATIVE
188	2015	CCHF BY PCR	SINDH	Karachi	NEGATIVE
189	2016	CCHF BY PCR	SINDH	Karachi	NEGATIVE
190	2016	CCHF BY PCR	SINDH	Karachi	NEGATIVE
191	2016	CCHF BY PCR	SINDH	Karachi	NEGATIVE
192	2015	CCHF BY PCR	SINDH	Karachi	POSITIVE
193	2019	CCHF BY PCR	SINDH	Karachi	NEGATIVE
194	2016	CCHF BY PCR	SINDH	Karachi	NEGATIVE
195	2015	CCHF BY PCR	SINDH	Karachi	NEGATIVE
196	2016	CCHF BY PCR	SINDH	Karachi	NEGATIVE
197	2015	CCHF BY PCR	SINDH	Karachi	NEGATIVE
198	2015	CCHF BY PCR	SINDH	Karachi	NEGATIVE
199	2015	CCHF BY PCR	SINDH	Karachi	NEGATIVE
200	2015	CCHF BY PCR	SINDH	Karachi	NEGATIVE
201	2015	CCHF BY PCR	SINDH	Karachi	NEGATIVE
202	2015	CCHF BY PCR	SINDH	Karachi	NEGATIVE
203	2015	CCHF BY PCR	SINDH	Karachi	POSITIVE
204	2015	CCHF BY PCR	SINDH	Karachi	NEGATIVE
205	2015	CCHF BY PCR	SINDH	Karachi	NEGATIVE
206	2015	CCHF BY PCR	SINDH	Karachi	POSITIVE
207	2015	CCHF BY PCR	SINDH	Karachi	NEGATIVE
208	2015	CCHF BY PCR	SINDH	Karachi	NEGATIVE
209	2015	CCHF BY PCR	SINDH	Karachi	NEGATIVE
210	2015	CCHF BY PCR	SINDH	Karachi	NEGATIVE
211	2016	CCHF BY PCR	SINDH	Karachi	NEGATIVE
212	2016	CCHF BY PCR	SINDH	Karachi	NEGATIVE
213	2015	CCHF BY PCR	SINDH	Karachi	POSITIVE
214	2015	CCHF BY PCR	SINDH	Karachi	NEGATIVE
215	2015	CCHF BY PCR	SINDH	Karachi	NEGATIVE
216	2015	CCHF BY PCR	SINDH	Karachi	NEGATIVE
217	2015	CCHF BY PCR	SINDH	Karachi	NEGATIVE
218	2015	CCHF BY PCR	SINDH	Karachi	NEGATIVE
219	2016	CCHF BY PCR	SINDH	Karachi	NEGATIVE
220	2016	CCHF BY PCR	SINDH	Karachi	NEGATIVE
221	2016	CCHF BY PCR	SINDH	Karachi	NEGATIVE

222	2016	CCHF BY PCR	SINDH	Karachi	NEGATIVE
223	2016	CCHF BY PCR	SINDH	Karachi	NEGATIVE
224	2016	CCHF BY PCR	SINDH	Karachi	POSITIVE
225	2016	CCHF BY PCR	SINDH	Karachi	NEGATIVE
226	2016	CCHF BY PCR	SINDH	Karachi	NEGATIVE
227	2016	CCHF BY PCR	SINDH	Karachi	POSITIVE
228	2016	CCHF BY PCR	SINDH	Karachi	NEGATIVE
229	2016	CCHF BY PCR	SINDH	Karachi	NEGATIVE
230	2016	CCHF BY PCR	SINDH	Karachi	NEGATIVE
231	2016	CCHF BY PCR	SINDH	Karachi	NEGATIVE
232	2016	CCHF BY PCR	SINDH	Karachi	NEGATIVE
233	2016	CCHF BY PCR	SINDH	Karachi	POSITIVE
234	2016	CCHF BY PCR	SINDH	Karachi	NEGATIVE
235	2016	CCHF BY PCR	SINDH	Karachi	POSITIVE
236	2016	CCHF BY PCR	SINDH	Karachi	POSITIVE
237	2016	CCHF BY PCR	SINDH	Karachi	NEGATIVE
238	2016	CCHF BY PCR	SINDH	Karachi	POSITIVE
239	2016	CCHF BY PCR	SINDH	Karachi	NEGATIVE
240	2016	CCHF BY PCR	SINDH	Karachi	POSITIVE
241	2016	CCHF BY PCR	SINDH	Karachi	POSITIVE
242	2016	CCHF BY PCR	SINDH	Karachi	NEGATIVE
243	2016	CCHF BY PCR	SINDH	Karachi	NEGATIVE
244	2016	CCHF BY PCR	SINDH	Karachi	NEGATIVE
245	2016	CCHF BY PCR	SINDH	Karachi	NEGATIVE
246	2016	CCHF BY PCR	SINDH	Karachi	NEGATIVE
247	2016	CCHF BY PCR	SINDH	Karachi	NEGATIVE
248	2016	CCHF BY PCR	SINDH	Karachi	NEGATIVE
249	2016	CCHF BY PCR	SINDH	Karachi	NEGATIVE
250	2016	CCHF BY PCR	SINDH	Karachi	NEGATIVE
251	2016	CCHF BY PCR	SINDH	Karachi	NEGATIVE
252	2016	CCHF BY PCR	SINDH	Karachi	NEGATIVE
253	2016	CCHF BY PCR	SINDH	Karachi	NEGATIVE
254	2016	CCHF BY PCR	SINDH	Karachi	NEGATIVE
255	2016	CCHF BY PCR	SINDH	Karachi	NEGATIVE
256	2016	CCHF BY PCR	SINDH	Karachi	NEGATIVE
257	2016	CCHF BY PCR	SINDH	Karachi	NEGATIVE
258	2016	CCHF BY PCR	SINDH	Karachi	NEGATIVE
259	2016	CCHF BY PCR	SINDH	Karachi	POSITIVE
260	2016	CCHF BY PCR	SINDH	Karachi	NEGATIVE
261	2016	CCHF BY PCR	SINDH	Karachi	NEGATIVE
262	2016	CCHF BY PCR	SINDH	Karachi	NEGATIVE
263	2016	CCHF BY PCR	SINDH	Karachi	NEGATIVE
264	2016	CCHF BY PCR	SINDH	Karachi	NEGATIVE
265	2016	CCHF BY PCR	SINDH	Karachi	NEGATIVE
266	2016	CCHF BY PCR	SINDH	Karachi	NEGATIVE
267	2016	CCHF BY PCR	SINDH	Karachi	NEGATIVE

268	2016	CCHF BY PCR	SINDH	Karachi	NEGATIVE
269	2016	CCHF BY PCR	SINDH	Karachi	NEGATIVE
270	2016	CCHF BY PCR	SINDH	Karachi	NEGATIVE
271	2016	CCHF BY PCR	SINDH	Karachi	NEGATIVE
272	2016	CCHF BY PCR	SINDH	Karachi	POSITIVE
273	2016	CCHF BY PCR	SINDH	Karachi	NEGATIVE
274	2016	CCHF BY PCR	SINDH	Karachi	NEGATIVE
275	2016	CCHF BY PCR	SINDH	Karachi	NEGATIVE
276	2016	CCHF BY PCR	SINDH	Karachi	NEGATIVE
277	2016	CCHF BY PCR	SINDH	Karachi	POSITIVE
278	2016	CCHF BY PCR	SINDH	Karachi	NEGATIVE
279	2017	CCHF BY PCR	SINDH	Karachi	NEGATIVE
280	2017	CCHF BY PCR	SINDH	Karachi	NEGATIVE
281	2017	CCHF BY PCR	SINDH	Karachi	NEGATIVE
282	2017	CCHF BY PCR	SINDH	Karachi	POSITIVE
283	2017	CCHF BY PCR	SINDH	Karachi	POSITIVE
284	2020	CCHF BY PCR	SINDH	Karachi	POSITIVE
285	2019	CCHF BY PCR	PUNJAB	Lahore	NEGATIVE
286	2017	CCHF BY PCR	SINDH	Karachi	NEGATIVE
287	2017	CCHF BY PCR	SINDH	Karachi	NEGATIVE
288	2017	CCHF BY PCR	SINDH	Karachi	NEGATIVE
289	2017	CCHF BY PCR	SINDH	Karachi	POSITIVE
290	2017	CCHF BY PCR	SINDH	Karachi	NEGATIVE
291	2017	CCHF BY PCR	SINDH	Karachi	NEGATIVE
292	2017	CCHF BY PCR	SINDH	Karachi	NEGATIVE
293	2017	CCHF BY PCR	SINDH	Karachi	POSITIVE
294	2017	CCHF BY PCR	SINDH	Karachi	NEGATIVE
295	2017	CCHF BY PCR	SINDH	Karachi	POSITIVE
296	2017	CCHF BY PCR	SINDH	Karachi	NEGATIVE
297	2017	CCHF BY PCR	SINDH	Karachi	NEGATIVE
298	2017	CCHF BY PCR	SINDH	Karachi	NEGATIVE
299	2017	CCHF BY PCR	SINDH	Karachi	POSITIVE
300	2017	CCHF BY PCR	SINDH	Karachi	NEGATIVE
301	2017	CCHF BY PCR	SINDH	Karachi	POSITIVE
302	2017	CCHF BY PCR	SINDH	Karachi	NEGATIVE
303	2017	CCHF BY PCR	SINDH	Karachi	NEGATIVE
304	2017	CCHF BY PCR	SINDH	Karachi	NEGATIVE
305	2017	CCHF BY PCR	SINDH	Karachi	NEGATIVE
306	2017	CCHF BY PCR	SINDH	Karachi	NEGATIVE
307	2017	CCHF BY PCR	SINDH	Karachi	NEGATIVE
308	2017	CCHF BY PCR	SINDH	Karachi	NEGATIVE
309	2017	CCHF BY PCR	SINDH	Karachi	NEGATIVE
310	2017	CCHF BY PCR	SINDH	Karachi	NEGATIVE
311	2017	CCHF BY PCR	SINDH	Karachi	NEGATIVE
312	2017	CCHF BY PCR	SINDH	Karachi	NEGATIVE
313	2017	CCHF BY PCR	SINDH	Karachi	NEGATIVE

314	2017	CCHF BY PCR	SINDH	Karachi	NEGATIVE
315	2017	CCHF BY PCR	SINDH	Karachi	NEGATIVE
316	2017	CCHF BY PCR	SINDH	Karachi	NEGATIVE
317	2017	CCHF BY PCR	SINDH	Karachi	NEGATIVE
318	2018	CCHF BY PCR	SINDH	Karachi	NEGATIVE
319	2018	CCHF BY PCR	SINDH	Karachi	NEGATIVE
320	2018	CCHF BY PCR	SINDH	Karachi	NEGATIVE
321	2018	CCHF BY PCR	SINDH	Karachi	NEGATIVE
322	2018	CCHF BY PCR	SINDH	Karachi	NEGATIVE
323	2018	CCHF BY PCR	SINDH	Karachi	NEGATIVE
324	2018	CCHF BY PCR	SINDH	Karachi	POSITIVE
325	2018	CCHF BY PCR	SINDH	Karachi	POSITIVE
326	2018	CCHF BY PCR	SINDH	Karachi	NEGATIVE
327	2018	CCHF BY PCR	SINDH	Karachi	NEGATIVE
328	2018	CCHF BY PCR	SINDH	Karachi	NEGATIVE
329	2018	CCHF BY PCR	SINDH	Karachi	NEGATIVE
330	2018	CCHF BY PCR	SINDH	Karachi	NEGATIVE
331	2018	CCHF BY PCR	SINDH	Karachi	NEGATIVE
332	2018	CCHF BY PCR	SINDH	Karachi	POSITIVE
333	2018	CCHF BY PCR	SINDH	Karachi	NEGATIVE
334	2018	CCHF BY PCR	SINDH	Karachi	NEGATIVE
335	2018	CCHF BY PCR	SINDH	Karachi	NEGATIVE
336	2018	CCHF BY PCR	SINDH	Karachi	POSITIVE
337	2018	CCHF BY PCR	SINDH	Karachi	POSITIVE
338	2018	CCHF BY PCR	SINDH	Karachi	NEGATIVE
339	2018	CCHF BY PCR	SINDH	Karachi	NEGATIVE
340	2018	CCHF BY PCR	SINDH	Karachi	POSITIVE
341	2018	CCHF BY PCR	SINDH	Karachi	NEGATIVE
342	2018	CCHF BY PCR	SINDH	Karachi	POSITIVE
343	2018	CCHF BY PCR	SINDH	Karachi	POSITIVE
344	2018	CCHF BY PCR	SINDH	Karachi	POSITIVE
345	2018	CCHF BY PCR	SINDH	Karachi	NEGATIVE
346	2018	CCHF BY PCR	SINDH	Karachi	NEGATIVE
347	2018	CCHF BY PCR	SINDH	Karachi	POSITIVE
348	2018	CCHF BY PCR	SINDH	Karachi	POSITIVE
349	2018	CCHF BY PCR	SINDH	Karachi	POSITIVE
350	2018	CCHF BY PCR	SINDH	Karachi	NEGATIVE
351	2018	CCHF BY PCR	SINDH	Karachi	NEGATIVE
352	2018	CCHF BY PCR	SINDH	Karachi	NEGATIVE
353	2018	CCHF BY PCR	SINDH	Karachi	POSITIVE
354	2018	CCHF BY PCR	SINDH	Karachi	NEGATIVE
355	2018	CCHF BY PCR	SINDH	Karachi	NEGATIVE
356	2018	CCHF BY PCR	SINDH	Karachi	POSITIVE
357	2018	CCHF BY PCR	SINDH	Karachi	NEGATIVE
358	2018	CCHF BY PCR	SINDH	Karachi	NEGATIVE
359	2018	CCHF BY PCR	SINDH	Karachi	NEGATIVE

360	2018	CCHF BY PCR	SINDH	Karachi	POSITIVE
361	2018	CCHF BY PCR	SINDH	Karachi	POSITIVE
362	2018	CCHF BY PCR	SINDH	Karachi	NEGATIVE
363	2018	CCHF BY PCR	SINDH	Karachi	NEGATIVE
364	2018	CCHF BY PCR	SINDH	Karachi	NEGATIVE
365	2018	CCHF BY PCR	SINDH	Karachi	NEGATIVE
366	2018	CCHF BY PCR	SINDH	Karachi	NEGATIVE
367	2019	CCHF BY PCR	SINDH	Karachi	NEGATIVE
368	2019	CCHF BY PCR	SINDH	Karachi	NEGATIVE
369	2019	CCHF BY PCR	SINDH	Karachi	NEGATIVE
370	2019	CCHF BY PCR	SINDH	Karachi	NEGATIVE
371	2019	CCHF BY PCR	SINDH	Karachi	NEGATIVE
372	2019	CCHF BY PCR	SINDH	Karachi	NEGATIVE
373	2019	CCHF BY PCR	SINDH	Karachi	NEGATIVE
374	2019	CCHF BY PCR	SINDH	Karachi	NEGATIVE
375	2021	CCHF BY PCR	SINDH	Karachi	NEGATIVE
376	2019	CCHF BY PCR	SINDH	Karachi	NEGATIVE
377	2015	CCHF BY PCR	SINDH	Karachi	NEGATIVE
378	2016	CCHF BY PCR	SINDH	Karachi	NEGATIVE
379	2016	CCHF BY PCR	SINDH	Karachi	NEGATIVE
380	2015	CCHF BY PCR	SINDH	Karachi	NEGATIVE
381	2015	CCHF BY PCR	SINDH	Karachi	NEGATIVE
382	2015	CCHF BY PCR	SINDH	Karachi	NEGATIVE
383	2017	CCHF BY PCR	SINDH	Karachi	POSITIVE
384	2019	CCHF BY PCR	SINDH	Karachi	NEGATIVE
385	2016	CCHF BY PCR	SINDH	Karachi	NEGATIVE
386	2016	CCHF BY PCR	SINDH	Karachi	NEGATIVE
387	2018	CCHF BY PCR	SINDH	Karachi	NEGATIVE
388	2016	CCHF BY PCR	SINDH	Karachi	NEGATIVE
389	2019	CCHF BY PCR	SINDH	Karachi	NEGATIVE
390	2018	CCHF BY PCR	SINDH	Karachi	NEGATIVE
391	2017	CCHF BY PCR	SINDH	Karachi	POSITIVE
392	2015	CCHF BY PCR	SINDH	Karachi	NEGATIVE
393	2016	CCHF BY PCR	SINDH	Karachi	NEGATIVE
394	2018	CCHF BY PCR	SINDH	Karachi	NEGATIVE
395	2018	CCHF BY PCR	SINDH	Karachi	NEGATIVE
396	2016	CCHF BY PCR	SINDH	Karachi	NEGATIVE
397	2017	CCHF BY PCR	SINDH	Karachi	NEGATIVE
398	2022	CCHF BY PCR	SINDH	Karachi	NEGATIVE
399	2013	CCHF BY PCR	SINDH	Karachi	NEGATIVE
400	2022	CCHF BY PCR	SINDH	Karachi	NEGATIVE
401	2016	CCHF BY PCR	SINDH	Karachi	NEGATIVE
402	2016	CCHF BY PCR	SINDH	Karachi	NEGATIVE
403	2014	CCHF BY PCR	SINDH	Karachi	NEGATIVE
404	2012	CCHF BY PCR	SINDH	Karachi	POSITIVE
405	2012	CCHF BY PCR	SINDH	Karachi	NEGATIVE

406	2016	CCHF BY PCR	SINDH	Karachi	NEGATIVE
407	2019	CCHF BY PCR	SINDH	Karachi	NEGATIVE
408	2014	CCHF BY PCR	SINDH	Karachi	NEGATIVE
409	2020	CCHF BY PCR	SINDH	Karachi	NEGATIVE
410	2019	CCHF BY PCR	SINDH	Karachi	NEGATIVE
411	2012	CCHF BY PCR	SINDH	Karachi	NEGATIVE
412	2014	CCHF BY PCR	SINDH	Karachi	NEGATIVE
413	2014	CCHF BY PCR	SINDH	Karachi	NEGATIVE
414	2013	CCHF BY PCR	SINDH	Karachi	NEGATIVE
415	2014	CCHF BY PCR	SINDH	Karachi	NEGATIVE
416	2014	CCHF BY PCR	SINDH	Karachi	NEGATIVE
417	2014	CCHF BY PCR	SINDH	Karachi	NEGATIVE
418	2017	CCHF BY PCR	SINDH	Karachi	NEGATIVE
419	2016	CCHF BY PCR	SINDH	Karachi	NEGATIVE
420	2018	CCHF BY PCR	SINDH	Karachi	NEGATIVE
421	2014	CCHF BY PCR	SINDH	Karachi	NEGATIVE
422	2015	CCHF BY PCR	SINDH	Karachi	NEGATIVE
423	2018	CCHF BY PCR	SINDH	Karachi	NEGATIVE
424	2018	CCHF BY PCR	SINDH	Karachi	NEGATIVE
425	2019	CCHF BY PCR	SINDH	Karachi	NEGATIVE
426	2016	CCHF BY PCR	SINDH	Karachi	NEGATIVE
427	2019	CCHF BY PCR	SINDH	Karachi	NEGATIVE
428	2016	CCHF BY PCR	SINDH	Karachi	NEGATIVE
429	2016	CCHF BY PCR	SINDH	Karachi	NEGATIVE
430	2015	CCHF BY PCR	SINDH	Karachi	NEGATIVE
431	2015	CCHF BY PCR	SINDH	Karachi	NEGATIVE
432	2017	CCHF BY PCR	SINDH	Karachi	NEGATIVE
433	2019	CCHF BY PCR	SINDH	Karachi	POSITIVE
434	2016	CCHF BY PCR	SINDH	Karachi	NEGATIVE
435	2022	CCHF BY PCR	SINDH	Karachi	NEGATIVE
436	2018	CCHF BY PCR	SINDH	Karachi	NEGATIVE
437	2016	CCHF BY PCR	SINDH	Karachi	NEGATIVE
438	2016	CCHF BY PCR	SINDH	Karachi	NEGATIVE
439	2016	CCHF BY PCR	SINDH	Karachi	POSITIVE
440	2016	CCHF BY PCR	PUNJAB	Bahawalpur	POSITIVE
441	2016	CCHF BY PCR	SINDH	Karachi	NEGATIVE
442	2016	CCHF BY PCR	SINDH	Karachi	NEGATIVE
443	2018	CCHF BY PCR	SINDH	Karachi	NEGATIVE
444	2016	CCHF BY PCR	SINDH	Karachi	NEGATIVE
445	2016	CCHF BY PCR	SINDH	Karachi	NEGATIVE
446	2019	CCHF BY PCR	SINDH	Karachi	NEGATIVE
447	2017	CCHF BY PCR	SINDH	Karachi	NEGATIVE
448	2018	CCHF BY PCR	SINDH	Karachi	NEGATIVE
449	2017	CCHF BY PCR	SINDH	Karachi	NEGATIVE
450	2018	CCHF BY PCR	SINDH	Karachi	NEGATIVE
451	2017	CCHF BY PCR	SINDH	Karachi	NEGATIVE

452	2022	CCHF BY PCR	SINDH	Karachi	NEGATIVE
453	2020	CCHF BY PCR	SINDH	Karachi	NEGATIVE
454	2019	CCHF BY PCR	SINDH	Karachi	POSITIVE
455	2019	CCHF BY PCR	SINDH	Karachi	NEGATIVE
456	2021	CCHF BY PCR	SINDH	Karachi	NEGATIVE
457	2019	CCHF BY PCR	SINDH	Karachi	NEGATIVE
458	2019	CCHF BY PCR	SINDH	Karachi	POSITIVE
459	2019	CCHF BY PCR	SINDH	Karachi	NEGATIVE
460	2019	CCHF BY PCR	SINDH	Karachi	POSITIVE
461	2019	CCHF BY PCR	SINDH	Karachi	POSITIVE
462	2019	CCHF BY PCR	SINDH	Karachi	POSITIVE
463	2019	CCHF BY PCR	SINDH	Karachi	NEGATIVE
464	2019	CCHF BY PCR	SINDH	Karachi	NEGATIVE
465	2020	CCHF BY PCR	SINDH	Karachi	NEGATIVE
466	2019	CCHF BY PCR	SINDH	Karachi	NEGATIVE
467	2019	CCHF BY PCR	SINDH	Karachi	NEGATIVE
468	2019	CCHF BY PCR	SINDH	Karachi	NEGATIVE
469	2019	CCHF BY PCR	SINDH	Karachi	POSITIVE
470	2019	CCHF BY PCR	SINDH	Karachi	NEGATIVE
471	2019	CCHF BY PCR	SINDH	Karachi	POSITIVE
472	2019	CCHF BY PCR	SINDH	Karachi	NEGATIVE
473	2019	CCHF BY PCR	SINDH	Karachi	POSITIVE
474	2019	CCHF BY PCR	SINDH	Karachi	NEGATIVE
475	2019	CCHF BY PCR	SINDH	Karachi	NEGATIVE
476	2019	CCHF BY PCR	SINDH	Karachi	POSITIVE
477	2019	CCHF BY PCR	SINDH	Karachi	POSITIVE
478	2019	CCHF BY PCR	SINDH	Karachi	NEGATIVE
479	2019	CCHF BY PCR	SINDH	Karachi	NEGATIVE
480	2019	CCHF BY PCR	SINDH	Karachi	NEGATIVE
481	2019	CCHF BY PCR	SINDH	Karachi	NEGATIVE
482	2019	CCHF BY PCR	SINDH	Karachi	POSITIVE
483	2019	CCHF BY PCR	SINDH	Karachi	POSITIVE
484	2019	CCHF BY PCR	SINDH	Karachi	POSITIVE
485	2019	CCHF BY PCR	SINDH	Karachi	NEGATIVE
486	2019	CCHF BY PCR	SINDH	Karachi	NEGATIVE
487	2019	CCHF BY PCR	SINDH	Karachi	POSITIVE
488	2019	CCHF BY PCR	SINDH	Karachi	NEGATIVE
489	2019	CCHF BY PCR	SINDH	Karachi	NEGATIVE
490	2019	CCHF BY PCR	SINDH	Karachi	NEGATIVE
491	2019	CCHF BY PCR	SINDH	Karachi	NEGATIVE
492	2019	CCHF BY PCR	SINDH	Karachi	NEGATIVE
493	2019	CCHF BY PCR	SINDH	Karachi	NEGATIVE
494	2019	CCHF BY PCR	SINDH	Karachi	POSITIVE
495	2019	CCHF BY PCR	SINDH	Karachi	NEGATIVE
496	2019	CCHF BY PCR	SINDH	Karachi	NEGATIVE
497	2019	CCHF BY PCR	SINDH	Karachi	NEGATIVE

498	2019	CCHF BY PCR	SINDH	Karachi	POSITIVE
499	2019	CCHF BY PCR	SINDH	Karachi	POSITIVE
500	2019	CCHF BY PCR	SINDH	Karachi	NEGATIVE
501	2019	CCHF BY PCR	SINDH	Karachi	NEGATIVE
502	2019	CCHF BY PCR	SINDH	Karachi	POSITIVE
503	2019	CCHF BY PCR	SINDH	Karachi	NEGATIVE
504	2020	CCHF BY PCR	SINDH	Karachi	POSITIVE
505	2020	CCHF BY PCR	SINDH	Karachi	NEGATIVE
506	2020	CCHF BY PCR	SINDH	Karachi	NEGATIVE
507	2020	CCHF BY PCR	SINDH	Karachi	NEGATIVE
508	2020	CCHF BY PCR	SINDH	Karachi	NEGATIVE
509	2020	CCHF BY PCR	SINDH	Karachi	NEGATIVE
510	2020	CCHF BY PCR	SINDH	Karachi	POSITIVE
511	2020	CCHF BY PCR	SINDH	Karachi	POSITIVE
512	2020	CCHF BY PCR	SINDH	Karachi	POSITIVE
513	2020	CCHF BY PCR	SINDH	Karachi	NEGATIVE
514	2020	CCHF BY PCR	SINDH	Karachi	NEGATIVE
515	2020	CCHF BY PCR	SINDH	Karachi	NEGATIVE
516	2020	CCHF BY PCR	SINDH	Karachi	NEGATIVE
517	2020	CCHF BY PCR	SINDH	Karachi	NEGATIVE
518	2020	CCHF BY PCR	SINDH	Karachi	POSITIVE
519	2020	CCHF BY PCR	SINDH	Karachi	NEGATIVE
520	2021	CCHF BY PCR	SINDH	Karachi	NEGATIVE
521	2021	CCHF BY PCR	SINDH	Karachi	NEGATIVE
522	2021	CCHF BY PCR	SINDH	Karachi	NEGATIVE
523	2021	CCHF BY PCR	SINDH	Karachi	NEGATIVE
524	2021	CCHF BY PCR	SINDH	Karachi	NEGATIVE
525	2021	CCHF BY PCR	SINDH	Karachi	NEGATIVE
526	2023	CCHF BY PCR	SINDH	Karachi	NEGATIVE
527	2021	CCHF BY PCR	SINDH	Karachi	NEGATIVE
528	2021	CCHF BY PCR	SINDH	Karachi	POSITIVE
529	2021	CCHF BY PCR	SINDH	Karachi	NEGATIVE
530	2021	CCHF BY PCR	SINDH	Karachi	NEGATIVE
531	2021	CCHF BY PCR	SINDH	Karachi	POSITIVE
532	2021	CCHF BY PCR	SINDH	Karachi	POSITIVE
533	2021	CCHF BY PCR	SINDH	Karachi	POSITIVE
534	2021	CCHF BY PCR	SINDH	Karachi	POSITIVE
535	2021	CCHF BY PCR	SINDH	Karachi	NEGATIVE
536	2021	CCHF BY PCR	SINDH	Karachi	NEGATIVE
537	2021	CCHF BY PCR	SINDH	Karachi	NEGATIVE
538	2021	CCHF BY PCR	SINDH	Karachi	NEGATIVE
539	2022	CCHF BY PCR	SINDH	Karachi	NEGATIVE
540	2022	CCHF BY PCR	SINDH	Karachi	NEGATIVE
541	2022	CCHF BY PCR	SINDH	Karachi	NEGATIVE
542	2022	CCHF BY PCR	SINDH	Karachi	NEGATIVE
543	2022	CCHF BY PCR	SINDH	Karachi	POSITIVE

544	2022	CCHF BY PCR	SINDH	Karachi	NEGATIVE
545	2022	CCHF BY PCR	SINDH	Karachi	NEGATIVE
546	2022	CCHF BY PCR	SINDH	Karachi	NEGATIVE
547	2022	CCHF BY PCR	SINDH	Karachi	NEGATIVE
548	2022	CCHF BY PCR	SINDH	Karachi	NEGATIVE
549	2022	CCHF BY PCR	SINDH	Karachi	POSITIVE
550	2022	CCHF BY PCR	SINDH	Karachi	NEGATIVE
551	2022	CCHF BY PCR	SINDH	Karachi	NEGATIVE
552	2022	CCHF BY PCR	SINDH	Karachi	NEGATIVE
553	2022	CCHF BY PCR	SINDH	Karachi	NEGATIVE
554	2022	CCHF BY PCR	SINDH	Karachi	NEGATIVE
555	2022	CCHF BY PCR	SINDH	Karachi	POSITIVE
556	2022	CCHF BY PCR	SINDH	Karachi	NEGATIVE
557	2022	CCHF BY PCR	SINDH	Karachi	POSITIVE
558	2022	CCHF BY PCR	SINDH	Karachi	POSITIVE
559	2022	CCHF BY PCR	SINDH	Karachi	NEGATIVE
560	2022	CCHF BY PCR	SINDH	Karachi	NEGATIVE
561	2022	CCHF BY PCR	SINDH	Karachi	POSITIVE
562	2022	CCHF BY PCR	SINDH	Karachi	NEGATIVE
563	2022	CCHF BY PCR	SINDH	Karachi	POSITIVE
564	2022	CCHF BY PCR	SINDH	Karachi	POSITIVE
565	2022	CCHF BY PCR	SINDH	Karachi	POSITIVE
566	2022	CCHF BY PCR	SINDH	Karachi	NEGATIVE
567	2022	CCHF BY PCR	SINDH	Karachi	NEGATIVE
568	2022	CCHF BY PCR	SINDH	Karachi	NEGATIVE
569	2022	CCHF BY PCR	SINDH	Karachi	NEGATIVE
570	2022	CCHF BY PCR	SINDH	Karachi	NEGATIVE
571	2022	CCHF BY PCR	SINDH	Karachi	NEGATIVE
572	2022	CCHF BY PCR	SINDH	Karachi	NEGATIVE
573	2022	CCHF BY PCR	SINDH	Karachi	NEGATIVE
574	2022	CCHF BY PCR	SINDH	Karachi	NEGATIVE
575	2023	CCHF BY PCR	SINDH	Karachi	NEGATIVE
576	2023	CCHF BY PCR	SINDH	Karachi	POSITIVE
577	2023	CCHF BY PCR	SINDH	Karachi	NEGATIVE
578	2023	CCHF BY PCR	SINDH	Karachi	NEGATIVE
579	2023	CCHF BY PCR	SINDH	Karachi	NEGATIVE
580	2023	CCHF BY PCR	SINDH	Karachi	NEGATIVE
581	2018	CCHF BY PCR	SINDH	Karachi	NEGATIVE
582	2019	CCHF BY PCR	SINDH	Karachi	NEGATIVE
583	2022	CCHF BY PCR	SINDH	Karachi	NEGATIVE
584	2021	CCHF BY PCR	SINDH	Karachi	NEGATIVE
585	2018	CCHF BY PCR	SINDH	Karachi	NEGATIVE
586	2018	CCHF BY PCR	SINDH	Karachi	NEGATIVE
587	2022	CCHF BY PCR	SINDH	Karachi	NEGATIVE
588	2022	CCHF BY PCR	SINDH	Karachi	POSITIVE
589	2022	CCHF BY PCR	SINDH	Karachi	NEGATIVE

590	2021	CCHF BY PCR	SINDH	Karachi	NEGATIVE
591	2019	CCHF BY PCR	SINDH	Karachi	NEGATIVE
592	2019	CCHF BY PCR	SINDH	Karachi	NEGATIVE
593	2019	CCHF BY PCR	SINDH	Karachi	NEGATIVE
594	2019	CCHF BY PCR	SINDH	Karachi	NEGATIVE
595	2021	CCHF BY PCR	SINDH	Karachi	NEGATIVE
596	2021	CCHF BY PCR	SINDH	Karachi	NEGATIVE
597	2022	CCHF BY PCR	SINDH	Karachi	NEGATIVE
598	2022	CCHF BY PCR	SINDH	Karachi	NEGATIVE
599	2016	CCHF BY PCR	SINDH	Karachi	NEGATIVE
600	2012	CCHF BY PCR	SINDH	Karachi	NEGATIVE
601	2021	CCHF BY PCR	SINDH	Karachi	POSITIVE
602	2014	CCHF BY PCR	SINDH	Karachi	NEGATIVE
603	2016	CCHF BY PCR	SINDH	Hyderabad	NEGATIVE
604	2019	CCHF BY PCR	SINDH	Hyderabad	NEGATIVE
605	2016	CCHF BY PCR	SINDH	Karachi	NEGATIVE
606	2013	CCHF BY PCR	SINDH	Karachi	NEGATIVE
607	2014	CCHF BY PCR	SINDH	Karachi	NEGATIVE
608	2015	CCHF BY PCR	SINDH	Karachi	NEGATIVE
609	2018	CCHF BY PCR	SINDH	Karachi	NEGATIVE
610	2022	CCHF BY PCR	SINDH	Karachi	NEGATIVE
611	2022	CCHF BY PCR	SINDH	Karachi	NEGATIVE
612	2014	CCHF BY PCR	SINDH	Karachi	NEGATIVE
613	2012	CCHF BY PCR	SINDH	Karachi	POSITIVE
614	2012	CCHF BY PCR	PUNJAB	Islamabad	NEGATIVE
615	2012	CCHF BY PCR	SINDH	Karachi	NEGATIVE
616	2012	CCHF BY PCR	SINDH	Karachi	NEGATIVE
617	2012	CCHF BY PCR	SINDH	Karachi	NEGATIVE
618	2012	CCHF BY PCR	SINDH	Karachi	POSITIVE
619	2016	CCHF BY PCR	SINDH	Karachi	NEGATIVE
620	2012	CCHF BY PCR	SINDH	Karachi	POSITIVE
621	2012	CCHF BY PCR	SINDH	Karachi	NEGATIVE
622	2012	CCHF BY PCR	SINDH	Karachi	NEGATIVE
623	2012	CCHF BY PCR	SINDH	Karachi	POSITIVE
624	2012	CCHF BY PCR	SINDH	Karachi	NEGATIVE
625	2012	CCHF BY PCR	Baluchistan	Quetta	POSITIVE
626	2012	CCHF BY PCR	SINDH	Karachi	NEGATIVE
627	2012	CCHF BY PCR	SINDH	Karachi	NEGATIVE
628	2012	CCHF BY PCR	Baluchistan	Quetta	NEGATIVE
629	2012	CCHF BY PCR	PUNJAB	Islamabad	NEGATIVE
630	2012	CCHF BY PCR	SINDH	Karachi	NEGATIVE
631	2012	CCHF BY PCR	Baluchistan	Quetta	NEGATIVE
632	2012	CCHF BY PCR	KPK	Abbottabad	NEGATIVE
633	2012	CCHF BY PCR	SINDH	Karachi	NEGATIVE

634	2012	CCHF BY PCR	SINDH	Karachi	NEGATIVE
635	2012	CCHF BY PCR	SINDH	Karachi	POSITIVE
636	2012	CCHF BY PCR	SINDH	Karachi	POSITIVE
637	2012	CCHF BY PCR	PUNJAB	Islamabad	NEGATIVE
638	2012	CCHF BY PCR	SINDH	Karachi	NEGATIVE
639	2012	CCHF BY PCR	SINDH	Karachi	NEGATIVE
640	2012	CCHF BY PCR	SINDH	Karachi	NEGATIVE
641	2012	CCHF BY PCR	SINDH	Karachi	NEGATIVE
642	2012	CCHF BY PCR	SINDH	Karachi	NEGATIVE
643	2012	CCHF BY PCR	SINDH	Karachi	NEGATIVE
644	2013	CCHF BY PCR	SINDH	Karachi	NEGATIVE
645	2013	CCHF BY PCR	PUNJAB	MULTAN	NEGATIVE
646	2013	CCHF BY PCR	SINDH	Karachi	NEGATIVE
647	2013	CCHF BY PCR	PUNJAB	Rawalpindi	POSITIVE
648	2013	CCHF BY PCR	SINDH	Karachi	POSITIVE
649	2013	CCHF BY PCR	SINDH	Karachi	NEGATIVE
650	2013	CCHF BY PCR	SINDH	Karachi	POSITIVE
651	2013	CCHF BY PCR	SINDH	Karachi	POSITIVE
652	2013	CCHF BY PCR	SINDH	Karachi	NEGATIVE
653	2013	CCHF BY PCR	SINDH	Karachi	NEGATIVE
654	2013	CCHF BY PCR	SINDH	Karachi	NEGATIVE
655	2013	CCHF BY PCR	SINDH	Karachi	NEGATIVE
656	2013	CCHF BY PCR	KPK	Peshawar	POSITIVE
657	2013	CCHF BY PCR	SINDH	Karachi	NEGATIVE
658	2013	CCHF BY PCR	SINDH	Karachi	NEGATIVE
659	2013	CCHF BY PCR	SINDH	Karachi	POSITIVE
660	2013	CCHF BY PCR	SINDH	Karachi	NEGATIVE
661	2013	CCHF BY PCR	KPK	Peshawar	NEGATIVE
662	2013	CCHF BY PCR	SINDH	Karachi	NEGATIVE
663	2013	CCHF BY PCR	SINDH	Karachi	NEGATIVE
664	2013	CCHF BY PCR	SINDH	Karachi	NEGATIVE
665	2013	CCHF BY PCR	SINDH	Karachi	NEGATIVE
666	2013	CCHF BY PCR	SINDH	Karachi	NEGATIVE
667	2013	CCHF BY PCR	SINDH	Karachi	NEGATIVE
668	2013	CCHF BY PCR	SINDH	Karachi	NEGATIVE
669	2013	CCHF BY PCR	SINDH	Karachi	NEGATIVE
670	2013	CCHF BY PCR	SINDH	Karachi	NEGATIVE
671	2013	CCHF BY PCR	SINDH	Karachi	NEGATIVE
672	2013	CCHF BY PCR	Baluchistan	Quetta	POSITIVE
673	2013	CCHF BY PCR	SINDH	Karachi	NEGATIVE
674	2013	CCHF BY PCR	PUNJAB	Rawalpindi	POSITIVE
675	2013	CCHF BY PCR	PUNJAB	Rawalpindi	POSITIVE
676	2013	CCHF BY PCR	SINDH	Karachi	POSITIVE
677	2013	CCHF BY PCR	PUNJAB	Islamabad	NEGATIVE
678	2013	CCHF BY PCR	KPK	Peshawar	POSITIVE

679	2013	CCHF BY PCR	SINDH	Karachi	NEGATIVE
680	2013	CCHF BY PCR	SINDH	Karachi	NEGATIVE
681	2013	CCHF BY PCR	SINDH	Karachi	NEGATIVE
682	2013	CCHF BY PCR	SINDH	Karachi	NEGATIVE
683	2013	CCHF BY PCR	KPK	Peshawar	NEGATIVE
684	2013	CCHF BY PCR	KPK	Peshawar	POSITIVE
685	2013	CCHF BY PCR	SINDH	Karachi	NEGATIVE
686	2013	CCHF BY PCR	KPK	Peshawar	NEGATIVE
687	2013	CCHF BY PCR	SINDH	Karachi	NEGATIVE
688	2013	CCHF BY PCR	SINDH	Karachi	NEGATIVE
689	2013	CCHF BY PCR	KPK	Peshawar	POSITIVE
690	2013	CCHF BY PCR	SINDH	Karachi	NEGATIVE
691	2013	CCHF BY PCR	KPK	Peshawar	POSITIVE
692	2013	CCHF BY PCR	SINDH	Karachi	NEGATIVE
693	2013	CCHF BY PCR	KPK	Peshawar	NEGATIVE
694	2013	CCHF BY PCR	KPK	Peshawar	NEGATIVE
695	2013	CCHF BY PCR	KPK	Peshawar	POSITIVE
696	2013	CCHF BY PCR	KPK	Peshawar	POSITIVE
697	2013	CCHF BY PCR	KPK	Peshawar	NEGATIVE
698	2013	CCHF BY PCR	SINDH	Karachi	NEGATIVE
699	2013	CCHF BY PCR	KPK	Peshawar	NEGATIVE
700	2013	CCHF BY PCR	SINDH	Karachi	NEGATIVE
701	2013	CCHF BY PCR	KPK	Peshawar	NEGATIVE
702	2013	CCHF BY PCR	KPK	Peshawar	POSITIVE
703	2013	CCHF BY PCR	KPK	Peshawar	NEGATIVE
704	2013	CCHF BY PCR	KPK	Peshawar	POSITIVE
705	2013	CCHF BY PCR	SINDH	Karachi	NEGATIVE
706	2013	CCHF BY PCR	PUNJAB	Islamabad	NEGATIVE
707	2013	CCHF BY PCR	KPK	Peshawar	NEGATIVE
708	2013	CCHF BY PCR	SINDH	Karachi	NEGATIVE
709	2013	CCHF BY PCR	KPK	Peshawar	NEGATIVE
710	2013	CCHF BY PCR	SINDH	Karachi	NEGATIVE
711	2013	CCHF BY PCR	SINDH	Karachi	NEGATIVE
712	2013	CCHF BY PCR	KPK	Peshawar	NEGATIVE
713	2013	CCHF BY PCR	PUNJAB	Rawalpindi	NEGATIVE
714	2013	CCHF BY PCR	SINDH	Karachi	NEGATIVE
715	2013	CCHF BY PCR	PUNJAB	Rawalpindi	NEGATIVE
716	2013	CCHF BY PCR	PUNJAB	Lahore	NEGATIVE
717	2013	CCHF BY PCR	KPK	Peshawar	NEGATIVE
718	2013	CCHF BY PCR	SINDH	Karachi	NEGATIVE
719	2013	CCHF BY PCR	PUNJAB	Islamabad	NEGATIVE
720	2013	CCHF BY PCR	PUNJAB	MULTAN	POSITIVE
721	2013	CCHF BY PCR	SINDH	Karachi	NEGATIVE
722	2013	CCHF BY PCR	SINDH	Karachi	NEGATIVE
723	2013	CCHF BY PCR	SINDH	Karachi	NEGATIVE
724	2013	CCHF BY PCR	SINDH	Karachi	NEGATIVE

725	2014	CCHF BY PCR	Baluchistan	Quetta	NEGATIVE
726	2014	CCHF BY PCR	KPK	Peshawar	NEGATIVE
727	2018	CCHF BY PCR	PUNJAB	Lahore	NEGATIVE
728	2014	CCHF BY PCR	SINDH	Karachi	NEGATIVE
729	2014	CCHF BY PCR	SINDH	Karachi	NEGATIVE
730	2014	CCHF BY PCR	SINDH	Karachi	NEGATIVE
731	2014	CCHF BY PCR	Baluchistan	Quetta	NEGATIVE
732	2016	CCHF BY PCR	PUNJAB	Bahawalpur	NEGATIVE
733	2014	CCHF BY PCR	PUNJAB	MULTAN	NEGATIVE
734	2014	CCHF BY PCR	PUNJAB	MULTAN	NEGATIVE
735	2014	CCHF BY PCR	Baluchistan	Quetta	NEGATIVE
736	2014	CCHF BY PCR	SINDH	Karachi	NEGATIVE
737	2014	CCHF BY PCR	KPK	Peshawar	POSITIVE
738	2014	CCHF BY PCR	KPK	Peshawar	POSITIVE
739	2014	CCHF BY PCR	SINDH	Karachi	NEGATIVE
740	2014	CCHF BY PCR	Baluchistan	Quetta	POSITIVE
741	2014	CCHF BY PCR	PUNJAB	MULTAN	NEGATIVE
742	2014	CCHF BY PCR	Baluchistan	Quetta	NEGATIVE
743	2014	CCHF BY PCR	KPK	Peshawar	POSITIVE
744	2014	CCHF BY PCR	KPK	Peshawar	NEGATIVE
745	2014	CCHF BY PCR	SINDH	Karachi	NEGATIVE
746	2014	CCHF BY PCR	SINDH	Karachi	NEGATIVE
747	2014	CCHF BY PCR	KPK	Peshawar	POSITIVE
748	2014	CCHF BY PCR	KPK	Peshawar	POSITIVE
749	2014	CCHF BY PCR	KPK	Peshawar	POSITIVE
750	2014	CCHF BY PCR	KPK	Peshawar	POSITIVE
751	2014	CCHF BY PCR	KPK	Peshawar	NEGATIVE
752	2014	CCHF BY PCR	KPK	Peshawar	NEGATIVE
753	2014	CCHF BY PCR	KPK	Peshawar	POSITIVE
754	2014	CCHF BY PCR	SINDH	Karachi	NEGATIVE
755	2014	CCHF BY PCR	KPK	Peshawar	NEGATIVE
756	2014	CCHF BY PCR	Baluchistan	Quetta	NEGATIVE
757	2014	CCHF BY PCR	KPK	Peshawar	NEGATIVE
758	2014	CCHF BY PCR	KPK	Peshawar	NEGATIVE
759	2014	CCHF BY PCR	KPK	Peshawar	NEGATIVE
760	2014	CCHF BY PCR	SINDH	Karachi	POSITIVE
761	2014	CCHF BY PCR	KPK	Peshawar	NEGATIVE
762	2014	CCHF BY PCR	KPK	Peshawar	POSITIVE
763	2014	CCHF BY PCR	KPK	Peshawar	POSITIVE
764	2014	CCHF BY PCR	KPK	Peshawar	NEGATIVE
765	2014	CCHF BY PCR	KPK	Peshawar	NEGATIVE

766	2014	CCHF BY PCR	SINDH	Karachi	NEGATIVE
767	2014	CCHF BY PCR	SINDH	Karachi	NEGATIVE
768	2014	CCHF BY PCR	SINDH	Karachi	POSITIVE
769	2014	CCHF BY PCR	KPK	Peshawar	POSITIVE
770	2014	CCHF BY PCR	KPK	Peshawar	NEGATIVE
771	2014	CCHF BY PCR	SINDH	Karachi	NEGATIVE
772	2014	CCHF BY PCR	KPK	Peshawar	NEGATIVE
773	2014	CCHF BY PCR	KPK	Peshawar	POSITIVE
774	2014	CCHF BY PCR	SINDH	Karachi	POSITIVE
775	2014	CCHF BY PCR	KPK	Peshawar	NEGATIVE
776	2014	CCHF BY PCR	KPK	Peshawar	POSITIVE
777	2014	CCHF BY PCR	SINDH	Karachi	POSITIVE
778	2014	CCHF BY PCR	SINDH	Karachi	NEGATIVE
779	2014	CCHF BY PCR	Baluchistan	Quetta	NEGATIVE
780	2014	CCHF BY PCR	SINDH	Karachi	NEGATIVE
781	2014	CCHF BY PCR	KPK	Peshawar	NEGATIVE
782	2014	CCHF BY PCR	SINDH	Karachi	POSITIVE
783	2014	CCHF BY PCR	KPK	Peshawar	POSITIVE
784	2014	CCHF BY PCR	KPK	Peshawar	NEGATIVE
785	2014	CCHF BY PCR	KPK	Peshawar	NEGATIVE
786	2014	CCHF BY PCR	SINDH	Karachi	NEGATIVE
787	2014	CCHF BY PCR	SINDH	Karachi	NEGATIVE
788	2014	CCHF BY PCR	KPK	Peshawar	NEGATIVE
789	2014	CCHF BY PCR	SINDH	Karachi	POSITIVE
790	2014	CCHF BY PCR	SINDH	Karachi	NEGATIVE
791	2014	CCHF BY PCR	KPK	Peshawar	NEGATIVE
792	2014	CCHF BY PCR	SINDH	Karachi	NEGATIVE
793	2014	CCHF BY PCR	KPK	Peshawar	POSITIVE
794	2014	CCHF BY PCR	SINDH	Karachi	NEGATIVE
795	2014	CCHF BY PCR	KPK	Peshawar	NEGATIVE
796	2014	CCHF BY PCR	SINDH	Karachi	NEGATIVE
797	2014	CCHF BY PCR	SINDH	Karachi	NEGATIVE
798	2014	CCHF BY PCR	SINDH	Karachi	POSITIVE
799	2014	CCHF BY PCR	SINDH	Karachi	NEGATIVE
800	2014	CCHF BY PCR	KPK	Peshawar	NEGATIVE
801	2014	CCHF BY PCR	KPK	Peshawar	NEGATIVE
802	2014	CCHF BY PCR	KPK	Peshawar	NEGATIVE
803	2014	CCHF BY PCR	SINDH	Karachi	POSITIVE
804	2014	CCHF BY PCR	SINDH	Karachi	NEGATIVE
805	2014	CCHF BY PCR	KPK	Peshawar	NEGATIVE
806	2014	CCHF BY PCR	SINDH	Karachi	NEGATIVE
807	2014	CCHF BY PCR	Baluchistan	Quetta	NEGATIVE
808	2014	CCHF BY PCR	SINDH	Karachi	NEGATIVE
809	2014	CCHF BY PCR	SINDH	Karachi	NEGATIVE

810	2014	CCHF BY PCR	SINDH	Karachi	NEGATIVE
811	2014	CCHF BY PCR	SINDH	Karachi	NEGATIVE
812	2014	CCHF BY PCR	SINDH	Karachi	NEGATIVE
813	2014	CCHF BY PCR	SINDH	Karachi	NEGATIVE
814	2014	CCHF BY PCR	SINDH	Karachi	POSITIVE
815	2014	CCHF BY PCR	SINDH	Karachi	NEGATIVE
816	2014	CCHF BY PCR	SINDH	Karachi	NEGATIVE
817	2014	CCHF BY PCR	SINDH	Karachi	NEGATIVE
818	2014	CCHF BY PCR	SINDH	Karachi	POSITIVE
819	2014	CCHF BY PCR	KPK	Peshawar	NEGATIVE
820	2014	CCHF BY PCR	SINDH	Karachi	POSITIVE
821	2014	CCHF BY PCR	SINDH	Karachi	NEGATIVE
822	2014	CCHF BY PCR	KPK	Peshawar	NEGATIVE
823	2014	CCHF BY PCR	SINDH	Karachi	NEGATIVE
824	2014	CCHF BY PCR	SINDH	Karachi	NEGATIVE
825	2014	CCHF BY PCR	SINDH	Karachi	NEGATIVE
826	2015	CCHF BY PCR	SINDH	Karachi	NEGATIVE
827	2015	CCHF BY PCR	KPK	Peshawar	NEGATIVE
828	2015	CCHF BY PCR	SINDH	Karachi	NEGATIVE
829	2015	CCHF BY PCR	SINDH	Karachi	NEGATIVE
830	2015	CCHF BY PCR	SINDH	Karachi	NEGATIVE
831	2015	CCHF BY PCR	SINDH	Karachi	NEGATIVE
832	2015	CCHF BY PCR	SINDH	Karachi	NEGATIVE
833	2015	CCHF BY PCR	SINDH	Karachi	NEGATIVE
834	2015	CCHF BY PCR	PUNJAB	Rawalpindi	NEGATIVE
835	2015	CCHF BY PCR	KPK	Peshawar	NEGATIVE
836	2015	CCHF BY PCR	Baluchistan	Quetta	NEGATIVE
837	2015	CCHF BY PCR	Baluchistan	Quetta	NEGATIVE
838	2015	CCHF BY PCR	KPK	Peshawar	NEGATIVE
839	2015	CCHF BY PCR	Baluchistan	Quetta	NEGATIVE
840	2015	CCHF BY PCR	KPK	Peshawar	NEGATIVE
841	2015	CCHF BY PCR	Baluchistan	Quetta	NEGATIVE
842	2016	CCHF BY PCR	SINDH	Karachi	NEGATIVE
843	2015	CCHF BY PCR	Baluchistan	Quetta	NEGATIVE
844	2015	CCHF BY PCR	Baluchistan	Quetta	POSITIVE
845	2015	CCHF BY PCR	SINDH	Karachi	POSITIVE
846	2015	CCHF BY PCR	Baluchistan	Quetta	POSITIVE
847	2016	CCHF BY PCR	SINDH	Karachi	NEGATIVE
848	2015	CCHF BY PCR	Baluchistan	Quetta	POSITIVE
849	2015	CCHF BY PCR	KPK	Peshawar	POSITIVE

850	2016	CCHF BY PCR	SINDH	Karachi	NEGATIVE
851	2016	CCHF BY PCR	SINDH	Karachi	NEGATIVE
852	2015	CCHF BY PCR	Baluchistan	Quetta	POSITIVE
853	2015	CCHF BY PCR	SINDH	Karachi	NEGATIVE
854	2015	CCHF BY PCR	Baluchistan	Quetta	NEGATIVE
855	2015	CCHF BY PCR	Baluchistan	Quetta	POSITIVE
856	2015	CCHF BY PCR	Baluchistan	Quetta	POSITIVE
857	2015	CCHF BY PCR	KPK	Peshawar	NEGATIVE
858	2015	CCHF BY PCR	PUNJAB	MULTAN	NEGATIVE
859	2015	CCHF BY PCR	Baluchistan	Quetta	NEGATIVE
860	2015	CCHF BY PCR	KPK	Peshawar	POSITIVE
861	2015	CCHF BY PCR	SINDH	Karachi	NEGATIVE
862	2015	CCHF BY PCR	Baluchistan	Quetta	POSITIVE
863	2015	CCHF BY PCR	SINDH	Karachi	POSITIVE
864	2015	CCHF BY PCR	SINDH	Karachi	NEGATIVE
865	2015	CCHF BY PCR	Baluchistan	Quetta	POSITIVE
866	2015	CCHF BY PCR	SINDH	Karachi	NEGATIVE
867	2016	CCHF BY PCR	SINDH	Karachi	NEGATIVE
868	2015	CCHF BY PCR	Baluchistan	Quetta	POSITIVE
869	2015	CCHF BY PCR	Baluchistan	Quetta	NEGATIVE
870	2015	CCHF BY PCR	SINDH	Karachi	NEGATIVE
871	2015	CCHF BY PCR	KPK	Peshawar	NEGATIVE
872	2015	CCHF BY PCR	Baluchistan	Quetta	POSITIVE
873	2015	CCHF BY PCR	SINDH	Karachi	NEGATIVE
874	2015	CCHF BY PCR	KPK	Peshawar	POSITIVE
875	2015	CCHF BY PCR	KPK	Peshawar	POSITIVE
876	2015	CCHF BY PCR	SINDH	Karachi	POSITIVE
877	2015	CCHF BY PCR	Baluchistan	Quetta	POSITIVE
878	2015	CCHF BY PCR	KPK	Peshawar	POSITIVE
879	2015	CCHF BY PCR	Baluchistan	Quetta	NEGATIVE
880	2015	CCHF BY PCR	KPK	Peshawar	NEGATIVE
881	2015	CCHF BY PCR	SINDH	Karachi	NEGATIVE
882	2015	CCHF BY PCR	KPK	Peshawar	NEGATIVE
883	2015	CCHF BY PCR	Baluchistan	Quetta	POSITIVE
884	2015	CCHF BY PCR	Baluchistan	Quetta	POSITIVE

885	2015	CCHF BY PCR	KPK	Peshawar	POSITIVE
886	2015	CCHF BY PCR	KPK	Peshawar	NEGATIVE
887	2015	CCHF BY PCR	Baluchistan	Quetta	NEGATIVE
888	2015	CCHF BY PCR	Baluchistan	Quetta	NEGATIVE
889	2015	CCHF BY PCR	KPK	Peshawar	NEGATIVE
890	2015	CCHF BY PCR	KPK	Peshawar	NEGATIVE
891	2015	CCHF BY PCR	SINDH	Karachi	NEGATIVE
892	2015	CCHF BY PCR	Baluchistan	Quetta	NEGATIVE
893	2015	CCHF BY PCR	KPK	Peshawar	NEGATIVE
894	2015	CCHF BY PCR	KPK	Peshawar	NEGATIVE
895	2015	CCHF BY PCR	KPK	Peshawar	NEGATIVE
896	2015	CCHF BY PCR	KPK	Peshawar	POSITIVE
897	2015	CCHF BY PCR	Baluchistan	Quetta	NEGATIVE
898	2015	CCHF BY PCR	SINDH	Karachi	POSITIVE
899	2015	CCHF BY PCR	SINDH	Karachi	NEGATIVE
900	2015	CCHF BY PCR	KPK	Peshawar	NEGATIVE
901	2015	CCHF BY PCR	KPK	Peshawar	POSITIVE
902	2015	CCHF BY PCR	Baluchistan	Quetta	NEGATIVE
903	2015	CCHF BY PCR	Baluchistan	Quetta	POSITIVE
904	2019	CCHF BY PCR	KPK	Peshawar	NEGATIVE
905	2015	CCHF BY PCR	Baluchistan	Quetta	NEGATIVE
906	2015	CCHF BY PCR	SINDH	Karachi	NEGATIVE
907	2015	CCHF BY PCR	KPK	Peshawar	POSITIVE
908	2015	CCHF BY PCR	KPK	Peshawar	NEGATIVE
909	2015	CCHF BY PCR	Baluchistan	Quetta	POSITIVE
910	2015	CCHF BY PCR	Baluchistan	Quetta	NEGATIVE
911	2015	CCHF BY PCR	SINDH	Karachi	NEGATIVE
912	2015	CCHF BY PCR	SINDH	Karachi	NEGATIVE
913	2015	CCHF BY PCR	Baluchistan	Quetta	NEGATIVE
914	2015	CCHF BY PCR	Baluchistan	Quetta	NEGATIVE
915	2015	CCHF BY PCR	KPK	Abbottabad	NEGATIVE
916	2015	CCHF BY PCR	SINDH	Karachi	NEGATIVE
917	2015	CCHF BY PCR	Baluchistan	Quetta	NEGATIVE
918	2015	CCHF BY PCR	Baluchistan	Quetta	NEGATIVE
919	2015	CCHF BY PCR	KPK	Peshawar	NEGATIVE

920	2015	CCHF BY PCR	Baluchistan	Quetta	POSITIVE
921	2016	CCHF BY PCR	SINDH	Karachi	NEGATIVE
922	2015	CCHF BY PCR	SINDH	Karachi	NEGATIVE
923	2015	CCHF BY PCR	Baluchistan	Quetta	POSITIVE
924	2015	CCHF BY PCR	Baluchistan	Quetta	POSITIVE
925	2015	CCHF BY PCR	Baluchistan	Quetta	POSITIVE
926	2015	CCHF BY PCR	Baluchistan	Quetta	POSITIVE
927	2015	CCHF BY PCR	SINDH	Karachi	NEGATIVE
928	2015	CCHF BY PCR	SINDH	Karachi	NEGATIVE
929	2015	CCHF BY PCR	KPK	Peshawar	POSITIVE
930	2015	CCHF BY PCR	KPK	Peshawar	NEGATIVE
931	2016	CCHF BY PCR	SINDH	Karachi	NEGATIVE
932	2016	CCHF BY PCR	SINDH	Karachi	NEGATIVE
933	2016	CCHF BY PCR	SINDH	Karachi	NEGATIVE
934	2015	CCHF BY PCR	SINDH	Karachi	NEGATIVE
935	2015	CCHF BY PCR	Baluchistan	Quetta	POSITIVE
936	2015	CCHF BY PCR	SINDH	Karachi	NEGATIVE
937	2015	CCHF BY PCR	KPK	Peshawar	NEGATIVE
938	2015	CCHF BY PCR	Baluchistan	Quetta	NEGATIVE
939	2015	CCHF BY PCR	KPK	Peshawar	NEGATIVE
940	2015	CCHF BY PCR	SINDH	Karachi	NEGATIVE
941	2015	CCHF BY PCR	Baluchistan	Quetta	POSITIVE
942	2015	CCHF BY PCR	Baluchistan	Quetta	POSITIVE
943	2015	CCHF BY PCR	SINDH	Karachi	NEGATIVE
944	2015	CCHF BY PCR	Baluchistan	Quetta	POSITIVE
945	2015	CCHF BY PCR	SINDH	Karachi	POSITIVE
946	2015	CCHF BY PCR	SINDH	Karachi	NEGATIVE
947	2015	CCHF BY PCR	SINDH	Karachi	NEGATIVE
948	2015	CCHF BY PCR	SINDH	Karachi	NEGATIVE
949	2016	CCHF BY PCR	SINDH	Karachi	NEGATIVE
950	2015	CCHF BY PCR	KPK	Peshawar	NEGATIVE
951	2015	CCHF BY PCR	KPK	Peshawar	NEGATIVE
952	2015	CCHF BY PCR	KPK	Peshawar	NEGATIVE
953	2015	CCHF BY PCR	SINDH	Karachi	NEGATIVE
954	2015	CCHF BY PCR	SINDH	Karachi	NEGATIVE
955	2015	CCHF BY PCR	Baluchistan	Quetta	POSITIVE
956	2015	CCHF BY PCR	Baluchistan	Quetta	NEGATIVE

957	2015	CCHF BY PCR	Baluchistan	Quetta	POSITIVE
958	2015	CCHF BY PCR	Baluchistan	Quetta	POSITIVE
959	2015	CCHF BY PCR	KPK	Peshawar	NEGATIVE
960	2015	CCHF BY PCR	SINDH	Karachi	NEGATIVE
961	2015	CCHF BY PCR	PUNJAB	Rawalpindi	NEGATIVE
962	2015	CCHF BY PCR	KPK	Peshawar	NEGATIVE
963	2015	CCHF BY PCR	Baluchistan	Quetta	POSITIVE
964	2015	CCHF BY PCR	Baluchistan	Quetta	POSITIVE
965	2015	CCHF BY PCR	Baluchistan	Quetta	POSITIVE
966	2015	CCHF BY PCR	SINDH	Karachi	NEGATIVE
967	2015	CCHF BY PCR	Baluchistan	Quetta	NEGATIVE
968	2015	CCHF BY PCR	Baluchistan	Quetta	POSITIVE
969	2015	CCHF BY PCR	KPK	Peshawar	POSITIVE
970	2015	CCHF BY PCR	KPK	Peshawar	NEGATIVE
971	2015	CCHF BY PCR	Baluchistan	Quetta	NEGATIVE
972	2015	CCHF BY PCR	Baluchistan	Quetta	NEGATIVE
973	2015	CCHF BY PCR	KPK	Peshawar	NEGATIVE
974	2015	CCHF BY PCR	KPK	Peshawar	NEGATIVE
975	2015	CCHF BY PCR	KPK	Peshawar	NEGATIVE
976	2015	CCHF BY PCR	KPK	Peshawar	NEGATIVE
977	2015	CCHF BY PCR	SINDH	Karachi	NEGATIVE
978	2015	CCHF BY PCR	SINDH	Karachi	NEGATIVE
979	2015	CCHF BY PCR	SINDH	Karachi	NEGATIVE
980	2015	CCHF BY PCR	SINDH	Karachi	NEGATIVE
981	2015	CCHF BY PCR	Baluchistan	Quetta	NEGATIVE
982	2015	CCHF BY PCR	Baluchistan	Quetta	NEGATIVE
983	2015	CCHF BY PCR	Baluchistan	Quetta	NEGATIVE
984	2016	CCHF BY PCR	SINDH	Karachi	NEGATIVE
985	2015	CCHF BY PCR	KPK	Peshawar	POSITIVE
986	2015	CCHF BY PCR	Baluchistan	Quetta	NEGATIVE
987	2015	CCHF BY PCR	Baluchistan	Quetta	NEGATIVE
988	2015	CCHF BY PCR	KPK	Peshawar	NEGATIVE
989	2015	CCHF BY PCR	Baluchistan	Quetta	NEGATIVE
990	2015	CCHF BY PCR	Baluchistan	Quetta	NEGATIVE

991	2015	CCHF BY PCR	Baluchistan	Quetta	NEGATIVE
992	2015	CCHF BY PCR	Baluchistan	Quetta	NEGATIVE
993	2015	CCHF BY PCR	Baluchistan	Quetta	NEGATIVE
994	2015	CCHF BY PCR	Baluchistan	Quetta	NEGATIVE
995	2015	CCHF BY PCR	SINDH	Karachi	NEGATIVE
996	2015	CCHF BY PCR	Baluchistan	Quetta	NEGATIVE
997	2015	CCHF BY PCR	Baluchistan	Quetta	NEGATIVE
998	2017	CCHF BY PCR	SINDH	Karachi	NEGATIVE
999	2015	CCHF BY PCR	KPK	Peshawar	NEGATIVE
1000	2015	CCHF BY PCR	SINDH	Karachi	NEGATIVE
1001	2015	CCHF BY PCR	SINDH	Karachi	NEGATIVE
1002	2016	CCHF BY PCR	SINDH	Karachi	NEGATIVE
1003	2015	CCHF BY PCR	Baluchistan	Quetta	POSITIVE
1004	2015	CCHF BY PCR	Baluchistan	Quetta	NEGATIVE
1005	2015	CCHF BY PCR	KPK	Peshawar	NEGATIVE
1006	2015	CCHF BY PCR	KPK	Peshawar	NEGATIVE
1007	2015	CCHF BY PCR	Baluchistan	Quetta	NEGATIVE
1008	2015	CCHF BY PCR	KPK	Peshawar	NEGATIVE
1009	2015	CCHF BY PCR	Baluchistan	Quetta	NEGATIVE
1010	2015	CCHF BY PCR	Baluchistan	Quetta	NEGATIVE
1011	2015	CCHF BY PCR	Baluchistan	Quetta	NEGATIVE
1012	2015	CCHF BY PCR	SINDH	Karachi	NEGATIVE
1013	2015	CCHF BY PCR	KPK	Peshawar	NEGATIVE
1014	2015	CCHF BY PCR	PUNJAB	MULTAN	NEGATIVE
1015	2015	CCHF BY PCR	SINDH	Karachi	NEGATIVE
1016	2015	CCHF BY PCR	SINDH	Karachi	NEGATIVE
1017	2015	CCHF BY PCR	Baluchistan	Quetta	NEGATIVE
1018	2016	CCHF BY PCR	KPK	Peshawar	NEGATIVE
1019	2016	CCHF BY PCR	Baluchistan	Quetta	NEGATIVE
1020	2016	CCHF BY PCR	Baluchistan	Quetta	NEGATIVE
1021	2016	CCHF BY PCR	KPK	Peshawar	POSITIVE
1022	2016	CCHF BY PCR	KPK	Peshawar	NEGATIVE
1023	2016	CCHF BY PCR	SINDH	Karachi	NEGATIVE
1024	2016	CCHF BY PCR	KPK	Peshawar	NEGATIVE

1025	2016	CCHF BY PCR	SINDH	Karachi	NEGATIVE
1026	2016	CCHF BY PCR	Baluchistan	Quetta	NEGATIVE
1027	2016	CCHF BY PCR	PUNJAB	Rawalpindi	NEGATIVE
1028	2016	CCHF BY PCR	Baluchistan	Quetta	POSITIVE
1029	2019	CCHF BY PCR	PUNJAB	RAHIM YAR KHAN	NEGATIVE
1030	2016	CCHF BY PCR	Baluchistan	Quetta	NEGATIVE
1031	2016	CCHF BY PCR	SINDH	Karachi	NEGATIVE
1032	2016	CCHF BY PCR	SINDH	Karachi	NEGATIVE
1033	2016	CCHF BY PCR	Baluchistan	Quetta	NEGATIVE
1034	2016	CCHF BY PCR	Baluchistan	Quetta	NEGATIVE
1035	2016	CCHF BY PCR	Baluchistan	Quetta	NEGATIVE
1036	2016	CCHF BY PCR	PUNJAB	Bahawalpur	NEGATIVE
1037	2016	CCHF BY PCR	SINDH	Karachi	NEGATIVE
1038	2016	CCHF BY PCR	PUNJAB	Islamabad	NEGATIVE
1039	2016	CCHF BY PCR	SINDH	Karachi	NEGATIVE
1040	2016	CCHF BY PCR	Baluchistan	Quetta	POSITIVE
1041	2016	CCHF BY PCR	SINDH	Karachi	NEGATIVE
1042	2016	CCHF BY PCR	SINDH	Karachi	NEGATIVE
1043	2016	CCHF BY PCR	Baluchistan	Quetta	POSITIVE
1044	2016	CCHF BY PCR	Baluchistan	Quetta	NEGATIVE
1045	2016	CCHF BY PCR	Baluchistan	Quetta	NEGATIVE
1046	2016	CCHF BY PCR	SINDH	Karachi	NEGATIVE
1047	2016	CCHF BY PCR	Baluchistan	Quetta	NEGATIVE
1048	2016	CCHF BY PCR	SINDH	Karachi	NEGATIVE
1049	2016	CCHF BY PCR	KPK	Peshawar	NEGATIVE
1050	2016	CCHF BY PCR	Baluchistan	Quetta	NEGATIVE
1051	2016	CCHF BY PCR	KPK	Peshawar	NEGATIVE
1052	2016	CCHF BY PCR	KPK	Peshawar	NEGATIVE
1053	2016	CCHF BY PCR	Baluchistan	Quetta	NEGATIVE
1054	2016	CCHF BY PCR	Baluchistan	Quetta	NEGATIVE
1055	2016	CCHF BY PCR	Baluchistan	Quetta	NEGATIVE
1056	2016	CCHF BY PCR	Baluchistan	Quetta	POSITIVE

1057	2016	CCHF BY PCR	Baluchistan	Quetta	POSITIVE
1058	2016	CCHF BY PCR	Baluchistan	Quetta	POSITIVE
1059	2016	CCHF BY PCR	Baluchistan	Quetta	NEGATIVE
1060	2016	CCHF BY PCR	KPK	Peshawar	NEGATIVE
1061	2016	CCHF BY PCR	Baluchistan	Quetta	NEGATIVE
1062	2016	CCHF BY PCR	Baluchistan	Quetta	NEGATIVE
1063	2016	CCHF BY PCR	SINDH	Karachi	NEGATIVE
1064	2016	CCHF BY PCR	Baluchistan	Quetta	POSITIVE
1065	2016	CCHF BY PCR	Baluchistan	Quetta	POSITIVE
1066	2016	CCHF BY PCR	Baluchistan	Quetta	NEGATIVE
1067	2016	CCHF BY PCR	Baluchistan	Quetta	NEGATIVE
1068	2016	CCHF BY PCR	Baluchistan	Quetta	NEGATIVE
1069	2016	CCHF BY PCR	Baluchistan	Quetta	NEGATIVE
1070	2016	CCHF BY PCR	Baluchistan	Quetta	NEGATIVE
1071	2016	CCHF BY PCR	Baluchistan	Quetta	POSITIVE
1072	2016	CCHF BY PCR	Baluchistan	Quetta	NEGATIVE
1073	2016	CCHF BY PCR	Baluchistan	Quetta	POSITIVE
1074	2016	CCHF BY PCR	Baluchistan	Quetta	POSITIVE
1075	2016	CCHF BY PCR	Baluchistan	Quetta	POSITIVE
1076	2016	CCHF BY PCR	KPK	Peshawar	NEGATIVE
1077	2016	CCHF BY PCR	Baluchistan	Quetta	NEGATIVE
1078	2016	CCHF BY PCR	Baluchistan	Quetta	NEGATIVE
1079	2016	CCHF BY PCR	Baluchistan	Quetta	NEGATIVE
1080	2016	CCHF BY PCR	Baluchistan	Quetta	NEGATIVE
1081	2016	CCHF BY PCR	Baluchistan	Quetta	POSITIVE
1082	2016	CCHF BY PCR	Baluchistan	Quetta	POSITIVE
1083	2016	CCHF BY PCR	Baluchistan	Quetta	NEGATIVE

1084	2016	CCHF BY PCR	Baluchistan	Quetta	NEGATIVE
1085	2016	CCHF BY PCR	Baluchistan	Quetta	NEGATIVE
1086	2016	CCHF BY PCR	Baluchistan	Quetta	NEGATIVE
1087	2016	CCHF BY PCR	Baluchistan	Quetta	NEGATIVE
1088	2016	CCHF BY PCR	Baluchistan	Quetta	POSITIVE
1089	2016	CCHF BY PCR	Baluchistan	Quetta	NEGATIVE
1090	2016	CCHF BY PCR	Baluchistan	Quetta	POSITIVE
1091	2016	CCHF BY PCR	Baluchistan	Quetta	NEGATIVE
1092	2016	CCHF BY PCR	Baluchistan	Quetta	NEGATIVE
1093	2016	CCHF BY PCR	Baluchistan	Quetta	NEGATIVE
1094	2016	CCHF BY PCR	Baluchistan	Quetta	NEGATIVE
1095	2016	CCHF BY PCR	SINDH	Karachi	NEGATIVE
1096	2016	CCHF BY PCR	KPK	Peshawar	POSITIVE
1097	2016	CCHF BY PCR	SINDH	Hyderabad	NEGATIVE
1098	2016	CCHF BY PCR	KPK	Peshawar	POSITIVE
1099	2016	CCHF BY PCR	Baluchistan	Quetta	POSITIVE
1100	2016	CCHF BY PCR	Baluchistan	Quetta	POSITIVE
1101	2016	CCHF BY PCR	PUNJAB	Bahawalpur	NEGATIVE
1102	2016	CCHF BY PCR	PUNJAB	Rawalpindi	NEGATIVE
1103	2016	CCHF BY PCR	PUNJAB	Bahawalpur	NEGATIVE
1104	2016	CCHF BY PCR	PUNJAB	Islamabad	NEGATIVE
1105	2016	CCHF BY PCR	SINDH	Karachi	NEGATIVE
1106	2016	CCHF BY PCR	SINDH	Karachi	NEGATIVE
1107	2016	CCHF BY PCR	SINDH	Karachi	NEGATIVE
1108	2016	CCHF BY PCR	SINDH	Karachi	NEGATIVE
1109	2016	CCHF BY PCR	PUNJAB	Bahawalpur	NEGATIVE
1110	2016	CCHF BY PCR	SINDH	Karachi	NEGATIVE
1111	2016	CCHF BY PCR	PUNJAB	Bahawalpur	NEGATIVE
1112	2016	CCHF BY PCR	PUNJAB	Bahawalpur	NEGATIVE
1113	2016	CCHF BY PCR	PUNJAB	Bahawalpur	NEGATIVE
1114	2016	CCHF BY PCR	PUNJAB	Bahawalpur	NEGATIVE
1115	2016	CCHF BY PCR	PUNJAB	Bahawalpur	NEGATIVE
1116	2016	CCHF BY PCR	PUNJAB	MULTAN	NEGATIVE
1117	2016	CCHF BY PCR	PUNJAB	Bahawalpur	NEGATIVE
1118	2016	CCHF BY PCR	SINDH	Karachi	NEGATIVE

1119	2016	CCHF BY PCR	Baluchistan	Quetta	POSITIVE
1120	2016	CCHF BY PCR	Baluchistan	Quetta	NEGATIVE
1121	2016	CCHF BY PCR	Baluchistan	Quetta	NEGATIVE
1122	2016	CCHF BY PCR	Baluchistan	Quetta	POSITIVE
1123	2016	CCHF BY PCR	SINDH	Karachi	NEGATIVE
1124	2016	CCHF BY PCR	SINDH	Karachi	NEGATIVE
1125	2016	CCHF BY PCR	SINDH	Karachi	NEGATIVE
1126	2016	CCHF BY PCR	SINDH	Karachi	NEGATIVE
1127	2016	CCHF BY PCR	Baluchistan	Quetta	NEGATIVE
1128	2016	CCHF BY PCR	Baluchistan	Quetta	POSITIVE
1129	2016	CCHF BY PCR	PUNJAB	RAHIM YAR KHAN	NEGATIVE
1130	2016	CCHF BY PCR	SINDH	Karachi	NEGATIVE
1131	2016	CCHF BY PCR	KPK	Abbottabad	NEGATIVE
1132	2016	CCHF BY PCR	KPK	Peshawar	NEGATIVE
1133	2016	CCHF BY PCR	Baluchistan	Quetta	NEGATIVE
1134	2016	CCHF BY PCR	PUNJAB	Rawalpindi	NEGATIVE
1135	2016	CCHF BY PCR	Baluchistan	Quetta	POSITIVE
1136	2016	CCHF BY PCR	Baluchistan	Quetta	POSITIVE
1137	2016	CCHF BY PCR	PUNJAB	DERA GHAZI KHAN	NEGATIVE
1138	2016	CCHF BY PCR	SINDH	Karachi	NEGATIVE
1139	2016	CCHF BY PCR	SINDH	Karachi	NEGATIVE
1140	2016	CCHF BY PCR	PUNJAB	RAHIM YAR KHAN	NEGATIVE
1141	2016	CCHF BY PCR	Baluchistan	Quetta	POSITIVE
1142	2016	CCHF BY PCR	PUNJAB	Bahawalpur	NEGATIVE
1143	2016	CCHF BY PCR	PUNJAB	RAHIM YAR KHAN	NEGATIVE
1144	2016	CCHF BY PCR	KPK	Peshawar	NEGATIVE
1145	2016	CCHF BY PCR	Baluchistan	Quetta	POSITIVE
1146	2016	CCHF BY PCR	SINDH	Karachi	POSITIVE
1147	2016	CCHF BY PCR	SINDH	Karachi	POSITIVE
1148	2016	CCHF BY PCR	Baluchistan	Quetta	NEGATIVE
1149	2016	CCHF BY PCR	PUNJAB	Lahore	NEGATIVE
1150	2016	CCHF BY PCR	PUNJAB	Lahore	NEGATIVE
1151	2016	CCHF BY PCR	SINDH	Karachi	NEGATIVE

1152	2016	CCHF BY PCR	Baluchistan	Quetta	NEGATIVE
1153	2016	CCHF BY PCR	SINDH	Karachi	NEGATIVE
1154	2016	CCHF BY PCR	KPK	Peshawar	POSITIVE
1155	2016	CCHF BY PCR	Baluchistan	Quetta	POSITIVE
1156	2016	CCHF BY PCR	Baluchistan	Quetta	POSITIVE
1157	2016	CCHF BY PCR	SINDH	Karachi	NEGATIVE
1158	2016	CCHF BY PCR	PUNJAB	RAHIM YAR KHAN	NEGATIVE
1159	2016	CCHF BY PCR	Baluchistan	Quetta	NEGATIVE
1160	2016	CCHF BY PCR	Baluchistan	Quetta	POSITIVE
1161	2016	CCHF BY PCR	KPK	Peshawar	NEGATIVE
1162	2016	CCHF BY PCR	SINDH	Sukkur	NEGATIVE
1163	2016	CCHF BY PCR	SINDH	Nawabshah	NEGATIVE
1164	2016	CCHF BY PCR	SINDH	Karachi	NEGATIVE
1165	2016	CCHF BY PCR	Baluchistan	Quetta	NEGATIVE
1166	2016	CCHF BY PCR	SINDH	Karachi	NEGATIVE
1167	2016	CCHF BY PCR	KPK	Abbottabad	NEGATIVE
1168	2016	CCHF BY PCR	PUNJAB	Lahore	NEGATIVE
1169	2016	CCHF BY PCR	SINDH	Karachi	NEGATIVE
1170	2016	CCHF BY PCR	Baluchistan	Quetta	POSITIVE
1171	2016	CCHF BY PCR	Baluchistan	Quetta	NEGATIVE
1172	2016	CCHF BY PCR	SINDH	Karachi	NEGATIVE
1173	2016	CCHF BY PCR	SINDH	Karachi	NEGATIVE
1174	2016	CCHF BY PCR	Baluchistan	Quetta	POSITIVE
1175	2016	CCHF BY PCR	SINDH	Karachi	NEGATIVE
1176	2016	CCHF BY PCR	KPK	Peshawar	NEGATIVE
1177	2016	CCHF BY PCR	SINDH	Karachi	NEGATIVE
1178	2016	CCHF BY PCR	SINDH	Karachi	NEGATIVE
1179	2016	CCHF BY PCR	SINDH	Karachi	NEGATIVE
1180	2016	CCHF BY PCR	SINDH	Karachi	POSITIVE
1181	2016	CCHF BY PCR	KPK	Peshawar	NEGATIVE
1182	2016	CCHF BY PCR	Baluchistan	Quetta	POSITIVE
1183	2016	CCHF BY PCR	KPK	Peshawar	NEGATIVE
1184	2016	CCHF BY PCR	Baluchistan	Quetta	NEGATIVE
1185	2016	CCHF BY PCR	Baluchistan	Quetta	NEGATIVE
1186	2016	CCHF BY PCR	SINDH	Karachi	NEGATIVE
1187	2016	CCHF BY PCR	SINDH	Karachi	NEGATIVE

1188	2016	CCHF BY PCR	PUNJAB	DERA GHAZI KHAN	NEGATIVE
1189	2016	CCHF BY PCR	KPK	Peshawar	NEGATIVE
1190	2016	CCHF BY PCR	SINDH	Karachi	NEGATIVE
1191	2016	CCHF BY PCR	SINDH	Karachi	NEGATIVE
1192	2016	CCHF BY PCR	Baluchistan	Quetta	POSITIVE
1193	2016	CCHF BY PCR	SINDH	Karachi	NEGATIVE
1194	2016	CCHF BY PCR	SINDH	Karachi	NEGATIVE
1195	2016	CCHF BY PCR	Baluchistan	Quetta	POSITIVE
1196	2016	CCHF BY PCR	SINDH	Karachi	NEGATIVE
1197	2016	CCHF BY PCR	PUNJAB	Lahore	NEGATIVE
1198	2016	CCHF BY PCR	SINDH	Karachi	NEGATIVE
1199	2016	CCHF BY PCR	SINDH	Sanghar	NEGATIVE
1200	2016	CCHF BY PCR	PUNJAB	JAMPUR POINT	NEGATIVE
1201	2016	CCHF BY PCR	Baluchistan	Quetta	NEGATIVE
1202	2016	CCHF BY PCR	Baluchistan	Quetta	NEGATIVE
1203	2016	CCHF BY PCR	Baluchistan	Quetta	POSITIVE
1204	2016	CCHF BY PCR	SINDH	Karachi	NEGATIVE
1205	2016	CCHF BY PCR	SINDH	Karachi	NEGATIVE
1206	2016	CCHF BY PCR	SINDH	Karachi	NEGATIVE
1207	2016	CCHF BY PCR	SINDH	Karachi	NEGATIVE
1208	2016	CCHF BY PCR	SINDH	Karachi	NEGATIVE
1209	2016	CCHF BY PCR	SINDH	Karachi	NEGATIVE
1210	2016	CCHF BY PCR	SINDH	Karachi	NEGATIVE
1211	2016	CCHF BY PCR	SINDH	Karachi	NEGATIVE
1212	2016	CCHF BY PCR	SINDH	Karachi	NEGATIVE
1213	2016	CCHF BY PCR	SINDH	Karachi	NEGATIVE
1214	2016	CCHF BY PCR	SINDH	Karachi	NEGATIVE
1215	2016	CCHF BY PCR	SINDH	Karachi	NEGATIVE
1216	2016	CCHF BY PCR	Baluchistan	Quetta	NEGATIVE
1217	2016	CCHF BY PCR	SINDH	Karachi	NEGATIVE
1218	2016	CCHF BY PCR	Baluchistan	Quetta	POSITIVE
1219	2016	CCHF BY PCR	KPK	Peshawar	NEGATIVE
1220	2016	CCHF BY PCR	SINDH	Karachi	NEGATIVE
1221	2016	CCHF BY PCR	KPK	Peshawar	NEGATIVE
1222	2016	CCHF BY PCR	SINDH	Karachi	NEGATIVE
1223	2016	CCHF BY PCR	PUNJAB	Lahore	NEGATIVE
1224	2016	CCHF BY PCR	SINDH	Karachi	NEGATIVE
1225	2016	CCHF BY PCR	PUNJAB	Khanpur	NEGATIVE
1226	2016	CCHF BY PCR	PUNJAB	Lahore	NEGATIVE
1227	2016	CCHF BY PCR	PUNJAB	Islamabad	NEGATIVE

1228	2016	CCHF BY PCR	PUNJAB	Lahore	NEGATIVE
1229	2016	CCHF BY PCR	PUNJAB	Bahawalpur	NEGATIVE
1230	2016	CCHF BY PCR	SINDH	Karachi	NEGATIVE
1231	2016	CCHF BY PCR	KPK	Peshawar	POSITIVE
1232	2016	CCHF BY PCR	PUNJAB	Lahore	NEGATIVE
1233	2016	CCHF BY PCR	SINDH	Karachi	NEGATIVE
1234	2016	CCHF BY PCR	Baluchistan	Quetta	NEGATIVE
1235	2016	CCHF BY PCR	Baluchistan	Quetta	NEGATIVE
1236	2016	CCHF BY PCR	PUNJAB	Lahore	NEGATIVE
1237	2016	CCHF BY PCR	SINDH	Karachi	NEGATIVE
1238	2016	CCHF BY PCR	SINDH	Karachi	NEGATIVE
1239	2016	CCHF BY PCR	SINDH	Karachi	POSITIVE
1240	2016	CCHF BY PCR	PUNJAB	Rawalpindi	NEGATIVE
1241	2016	CCHF BY PCR	PUNJAB	Rawalpindi	NEGATIVE
1242	2016	CCHF BY PCR	PUNJAB	Rawalpindi	NEGATIVE
1243	2016	CCHF BY PCR	SINDH	Karachi	NEGATIVE
1244	2016	CCHF BY PCR	KPK	Peshawar	NEGATIVE
1245	2016	CCHF BY PCR	SINDH	Karachi	NEGATIVE
1246	2016	CCHF BY PCR	Baluchistan	Quetta	POSITIVE
1247	2016	CCHF BY PCR	Baluchistan	Quetta	NEGATIVE
1248	2016	CCHF BY PCR	Baluchistan	Quetta	NEGATIVE
1249	2016	CCHF BY PCR	Baluchistan	Quetta	NEGATIVE
1250	2016	CCHF BY PCR	KPK	Peshawar	NEGATIVE
1251	2016	CCHF BY PCR	SINDH	Karachi	NEGATIVE
1252	2016	CCHF BY PCR	PUNJAB	Islamabad	NEGATIVE
1253	2016	CCHF BY PCR	Baluchistan	Quetta	POSITIVE
1254	2016	CCHF BY PCR	SINDH	Karachi	NEGATIVE
1255	2016	CCHF BY PCR	SINDH	Karachi	NEGATIVE
1256	2016	CCHF BY PCR	KPK	Peshawar	POSITIVE
1257	2016	CCHF BY PCR	SINDH	Karachi	POSITIVE
1258	2016	CCHF BY PCR	Baluchistan	Quetta	NEGATIVE
1259	2016	CCHF BY PCR	KPK	Peshawar	NEGATIVE
1260	2016	CCHF BY PCR	PUNJAB	MULTAN	NEGATIVE
1261	2016	CCHF BY PCR	SINDH	Karachi	POSITIVE
1262	2016	CCHF BY PCR	Baluchistan	Quetta	POSITIVE
1263	2016	CCHF BY PCR	Baluchistan	Quetta	POSITIVE
1264	2016	CCHF BY PCR	Baluchistan	Quetta	NEGATIVE
1265	2016	CCHF BY PCR	KPK	Peshawar	NEGATIVE

1266	2016	CCHF BY PCR	KPK	Peshawar	NEGATIVE
1267	2016	CCHF BY PCR	SINDH	Karachi	POSITIVE
1268	2016	CCHF BY PCR	SINDH	Hyderabad	POSITIVE
1269	2016	CCHF BY PCR	KPK	Peshawar	NEGATIVE
1270	2016	CCHF BY PCR	Baluchistan	Quetta	POSITIVE
1271	2016	CCHF BY PCR	Baluchistan	Quetta	POSITIVE
1272	2016	CCHF BY PCR	Baluchistan	Quetta	POSITIVE
1273	2021	CCHF BY PCR	SINDH	Karachi	NEGATIVE
1274	2016	CCHF BY PCR	KPK	Peshawar	NEGATIVE
1275	2016	CCHF BY PCR	KPK	Dera Ismail Khan	NEGATIVE
1276	2016	CCHF BY PCR	KPK	Dera Ismail Khan	NEGATIVE
1277	2016	CCHF BY PCR	Baluchistan	Quetta	POSITIVE
1278	2016	CCHF BY PCR	Baluchistan	Quetta	POSITIVE
1279	2016	CCHF BY PCR	Baluchistan	Quetta	POSITIVE
1280	2016	CCHF BY PCR	KPK	Peshawar	NEGATIVE
1281	2016	CCHF BY PCR	Baluchistan	Quetta	POSITIVE
1282	2016	CCHF BY PCR	Baluchistan	Quetta	POSITIVE
1283	2016	CCHF BY PCR	Baluchistan	Quetta	NEGATIVE
1284	2016	CCHF BY PCR	KPK	Peshawar	NEGATIVE
1285	2016	CCHF BY PCR	SINDH	Karachi	NEGATIVE
1286	2016	CCHF BY PCR	SINDH	Karachi	NEGATIVE
1287	2016	CCHF BY PCR	SINDH	Karachi	NEGATIVE
1288	2016	CCHF BY PCR	Baluchistan	Quetta	NEGATIVE
1289	2016	CCHF BY PCR	KPK	Peshawar	NEGATIVE
1290	2016	CCHF BY PCR	Baluchistan	Quetta	POSITIVE
1291	2016	CCHF BY PCR	SINDH	Karachi	NEGATIVE
1292	2016	CCHF BY PCR	SINDH	Karachi	POSITIVE
1293	2016	CCHF BY PCR	SINDH	Hyderabad	NEGATIVE
1294	2016	CCHF BY PCR	SINDH	Karachi	NEGATIVE
1295	2016	CCHF BY PCR	SINDH	Karachi	NEGATIVE
1296	2016	CCHF BY PCR	KPK	Peshawar	POSITIVE
1297	2016	CCHF BY PCR	Baluchistan	Quetta	POSITIVE
1298	2016	CCHF BY PCR	Baluchistan	Quetta	NEGATIVE
1299	2016	CCHF BY PCR	PUNJAB	Islamabad	POSITIVE
1300	2016	CCHF BY PCR	Baluchistan	Quetta	NEGATIVE

1301	2016	CCHF BY PCR	KPK	Peshawar	NEGATIVE
1302	2016	CCHF BY PCR	SINDH	Karachi	NEGATIVE
1303	2016	CCHF BY PCR	PUNJAB	Faisalabad	NEGATIVE
1304	2016	CCHF BY PCR	KPK	Peshawar	NEGATIVE
1305	2016	CCHF BY PCR	Baluchistan	Quetta	POSITIVE
1306	2016	CCHF BY PCR	Baluchistan	Quetta	NEGATIVE
1307	2016	CCHF BY PCR	Baluchistan	Quetta	POSITIVE
1308	2016	CCHF BY PCR	SINDH	Karachi	NEGATIVE
1309	2016	CCHF BY PCR	SINDH	Karachi	NEGATIVE
1310	2016	CCHF BY PCR	SINDH	Karachi	NEGATIVE
1311	2016	CCHF BY PCR	SINDH	Karachi	NEGATIVE
1312	2016	CCHF BY PCR	Baluchistan	Quetta	POSITIVE
1313	2016	CCHF BY PCR	Baluchistan	Quetta	POSITIVE
1314	2016	CCHF BY PCR	SINDH	Hyderabad	POSITIVE
1315	2016	CCHF BY PCR	SINDH	Karachi	NEGATIVE
1316	2016	CCHF BY PCR	SINDH	Karachi	NEGATIVE
1317	2016	CCHF BY PCR	SINDH	Karachi	NEGATIVE
1318	2016	CCHF BY PCR	PUNJAB	MULTAN	NEGATIVE
1319	2016	CCHF BY PCR	Baluchistan	Quetta	NEGATIVE
1320	2016	CCHF BY PCR	Baluchistan	Quetta	NEGATIVE
1321	2016	CCHF BY PCR	SINDH	Karachi	NEGATIVE
1322	2016	CCHF BY PCR	SINDH	Karachi	NEGATIVE
1323	2016	CCHF BY PCR	SINDH	Karachi	NEGATIVE
1324	2016	CCHF BY PCR	SINDH	Karachi	NEGATIVE
1325	2016	CCHF BY PCR	SINDH	Karachi	NEGATIVE
1326	2016	CCHF BY PCR	SINDH	Karachi	NEGATIVE
1327	2016	CCHF BY PCR	Baluchistan	Quetta	POSITIVE
1328	2016	CCHF BY PCR	Baluchistan	Quetta	NEGATIVE
1329	2016	CCHF BY PCR	SINDH	Hyderabad	NEGATIVE
1330	2016	CCHF BY PCR	SINDH	Hyderabad	NEGATIVE
1331	2016	CCHF BY PCR	Baluchistan	Quetta	POSITIVE
1332	2016	CCHF BY PCR	SINDH	Karachi	NEGATIVE
1333	2016	CCHF BY PCR	SINDH	DHARKI	NEGATIVE
1334	2016	CCHF BY PCR	Baluchistan	Loralai	NEGATIVE
1335	2016	CCHF BY PCR	KPK	Peshawar	NEGATIVE
1336	2016	CCHF BY PCR	SINDH	Karachi	NEGATIVE
1337	2016	CCHF BY PCR	PUNJAB	Lahore	NEGATIVE
1338	2016	CCHF BY PCR	SINDH	Karachi	POSITIVE

1339	2016	CCHF BY PCR	SINDH	Karachi	NEGATIVE
1340	2016	CCHF BY PCR	SINDH	Karachi	NEGATIVE
1341	2016	CCHF BY PCR	SINDH	Karachi	NEGATIVE
1342	2016	CCHF BY PCR	SINDH	Karachi	NEGATIVE
1343	2016	CCHF BY PCR	SINDH	Karachi	NEGATIVE
1344	2016	CCHF BY PCR	Baluchistan	Quetta	POSITIVE
1345	2016	CCHF BY PCR	Baluchistan	Quetta	NEGATIVE
1346	2016	CCHF BY PCR	SINDH	Karachi	NEGATIVE
1347	2016	CCHF BY PCR	KPK	Peshawar	POSITIVE
1348	2016	CCHF BY PCR	SINDH	Nawabshah	NEGATIVE
1349	2016	CCHF BY PCR	Baluchistan	Quetta	NEGATIVE
1350	2016	CCHF BY PCR	SINDH	Karachi	NEGATIVE
1351	2016	CCHF BY PCR	KPK	Abbottabad	NEGATIVE
1352	2016	CCHF BY PCR	Baluchistan	Quetta	NEGATIVE
1353	2016	CCHF BY PCR	SINDH	Karachi	NEGATIVE
1354	2016	CCHF BY PCR	SINDH	Karachi	NEGATIVE
1355	2016	CCHF BY PCR	KPK	Peshawar	NEGATIVE
1356	2016	CCHF BY PCR	SINDH	Karachi	NEGATIVE
1357	2016	CCHF BY PCR	Baluchistan	Quetta	NEGATIVE
1358	2016	CCHF BY PCR	KPK	Peshawar	NEGATIVE
1359	2016	CCHF BY PCR	SINDH	Karachi	NEGATIVE
1360	2016	CCHF BY PCR	SINDH	Karachi	NEGATIVE
1361	2016	CCHF BY PCR	SINDH	Karachi	NEGATIVE
1362	2016	CCHF BY PCR	KPK	Peshawar	NEGATIVE
1363	2016	CCHF BY PCR	SINDH	Karachi	NEGATIVE
1364	2017	CCHF BY PCR	PUNJAB	Bahawalpur	NEGATIVE
1365	2016	CCHF BY PCR	SINDH	Karachi	NEGATIVE
1366	2016	CCHF BY PCR	KPK	Peshawar	NEGATIVE
1367	2016	CCHF BY PCR	SINDH	Karachi	NEGATIVE
1368	2016	CCHF BY PCR	SINDH	Karachi	NEGATIVE
1369	2016	CCHF BY PCR	SINDH	Hyderabad	NEGATIVE
1370	2016	CCHF BY PCR	SINDH	Karachi	NEGATIVE
1371	2016	CCHF BY PCR	PUNJAB	Islamabad	NEGATIVE
1372	2016	CCHF BY PCR	SINDH	Karachi	NEGATIVE
1373	2016	CCHF BY PCR	KPK	Peshawar	NEGATIVE
1374	2016	CCHF BY PCR	SINDH	Karachi	NEGATIVE
1375	2016	CCHF BY PCR	SINDH	Karachi	NEGATIVE
1376	2016	CCHF BY PCR	SINDH	Karachi	NEGATIVE
1377	2016	CCHF BY PCR	SINDH	Karachi	NEGATIVE
1378	2016	CCHF BY PCR	SINDH	Karachi	NEGATIVE
1379	2016	CCHF BY PCR	KPK	Peshawar	NEGATIVE
1380	2016	CCHF BY PCR	SINDH	Karachi	NEGATIVE

1381	2016	CCHF BY PCR	SINDH	Karachi	NEGATIVE
1382	2017	CCHF BY PCR	SINDH	Karachi	NEGATIVE
1383	2017	CCHF BY PCR	PUNJAB	Faisalabad	NEGATIVE
1384	2017	CCHF BY PCR	PUNJAB	Lahore	NEGATIVE
1385	2017	CCHF BY PCR	KPK	Mardan	NEGATIVE
1386	2017	CCHF BY PCR	KPK	Mardan	NEGATIVE
1387	2017	CCHF BY PCR	KPK	Mardan	NEGATIVE
1388	2017	CCHF BY PCR	KPK	Mardan	NEGATIVE
1389	2017	CCHF BY PCR	KPK	Mardan	NEGATIVE
1390	2017	CCHF BY PCR	PUNJAB	Islamabad	NEGATIVE
1391	2017	CCHF BY PCR	Baluchistan	Quetta	NEGATIVE
1392	2017	CCHF BY PCR	KPK	Peshawar	NEGATIVE
1393	2017	CCHF BY PCR	Baluchistan	Quetta	NEGATIVE
1394	2017	CCHF BY PCR	A.Z.J.K	Mirpur	NEGATIVE
1395	2017	CCHF BY PCR	KPK	Peshawar	NEGATIVE
1396	2017	CCHF BY PCR	Baluchistan	Quetta	NEGATIVE
1397	2017	CCHF BY PCR	Baluchistan	Quetta	NEGATIVE
1398	2017	CCHF BY PCR	SINDH	Karachi	NEGATIVE
1399	2017	CCHF BY PCR	SINDH	Karachi	NEGATIVE
1400	2017	CCHF BY PCR	Baluchistan	Quetta	POSITIVE
1401	2017	CCHF BY PCR	SINDH	Karachi	NEGATIVE
1402	2017	CCHF BY PCR	Baluchistan	Quetta	POSITIVE
1403	2017	CCHF BY PCR	Baluchistan	Quetta	NEGATIVE
1404	2017	CCHF BY PCR	SINDH	Karachi	POSITIVE
1405	2017	CCHF BY PCR	Baluchistan	Quetta	POSITIVE
1406	2017	CCHF BY PCR	SINDH	Karachi	NEGATIVE
1407	2017	CCHF BY PCR	PUNJAB	Lahore	POSITIVE
1408	2017	CCHF BY PCR	Baluchistan	Quetta	POSITIVE
1409	2017	CCHF BY PCR	Baluchistan	Quetta	POSITIVE
1410	2017	CCHF BY PCR	Baluchistan	Quetta	NEGATIVE
1411	2017	CCHF BY PCR	PUNJAB	MULTAN	NEGATIVE
1412	2017	CCHF BY PCR	SINDH	Karachi	POSITIVE
1413	2018	CCHF BY PCR	SINDH	Hyderabad	NEGATIVE
1414	2017	CCHF BY PCR	Baluchistan	Quetta	NEGATIVE
1415	2017	CCHF BY PCR	Baluchistan	Quetta	NEGATIVE

1416	2017	CCHF BY PCR	Baluchistan	Quetta	NEGATIVE
1417	2017	CCHF BY PCR	Baluchistan	Quetta	NEGATIVE
1418	2017	CCHF BY PCR	Baluchistan	Quetta	NEGATIVE
1419	2017	CCHF BY PCR	SINDH	Karachi	NEGATIVE
1420	2017	CCHF BY PCR	Baluchistan	Quetta	NEGATIVE
1421	2017	CCHF BY PCR	SINDH	Karachi	NEGATIVE
1422	2017	CCHF BY PCR	SINDH	Karachi	NEGATIVE
1423	2019	CCHF BY PCR	SINDH	Karachi	NEGATIVE
1424	2017	CCHF BY PCR	KPK	Peshawar	NEGATIVE
1425	2017	CCHF BY PCR	Baluchistan	Quetta	POSITIVE
1426	2017	CCHF BY PCR	Baluchistan	Quetta	NEGATIVE
1427	2017	CCHF BY PCR	SINDH	Karachi	NEGATIVE
1428	2017	CCHF BY PCR	SINDH	Karachi	NEGATIVE
1429	2017	CCHF BY PCR	SINDH	Karachi	NEGATIVE
1430	2017	CCHF BY PCR	Baluchistan	Quetta	NEGATIVE
1431	2017	CCHF BY PCR	Baluchistan	Quetta	NEGATIVE
1432	2017	CCHF BY PCR	SINDH	Karachi	NEGATIVE
1433	2017	CCHF BY PCR	Baluchistan	Quetta	POSITIVE
1434	2017	CCHF BY PCR	KPK	Peshawar	POSITIVE
1435	2017	CCHF BY PCR	PUNJAB	Lahore	NEGATIVE
1436	2017	CCHF BY PCR	Baluchistan	Quetta	NEGATIVE
1437	2017	CCHF BY PCR	Baluchistan	Quetta	POSITIVE
1438	2017	CCHF BY PCR	KPK	Peshawar	POSITIVE
1439	2017	CCHF BY PCR	KPK	Peshawar	NEGATIVE
1440	2017	CCHF BY PCR	Baluchistan	Quetta	POSITIVE
1441	2017	CCHF BY PCR	KPK	KOHAT	NEGATIVE
1442	2017	CCHF BY PCR	Baluchistan	Quetta	POSITIVE
1443	2017	CCHF BY PCR	Baluchistan	Quetta	NEGATIVE
1444	2017	CCHF BY PCR	PUNJAB	Lahore	NEGATIVE
1445	2017	CCHF BY PCR	KPK	Peshawar	NEGATIVE
1446	2017	CCHF BY PCR	KPK	Peshawar	POSITIVE
1447	2017	CCHF BY PCR	Baluchistan	Quetta	POSITIVE
1448	2017	CCHF BY PCR	Baluchistan	Quetta	NEGATIVE

1449	2017	CCHF BY PCR	Baluchistan	Quetta	NEGATIVE
1450	2017	CCHF BY PCR	Baluchistan	Quetta	NEGATIVE
1451	2017	CCHF BY PCR	PUNJAB	Islamabad	NEGATIVE
1452	2017	CCHF BY PCR	Baluchistan	Quetta	POSITIVE
1453	2017	CCHF BY PCR	Baluchistan	Quetta	POSITIVE
1454	2017	CCHF BY PCR	KPK	Peshawar	NEGATIVE
1455	2017	CCHF BY PCR	KPK	Peshawar	NEGATIVE
1456	2017	CCHF BY PCR	Baluchistan	Quetta	POSITIVE
1457	2017	CCHF BY PCR	KPK	Peshawar	NEGATIVE
1458	2017	CCHF BY PCR	Baluchistan	Quetta	NEGATIVE
1459	2017	CCHF BY PCR	KPK	Peshawar	POSITIVE
1460	2017	CCHF BY PCR	Baluchistan	Quetta	NEGATIVE
1461	2017	CCHF BY PCR	Baluchistan	Quetta	NEGATIVE
1462	2017	CCHF BY PCR	Baluchistan	Quetta	POSITIVE
1463	2017	CCHF BY PCR	KPK	Peshawar	NEGATIVE
1464	2017	CCHF BY PCR	Baluchistan	Quetta	POSITIVE
1465	2017	CCHF BY PCR	KPK	Peshawar	NEGATIVE
1466	2017	CCHF BY PCR	SINDH	Karachi	NEGATIVE
1467	2017	CCHF BY PCR	KPK	Peshawar	NEGATIVE
1468	2017	CCHF BY PCR	Baluchistan	Quetta	NEGATIVE
1469	2017	CCHF BY PCR	KPK	Peshawar	POSITIVE
1470	2017	CCHF BY PCR	Baluchistan	Quetta	NEGATIVE
1471	2017	CCHF BY PCR	SINDH	Karachi	NEGATIVE
1472	2017	CCHF BY PCR	SINDH	Karachi	NEGATIVE
1473	2017	CCHF BY PCR	Baluchistan	Quetta	NEGATIVE
1474	2017	CCHF BY PCR	Baluchistan	Quetta	NEGATIVE
1475	2017	CCHF BY PCR	SINDH	Hyderabad	NEGATIVE
1476	2017	CCHF BY PCR	SINDH	Karachi	NEGATIVE
1477	2017	CCHF BY PCR	KPK	Peshawar	NEGATIVE
1478	2017	CCHF BY PCR	SINDH	Karachi	POSITIVE
1479	2017	CCHF BY PCR	Baluchistan	Quetta	NEGATIVE
1480	2017	CCHF BY PCR	Baluchistan	Quetta	POSITIVE
1481	2017	CCHF BY PCR	Baluchistan	Quetta	POSITIVE

1482	2017	CCHF BY PCR	KPK	Peshawar	NEGATIVE
1483	2017	CCHF BY PCR	Baluchistan	Quetta	NEGATIVE
1484	2017	CCHF BY PCR	KPK	Peshawar	NEGATIVE
1485	2017	CCHF BY PCR	KPK	Peshawar	NEGATIVE
1486	2017	CCHF BY PCR	KPK	Peshawar	POSITIVE
1487	2017	CCHF BY PCR	PUNJAB	Gujar Khan	NEGATIVE
1488	2017	CCHF BY PCR	Baluchistan	Quetta	NEGATIVE
1489	2017	CCHF BY PCR	Baluchistan	Quetta	NEGATIVE
1490	2017	CCHF BY PCR	Baluchistan	Quetta	NEGATIVE
1491	2017	CCHF BY PCR	Baluchistan	Quetta	POSITIVE
1492	2017	CCHF BY PCR	KPK	Peshawar	NEGATIVE
1493	2017	CCHF BY PCR	PUNJAB	Lahore	NEGATIVE
1494	2017	CCHF BY PCR	Baluchistan	Quetta	NEGATIVE
1495	2017	CCHF BY PCR	KPK	Peshawar	POSITIVE
1496	2017	CCHF BY PCR	Baluchistan	Quetta	NEGATIVE
1497	2017	CCHF BY PCR	Baluchistan	Quetta	NEGATIVE
1498	2017	CCHF BY PCR	Baluchistan	Quetta	NEGATIVE
1499	2017	CCHF BY PCR	KPK	Peshawar	NEGATIVE
1500	2017	CCHF BY PCR	PUNJAB	Lahore	NEGATIVE
1501	2017	CCHF BY PCR	SINDH	Karachi	NEGATIVE
1502	2017	CCHF BY PCR	SINDH	Karachi	NEGATIVE
1503	2017	CCHF BY PCR	Baluchistan	Loralai	NEGATIVE
1504	2017	CCHF BY PCR	SINDH	Karachi	NEGATIVE
1505	2017	CCHF BY PCR	PUNJAB	Lahore	NEGATIVE
1506	2017	CCHF BY PCR	KPK	Peshawar	NEGATIVE
1507	2017	CCHF BY PCR	SINDH	Karachi	NEGATIVE
1508	2017	CCHF BY PCR	KPK	Peshawar	NEGATIVE
1509	2017	CCHF BY PCR	Baluchistan	Quetta	NEGATIVE
1510	2017	CCHF BY PCR	SINDH	Karachi	NEGATIVE
1511	2017	CCHF BY PCR	PUNJAB	Islamabad	NEGATIVE
1512	2017	CCHF BY PCR	Baluchistan	Quetta	POSITIVE
1513	2017	CCHF BY PCR	PUNJAB	Lahore	NEGATIVE
1514	2017	CCHF BY PCR	Baluchistan	Quetta	NEGATIVE
1515	2017	CCHF BY PCR	Baluchistan	Quetta	NEGATIVE

1516	2017	CCHF BY PCR	Baluchistan	Quetta	NEGATIVE
1517	2017	CCHF BY PCR	KPK	Peshawar	NEGATIVE
1518	2017	CCHF BY PCR	KPK	Peshawar	NEGATIVE
1519	2017	CCHF BY PCR	KPK	Peshawar	NEGATIVE
1520	2017	CCHF BY PCR	PUNJAB	Islamabad	POSITIVE
1521	2017	CCHF BY PCR	PUNJAB	Islamabad	NEGATIVE
1522	2017	CCHF BY PCR	KPK	Abbottabad	NEGATIVE
1523	2017	CCHF BY PCR	KPK	Peshawar	NEGATIVE
1524	2017	CCHF BY PCR	KPK	Peshawar	NEGATIVE
1525	2017	CCHF BY PCR	Baluchistan	Quetta	POSITIVE
1526	2017	CCHF BY PCR	Baluchistan	Quetta	POSITIVE
1527	2017	CCHF BY PCR	Baluchistan	Quetta	POSITIVE
1528	2017	CCHF BY PCR	Baluchistan	Quetta	POSITIVE
1529	2017	CCHF BY PCR	PUNJAB	Islamabad	NEGATIVE
1530	2017	CCHF BY PCR	KPK	Peshawar	NEGATIVE
1531	2017	CCHF BY PCR	KPK	Peshawar	NEGATIVE
1532	2017	CCHF BY PCR	Baluchistan	Quetta	NEGATIVE
1533	2017	CCHF BY PCR	Baluchistan	Quetta	NEGATIVE
1534	2017	CCHF BY PCR	SINDH	Karachi	POSITIVE
1535	2017	CCHF BY PCR	KPK	Peshawar	NEGATIVE
1536	2017	CCHF BY PCR	Baluchistan	Quetta	NEGATIVE
1537	2017	CCHF BY PCR	Baluchistan	Quetta	NEGATIVE
1538	2017	CCHF BY PCR	Baluchistan	Quetta	NEGATIVE
1539	2017	CCHF BY PCR	Baluchistan	Quetta	NEGATIVE
1540	2017	CCHF BY PCR	PUNJAB	Rawalpindi	NEGATIVE
1541	2017	CCHF BY PCR	KPK	Peshawar	NEGATIVE
1542	2017	CCHF BY PCR	KPK	Peshawar	POSITIVE
1543	2017	CCHF BY PCR	KPK	Peshawar	NEGATIVE
1544	2017	CCHF BY PCR	SINDH	Karachi	NEGATIVE
1545	2017	CCHF BY PCR	Baluchistan	Quetta	POSITIVE
1546	2017	CCHF BY PCR	Baluchistan	Quetta	POSITIVE
1547	2017	CCHF BY PCR	SINDH	Karachi	NEGATIVE
1548	2017	CCHF BY PCR	PUNJAB	Lahore	NEGATIVE
1549	2017	CCHF BY PCR	KPK	Peshawar	POSITIVE
1550	2017	CCHF BY PCR	SINDH	Karachi	NEGATIVE
1551	2017	CCHF BY PCR	SINDH	Karachi	NEGATIVE

1552	2017	CCHF BY PCR	PUNJAB	Islamabad	NEGATIVE
1553	2017	CCHF BY PCR	Baluchistan	Quetta	POSITIVE
1554	2017	CCHF BY PCR	SINDH	Karachi	NEGATIVE
1555	2017	CCHF BY PCR	PUNJAB	Lahore	NEGATIVE
1556	2017	CCHF BY PCR	SINDH	Karachi	NEGATIVE
1557	2017	CCHF BY PCR	SINDH	Karachi	NEGATIVE
1558	2017	CCHF BY PCR	SINDH	Karachi	NEGATIVE
1559	2017	CCHF BY PCR	SINDH	Karachi	NEGATIVE
1560	2017	CCHF BY PCR	Baluchistan	Quetta	NEGATIVE
1561	2017	CCHF BY PCR	Baluchistan	Quetta	NEGATIVE
1562	2017	CCHF BY PCR	SINDH	Karachi	NEGATIVE
1563	2017	CCHF BY PCR	PUNJAB	Bahawalpur	NEGATIVE
1564	2017	CCHF BY PCR	Baluchistan	Quetta	NEGATIVE
1565	2017	CCHF BY PCR	KPK	Peshawar	POSITIVE
1566	2017	CCHF BY PCR	PUNJAB	DERA GHAZI KHAN	NEGATIVE
1567	2017	CCHF BY PCR	KPK	Peshawar	NEGATIVE
1568	2017	CCHF BY PCR	Baluchistan	Quetta	NEGATIVE
1569	2017	CCHF BY PCR	KPK	Peshawar	POSITIVE
1570	2017	CCHF BY PCR	KPK	Timargarh	NEGATIVE
1571	2017	CCHF BY PCR	SINDH	Karachi	POSITIVE
1572	2017	CCHF BY PCR	Baluchistan	Quetta	NEGATIVE
1573	2017	CCHF BY PCR	Baluchistan	Quetta	POSITIVE
1574	2017	CCHF BY PCR	KPK	Peshawar	NEGATIVE
1575	2017	CCHF BY PCR	SINDH	Karachi	NEGATIVE
1576	2017	CCHF BY PCR	Baluchistan	Quetta	POSITIVE
1577	2017	CCHF BY PCR	Baluchistan	Quetta	POSITIVE
1578	2017	CCHF BY PCR	Baluchistan	Quetta	POSITIVE
1579	2017	CCHF BY PCR	Baluchistan	Quetta	NEGATIVE
1580	2017	CCHF BY PCR	SINDH	Karachi	NEGATIVE
1581	2017	CCHF BY PCR	SINDH	Karachi	NEGATIVE
1582	2017	CCHF BY PCR	SINDH	Karachi	NEGATIVE
1583	2017	CCHF BY PCR	Baluchistan	Quetta	NEGATIVE
1584	2017	CCHF BY PCR	KPK	Peshawar	POSITIVE
1585	2017	CCHF BY PCR	Baluchistan	Quetta	POSITIVE
1586	2017	CCHF BY PCR	PUNJAB	Faisalabad	NEGATIVE

1587	2017	CCHF BY PCR	Baluchistan	Quetta	NEGATIVE
1588	2017	CCHF BY PCR	SINDH	Karachi	NEGATIVE
1589	2017	CCHF BY PCR	SINDH	Karachi	NEGATIVE
1590	2017	CCHF BY PCR	KPK	Peshawar	NEGATIVE
1591	2017	CCHF BY PCR	SINDH	Karachi	NEGATIVE
1592	2018	CCHF BY PCR	Baluchistan	Quetta	NEGATIVE
1593	2018	CCHF BY PCR	Baluchistan	Quetta	NEGATIVE
1594	2018	CCHF BY PCR	Baluchistan	Quetta	NEGATIVE
1595	2018	CCHF BY PCR	SINDH	Karachi	NEGATIVE
1596	2018	CCHF BY PCR	Baluchistan	Quetta	NEGATIVE
1597	2018	CCHF BY PCR	Baluchistan	Quetta	NEGATIVE
1598	2018	CCHF BY PCR	Baluchistan	Quetta	NEGATIVE
1599	2018	CCHF BY PCR	Baluchistan	Quetta	NEGATIVE
1600	2021	CCHF BY PCR	Baluchistan	Quetta	NEGATIVE
1601	2018	CCHF BY PCR	KPK	Peshawar	NEGATIVE
1602	2018	CCHF BY PCR	KPK	Peshawar	POSITIVE
1603	2018	CCHF BY PCR	Baluchistan	Quetta	NEGATIVE
1604	2018	CCHF BY PCR	KPK	Peshawar	NEGATIVE
1605	2018	CCHF BY PCR	KPK	Peshawar	NEGATIVE
1606	2018	CCHF BY PCR	Baluchistan	Quetta	NEGATIVE
1607	2018	CCHF BY PCR	Baluchistan	Quetta	NEGATIVE
1608	2018	CCHF BY PCR	SINDH	Karachi	NEGATIVE
1609	2018	CCHF BY PCR	Baluchistan	Quetta	NEGATIVE
1610	2018	CCHF BY PCR	SINDH	Karachi	NEGATIVE
1611	2018	CCHF BY PCR	Baluchistan	Quetta	POSITIVE
1612	2018	CCHF BY PCR	SINDH	Karachi	POSITIVE
1613	2018	CCHF BY PCR	SINDH	Karachi	NEGATIVE
1614	2018	CCHF BY PCR	SINDH	Karachi	POSITIVE
1615	2018	CCHF BY PCR	KPK	Peshawar	POSITIVE
1616	2018	CCHF BY PCR	KPK	Peshawar	POSITIVE
1617	2018	CCHF BY PCR	Baluchistan	Quetta	POSITIVE
1618	2018	CCHF BY PCR	Baluchistan	Quetta	NEGATIVE
1619	2018	CCHF BY PCR	KPK	Peshawar	POSITIVE
1620	2018	CCHF BY PCR	KPK	Peshawar	POSITIVE

1621	2018	CCHF BY PCR	SINDH	Karachi	POSITIVE
1622	2018	CCHF BY PCR	SINDH	Karachi	NEGATIVE
1623	2018	CCHF BY PCR	Baluchistan	Quetta	POSITIVE
1624	2018	CCHF BY PCR	KPK	Peshawar	NEGATIVE
1625	2018	CCHF BY PCR	KPK	Peshawar	NEGATIVE
1626	2018	CCHF BY PCR	KPK	Peshawar	NEGATIVE
1627	2018	CCHF BY PCR	KPK	Peshawar	NEGATIVE
1628	2018	CCHF BY PCR	Baluchistan	Quetta	POSITIVE
1629	2018	CCHF BY PCR	SINDH	Karachi	POSITIVE
1630	2018	CCHF BY PCR	KPK	Peshawar	POSITIVE
1631	2018	CCHF BY PCR	Baluchistan	Quetta	NEGATIVE
1632	2018	CCHF BY PCR	KPK	Peshawar	NEGATIVE
1633	2018	CCHF BY PCR	SINDH	Karachi	NEGATIVE
1634	2018	CCHF BY PCR	KPK	Peshawar	NEGATIVE
1635	2018	CCHF BY PCR	SINDH	Karachi	NEGATIVE
1636	2018	CCHF BY PCR	Baluchistan	Quetta	POSITIVE
1637	2018	CCHF BY PCR	KPK	Peshawar	POSITIVE
1638	2018	CCHF BY PCR	SINDH	Hyderabad	NEGATIVE
1639	2018	CCHF BY PCR	Baluchistan	Quetta	NEGATIVE
1640	2018	CCHF BY PCR	SINDH	Karachi	NEGATIVE
1641	2018	CCHF BY PCR	Baluchistan	Quetta	NEGATIVE
1642	2018	CCHF BY PCR	SINDH	Karachi	NEGATIVE
1643	2018	CCHF BY PCR	SINDH	Karachi	NEGATIVE
1644	2018	CCHF BY PCR	SINDH	Karachi	NEGATIVE
1645	2018	CCHF BY PCR	Baluchistan	Quetta	NEGATIVE
1646	2018	CCHF BY PCR	KPK	Peshawar	POSITIVE
1647	2018	CCHF BY PCR	KPK	Peshawar	POSITIVE
1648	2018	CCHF BY PCR	Baluchistan	Quetta	POSITIVE
1649	2018	CCHF BY PCR	Baluchistan	Quetta	POSITIVE
1650	2018	CCHF BY PCR	Baluchistan	Quetta	NEGATIVE
1651	2018	CCHF BY PCR	Baluchistan	Quetta	POSITIVE
1652	2018	CCHF BY PCR	SINDH	Karachi	NEGATIVE
1653	2018	CCHF BY PCR	SINDH	Karachi	NEGATIVE
1654	2018	CCHF BY PCR	SINDH	Karachi	NEGATIVE
1655	2018	CCHF BY PCR	Baluchistan	Quetta	POSITIVE
1656	2018	CCHF BY PCR	SINDH	Karachi	NEGATIVE

1657	2018	CCHF BY PCR	Baluchistan	Quetta	POSITIVE
1658	2018	CCHF BY PCR	KPK	Peshawar	NEGATIVE
1659	2018	CCHF BY PCR	SINDH	Karachi	NEGATIVE
1660	2018	CCHF BY PCR	Baluchistan	Quetta	NEGATIVE
1661	2018	CCHF BY PCR	Baluchistan	Quetta	POSITIVE
1662	2018	CCHF BY PCR	SINDH	Karachi	NEGATIVE
1663	2018	CCHF BY PCR	Baluchistan	Quetta	POSITIVE
1664	2018	CCHF BY PCR	Baluchistan	Quetta	POSITIVE
1665	2018	CCHF BY PCR	Baluchistan	Quetta	NEGATIVE
1666	2018	CCHF BY PCR	Baluchistan	Quetta	NEGATIVE
1667	2018	CCHF BY PCR	Baluchistan	Quetta	NEGATIVE
1668	2018	CCHF BY PCR	Baluchistan	Quetta	NEGATIVE
1669	2018	CCHF BY PCR	SINDH	Karachi	NEGATIVE
1670	2018	CCHF BY PCR	Baluchistan	Quetta	NEGATIVE
1671	2018	CCHF BY PCR	KPK	Peshawar	NEGATIVE
1672	2018	CCHF BY PCR	SINDH	Karachi	NEGATIVE
1673	2018	CCHF BY PCR	Baluchistan	Quetta	NEGATIVE
1674	2018	CCHF BY PCR	Baluchistan	Quetta	NEGATIVE
1675	2018	CCHF BY PCR	KPK	Peshawar	NEGATIVE
1676	2018	CCHF BY PCR	KPK	Peshawar	NEGATIVE
1677	2018	CCHF BY PCR	KPK	Peshawar	NEGATIVE
1678	2018	CCHF BY PCR	Baluchistan	Quetta	NEGATIVE
1679	2018	CCHF BY PCR	KPK	Peshawar	NEGATIVE
1680	2018	CCHF BY PCR	SINDH	Karachi	NEGATIVE
1681	2018	CCHF BY PCR	SINDH	Karachi	NEGATIVE
1682	2018	CCHF BY PCR	SINDH	Karachi	POSITIVE
1683	2018	CCHF BY PCR	PUNJAB	RAHIM YAR KHAN	NEGATIVE
1684	2018	CCHF BY PCR	Baluchistan	Quetta	NEGATIVE
1685	2018	CCHF BY PCR	SINDH	Karachi	NEGATIVE
1686	2018	CCHF BY PCR	Baluchistan	Quetta	POSITIVE
1687	2018	CCHF BY PCR	Baluchistan	Quetta	NEGATIVE
1688	2018	CCHF BY PCR	PUNJAB	Lahore	NEGATIVE

1689	2018	CCHF BY PCR	Baluchistan	Quetta	NEGATIVE
1690	2018	CCHF BY PCR	KPK	Peshawar	NEGATIVE
1691	2018	CCHF BY PCR	Baluchistan	Quetta	NEGATIVE
1692	2018	CCHF BY PCR	SINDH	Karachi	NEGATIVE
1693	2018	CCHF BY PCR	KPK	Peshawar	NEGATIVE
1694	2018	CCHF BY PCR	SINDH	Karachi	NEGATIVE
1695	2018	CCHF BY PCR	KPK	Peshawar	POSITIVE
1696	2018	CCHF BY PCR	KPK	Peshawar	POSITIVE
1697	2018	CCHF BY PCR	SINDH	Karachi	NEGATIVE
1698	2018	CCHF BY PCR	SINDH	Karachi	POSITIVE
1699	2018	CCHF BY PCR	KPK	Peshawar	NEGATIVE
1700	2018	CCHF BY PCR	Baluchistan	Quetta	POSITIVE
1701	2018	CCHF BY PCR	Baluchistan	Quetta	POSITIVE
1702	2018	CCHF BY PCR	Baluchistan	Quetta	NEGATIVE
1703	2018	CCHF BY PCR	Baluchistan	Quetta	POSITIVE
1704	2018	CCHF BY PCR	SINDH	Karachi	NEGATIVE
1705	2018	CCHF BY PCR	Baluchistan	Quetta	POSITIVE
1706	2018	CCHF BY PCR	SINDH	Karachi	POSITIVE
1707	2018	CCHF BY PCR	SINDH	Karachi	POSITIVE
1708	2018	CCHF BY PCR	Baluchistan	Quetta	POSITIVE
1709	2018	CCHF BY PCR	SINDH	Karachi	NEGATIVE
1710	2018	CCHF BY PCR	SINDH	Karachi	NEGATIVE
1711	2018	CCHF BY PCR	SINDH	Karachi	NEGATIVE
1712	2018	CCHF BY PCR	Baluchistan	Quetta	POSITIVE
1713	2018	CCHF BY PCR	SINDH	Karachi	NEGATIVE
1714	2018	CCHF BY PCR	Baluchistan	Quetta	POSITIVE
1715	2018	CCHF BY PCR	SINDH	Karachi	NEGATIVE
1716	2018	CCHF BY PCR	Baluchistan	Quetta	POSITIVE
1717	2018	CCHF BY PCR	KPK	Peshawar	POSITIVE
1718	2018	CCHF BY PCR	Baluchistan	Quetta	POSITIVE
1719	2018	CCHF BY PCR	Baluchistan	Quetta	POSITIVE
1720	2018	CCHF BY PCR	SINDH	Karachi	POSITIVE
1721	2018	CCHF BY PCR	Baluchistan	Quetta	POSITIVE
1722	2018	CCHF BY PCR	SINDH	Karachi	POSITIVE
1723	2018	CCHF BY PCR	SINDH	Karachi	NEGATIVE

1724	2018	CCHF BY PCR	SINDH	Karachi	NEGATIVE
1725	2018	CCHF BY PCR	SINDH	Karachi	NEGATIVE
1726	2018	CCHF BY PCR	KPK	Peshawar	NEGATIVE
1727	2018	CCHF BY PCR	Baluchistan	Quetta	NEGATIVE
1728	2018	CCHF BY PCR	Baluchistan	Quetta	NEGATIVE
1729	2018	CCHF BY PCR	Baluchistan	Quetta	NEGATIVE
1730	2018	CCHF BY PCR	KPK	Peshawar	NEGATIVE
1731	2018	CCHF BY PCR	KPK	Peshawar	NEGATIVE
1732	2018	CCHF BY PCR	Baluchistan	Quetta	NEGATIVE
1733	2018	CCHF BY PCR	SINDH	Karachi	NEGATIVE
1734	2018	CCHF BY PCR	Baluchistan	Quetta	NEGATIVE
1735	2018	CCHF BY PCR	SINDH	Karachi	NEGATIVE
1736	2018	CCHF BY PCR	Baluchistan	Quetta	NEGATIVE
1737	2018	CCHF BY PCR	Baluchistan	Quetta	NEGATIVE
1738	2018	CCHF BY PCR	Baluchistan	Quetta	NEGATIVE
1739	2018	CCHF BY PCR	Baluchistan	Quetta	NEGATIVE
1740	2018	CCHF BY PCR	Baluchistan	Quetta	NEGATIVE
1741	2018	CCHF BY PCR	Baluchistan	Quetta	NEGATIVE
1742	2018	CCHF BY PCR	Baluchistan	Quetta	NEGATIVE
1743	2018	CCHF BY PCR	SINDH	Karachi	NEGATIVE
1744	2018	CCHF BY PCR	SINDH	Karachi	POSITIVE
1745	2018	CCHF BY PCR	SINDH	Karachi	POSITIVE
1746	2018	CCHF BY PCR	Baluchistan	Quetta	NEGATIVE
1747	2018	CCHF BY PCR	SINDH	Karachi	NEGATIVE
1748	2018	CCHF BY PCR	Baluchistan	Quetta	NEGATIVE
1749	2018	CCHF BY PCR	PUNJAB	Mianwali	NEGATIVE
1750	2018	CCHF BY PCR	KPK	Abbottabad	NEGATIVE
1751	2018	CCHF BY PCR	Baluchistan	Quetta	NEGATIVE
1752	2018	CCHF BY PCR	Baluchistan	Quetta	NEGATIVE
1753	2018	CCHF BY PCR	SINDH	Karachi	NEGATIVE
1754	2018	CCHF BY PCR	SINDH	Karachi	NEGATIVE
1755	2018	CCHF BY PCR	Baluchistan	Quetta	POSITIVE

1756	2018	CCHF BY PCR	Baluchistan	Quetta	NEGATIVE
1757	2018	CCHF BY PCR	SINDH	Karachi	NEGATIVE
1758	2018	CCHF BY PCR	SINDH	Karachi	NEGATIVE
1759	2018	CCHF BY PCR	SINDH	Karachi	NEGATIVE
1760	2018	CCHF BY PCR	Baluchistan	Quetta	NEGATIVE
1761	2018	CCHF BY PCR	SINDH	Karachi	NEGATIVE
1762	2018	CCHF BY PCR	KPK	Peshawar	NEGATIVE
1763	2018	CCHF BY PCR	KPK	Peshawar	POSITIVE
1764	2018	CCHF BY PCR	SINDH	Karachi	NEGATIVE
1765	2018	CCHF BY PCR	SINDH	Karachi	NEGATIVE
1766	2018	CCHF BY PCR	KPK	Peshawar	NEGATIVE
1767	2018	CCHF BY PCR	SINDH	Karachi	NEGATIVE
1768	2018	CCHF BY PCR	Baluchistan	Quetta	POSITIVE
1769	2018	CCHF BY PCR	SINDH	Karachi	NEGATIVE
1770	2018	CCHF BY PCR	SINDH	Karachi	NEGATIVE
1771	2018	CCHF BY PCR	KPK	Peshawar	NEGATIVE
1772	2018	CCHF BY PCR	Baluchistan	Quetta	NEGATIVE
1773	2018	CCHF BY PCR	SINDH	Karachi	NEGATIVE
1774	2018	CCHF BY PCR	PUNJAB	Lahore	NEGATIVE
1775	2018	CCHF BY PCR	Baluchistan	Quetta	NEGATIVE
1776	2018	CCHF BY PCR	Baluchistan	Quetta	NEGATIVE
1777	2018	CCHF BY PCR	SINDH	Karachi	NEGATIVE
1778	2018	CCHF BY PCR	KPK	Peshawar	NEGATIVE
1779	2018	CCHF BY PCR	SINDH	Karachi	POSITIVE
1780	2018	CCHF BY PCR	KPK	Peshawar	NEGATIVE
1781	2018	CCHF BY PCR	SINDH	Karachi	NEGATIVE
1782	2018	CCHF BY PCR	Baluchistan	Quetta	NEGATIVE
1783	2018	CCHF BY PCR	Baluchistan	Quetta	NEGATIVE
1784	2018	CCHF BY PCR	SINDH	Karachi	POSITIVE
1785	2018	CCHF BY PCR	Baluchistan	Quetta	NEGATIVE
1786	2018	CCHF BY PCR	Baluchistan	Quetta	NEGATIVE
1787	2018	CCHF BY PCR	SINDH	Karachi	NEGATIVE
1788	2018	CCHF BY PCR	SINDH	Karachi	NEGATIVE
1789	2018	CCHF BY PCR	Baluchistan	Quetta	NEGATIVE
1790	2018	CCHF BY PCR	SINDH	Karachi	NEGATIVE
1791	2018	CCHF BY PCR	KPK	Peshawar	NEGATIVE
1792	2018	CCHF BY PCR	Baluchistan	Quetta	NEGATIVE

1793	2018	CCHF BY PCR	SINDH	Hyderabad	POSITIVE
1794	2018	CCHF BY PCR	Baluchistan	Quetta	POSITIVE
1795	2018	CCHF BY PCR	SINDH	Karachi	NEGATIVE
1796	2018	CCHF BY PCR	Baluchistan	Quetta	NEGATIVE
1797	2018	CCHF BY PCR	KPK	Peshawar	NEGATIVE
1798	2018	CCHF BY PCR	Baluchistan	Quetta	POSITIVE
1799	2018	CCHF BY PCR	KPK	Dera Ismail Khan	NEGATIVE
1800	2018	CCHF BY PCR	SINDH	Karachi	NEGATIVE
1801	2018	CCHF BY PCR	KPK	Peshawar	NEGATIVE
1802	2018	CCHF BY PCR	Baluchistan	Quetta	NEGATIVE
1803	2018	CCHF BY PCR	Baluchistan	Quetta	POSITIVE
1804	2018	CCHF BY PCR	KPK	Peshawar	POSITIVE
1805	2018	CCHF BY PCR	KPK	Abbottabad	NEGATIVE
1806	2018	CCHF BY PCR	KPK	Peshawar	NEGATIVE
1807	2018	CCHF BY PCR	SINDH	Karachi	NEGATIVE
1808	2018	CCHF BY PCR	PUNJAB	RAHIM YAR KHAN	NEGATIVE
1809	2018	CCHF BY PCR	Baluchistan	Quetta	POSITIVE
1810	2018	CCHF BY PCR	Baluchistan	Quetta	NEGATIVE
1811	2018	CCHF BY PCR	SINDH	Karachi	POSITIVE
1812	2018	CCHF BY PCR	SINDH	Karachi	NEGATIVE
1813	2018	CCHF BY PCR	SINDH	Karachi	NEGATIVE
1814	2018	CCHF BY PCR	SINDH	Karachi	NEGATIVE
1815	2018	CCHF BY PCR	Baluchistan	Quetta	NEGATIVE
1816	2018	CCHF BY PCR	Baluchistan	Quetta	NEGATIVE
1817	2018	CCHF BY PCR	SINDH	Karachi	POSITIVE
1818	2018	CCHF BY PCR	SINDH	Karachi	NEGATIVE
1819	2018	CCHF BY PCR	KPK	Peshawar	NEGATIVE
1820	2018	CCHF BY PCR	SINDH	Karachi	NEGATIVE
1821	2018	CCHF BY PCR	SINDH	Karachi	NEGATIVE
1822	2018	CCHF BY PCR	SINDH	Karachi	NEGATIVE
1823	2018	CCHF BY PCR	KPK	Peshawar	NEGATIVE
1824	2018	CCHF BY PCR	Baluchistan	Quetta	POSITIVE
1825	2018	CCHF BY PCR	SINDH	Karachi	POSITIVE
1826	2018	CCHF BY PCR	Baluchistan	Quetta	POSITIVE
1827	2018	CCHF BY PCR	Baluchistan	Quetta	NEGATIVE
1828	2018	CCHF BY PCR	KPK	Peshawar	POSITIVE

1829	2018	CCHF BY PCR	SINDH	Karachi	NEGATIVE
1830	2018	CCHF BY PCR	SINDH	Karachi	NEGATIVE
1831	2018	CCHF BY PCR	Baluchistan	Quetta	NEGATIVE
1832	2018	CCHF BY PCR	SINDH	Karachi	NEGATIVE
1833	2018	CCHF BY PCR	SINDH	Karachi	NEGATIVE
1834	2018	CCHF BY PCR	Baluchistan	Quetta	NEGATIVE
1835	2018	CCHF BY PCR	SINDH	Karachi	NEGATIVE
1836	2018	CCHF BY PCR	SINDH	Karachi	NEGATIVE
1837	2018	CCHF BY PCR	SINDH	Karachi	POSITIVE
1838	2018	CCHF BY PCR	Baluchistan	Quetta	POSITIVE
1839	2018	CCHF BY PCR	SINDH	Karachi	NEGATIVE
1840	2018	CCHF BY PCR	Baluchistan	Quetta	NEGATIVE
1841	2018	CCHF BY PCR	SINDH	Karachi	NEGATIVE
1842	2018	CCHF BY PCR	SINDH	Karachi	NEGATIVE
1843	2018	CCHF BY PCR	SINDH	Karachi	NEGATIVE
1844	2018	CCHF BY PCR	SINDH	Karachi	NEGATIVE
1845	2018	CCHF BY PCR	Baluchistan	Quetta	NEGATIVE
1846	2018	CCHF BY PCR	Baluchistan	Quetta	NEGATIVE
1847	2018	CCHF BY PCR	Baluchistan	Quetta	NEGATIVE
1848	2018	CCHF BY PCR	SINDH	Karachi	NEGATIVE
1849	2018	CCHF BY PCR	SINDH	Karachi	NEGATIVE
1850	2018	CCHF BY PCR	KPK	Peshawar	NEGATIVE
1851	2018	CCHF BY PCR	PUNJAB	Kharian	NEGATIVE
1852	2018	CCHF BY PCR	SINDH	Karachi	NEGATIVE
1853	2018	CCHF BY PCR	SINDH	Karachi	NEGATIVE
1854	2018	CCHF BY PCR	Baluchistan	Quetta	NEGATIVE
1855	2018	CCHF BY PCR	SINDH	Karachi	NEGATIVE
1856	2018	CCHF BY PCR	Baluchistan	Quetta	NEGATIVE
1857	2019	CCHF BY PCR	KPK	Peshawar	NEGATIVE
1858	2019	CCHF BY PCR	SINDH	Karachi	NEGATIVE
1859	2019	CCHF BY PCR	SINDH	Karachi	NEGATIVE
1860	2019	CCHF BY PCR	KPK	Peshawar	NEGATIVE
1861	2019	CCHF BY PCR	SINDH	Karachi	NEGATIVE
1862	2019	CCHF BY PCR	SINDH	Karachi	NEGATIVE
1863	2019	CCHF BY PCR	SINDH	Karachi	NEGATIVE
1864	2019	CCHF BY PCR	SINDH	Karachi	NEGATIVE
1865	2019	CCHF BY PCR	SINDH	Karachi	NEGATIVE
1866	2019	CCHF BY PCR	SINDH	Karachi	NEGATIVE
1867	2019	CCHF BY PCR	PUNJAB	Lahore	NEGATIVE

1868	2019	CCHF BY PCR	SINDH	Karachi	NEGATIVE
1869	2019	CCHF BY PCR	SINDH	Karachi	NEGATIVE
1870	2019	CCHF BY PCR	Baluchistan	Quetta	NEGATIVE
1871	2019	CCHF BY PCR	SINDH	Karachi	NEGATIVE
1872	2019	CCHF BY PCR	KPK	Peshawar	NEGATIVE
1873	2019	CCHF BY PCR	Baluchistan	Quetta	NEGATIVE
1874	2019	CCHF BY PCR	SINDH	Hyderabad	NEGATIVE
1875	2019	CCHF BY PCR	Baluchistan	Quetta	NEGATIVE
1876	2019	CCHF BY PCR	KPK	Peshawar	NEGATIVE
1877	2019	CCHF BY PCR	SINDH	Karachi	POSITIVE
1878	2019	CCHF BY PCR	SINDH	Karachi	NEGATIVE
1879	2019	CCHF BY PCR	SINDH	Karachi	NEGATIVE
1880	2019	CCHF BY PCR	PUNJAB	Gujranwala	NEGATIVE
1881	2019	CCHF BY PCR	SINDH	Karachi	NEGATIVE
1882	2019	CCHF BY PCR	SINDH	Karachi	NEGATIVE
1883	2019	CCHF BY PCR	Baluchistan	Quetta	NEGATIVE
1884	2019	CCHF BY PCR	KPK	Peshawar	NEGATIVE
1885	2019	CCHF BY PCR	SINDH	Karachi	NEGATIVE
1886	2019	CCHF BY PCR	SINDH	Karachi	NEGATIVE
1887	2019	CCHF BY PCR	Baluchistan	Quetta	NEGATIVE
1888	2019	CCHF BY PCR	SINDH	Karachi	NEGATIVE
1889	2019	CCHF BY PCR	SINDH	Karachi	POSITIVE
1890	2019	CCHF BY PCR	SINDH	Karachi	NEGATIVE
1891	2019	CCHF BY PCR	Baluchistan	Quetta	POSITIVE
1892	2019	CCHF BY PCR	Baluchistan	Quetta	POSITIVE
1893	2019	CCHF BY PCR	SINDH	Karachi	NEGATIVE
1894	2019	CCHF BY PCR	Baluchistan	Quetta	NEGATIVE
1895	2019	CCHF BY PCR	Baluchistan	Quetta	POSITIVE
1896	2019	CCHF BY PCR	SINDH	Karachi	NEGATIVE
1897	2019	CCHF BY PCR	SINDH	Karachi	NEGATIVE
1898	2019	CCHF BY PCR	SINDH	Karachi	NEGATIVE
1899	2019	CCHF BY PCR	SINDH	Karachi	POSITIVE
1900	2019	CCHF BY PCR	SINDH	Hyderabad	NEGATIVE
1901	2019	CCHF BY PCR	SINDH	Karachi	NEGATIVE
1902	2019	CCHF BY PCR	Baluchistan	Quetta	NEGATIVE
1903	2019	CCHF BY PCR	KPK	Peshawar	NEGATIVE
1904	2019	CCHF BY PCR	Baluchistan	Quetta	NEGATIVE
1905	2019	CCHF BY PCR	PUNJAB	MULTAN	NEGATIVE

1906	2019	CCHF BY PCR	SINDH	Karachi	NEGATIVE
1907	2019	CCHF BY PCR	Baluchistan	Quetta	NEGATIVE
1908	2019	CCHF BY PCR	KPK	Peshawar	NEGATIVE
1909	2019	CCHF BY PCR	SINDH	Karachi	NEGATIVE
1910	2019	CCHF BY PCR	SINDH	Karachi	NEGATIVE
1911	2019	CCHF BY PCR	SINDH	Karachi	NEGATIVE
1912	2019	CCHF BY PCR	SINDH	Karachi	NEGATIVE
1913	2019	CCHF BY PCR	SINDH	Karachi	NEGATIVE
1914	2019	CCHF BY PCR	Baluchistan	Quetta	POSITIVE
1915	2019	CCHF BY PCR	Baluchistan	Quetta	NEGATIVE
1916	2019	CCHF BY PCR	KPK	Peshawar	POSITIVE
1917	2019	CCHF BY PCR	KPK	Peshawar	POSITIVE
1918	2019	CCHF BY PCR	Baluchistan	Quetta	POSITIVE
1919	2019	CCHF BY PCR	KPK	Peshawar	NEGATIVE
1920	2019	CCHF BY PCR	KPK	Peshawar	NEGATIVE
1921	2019	CCHF BY PCR	KPK	Peshawar	NEGATIVE
1922	2019	CCHF BY PCR	KPK	Peshawar	NEGATIVE
1923	2019	CCHF BY PCR	KPK	Peshawar	NEGATIVE
1924	2019	CCHF BY PCR	KPK	Peshawar	NEGATIVE
1925	2019	CCHF BY PCR	KPK	Peshawar	NEGATIVE
1926	2019	CCHF BY PCR	KPK	Peshawar	NEGATIVE
1927	2019	CCHF BY PCR	KPK	Peshawar	NEGATIVE
1928	2019	CCHF BY PCR	KPK	Peshawar	NEGATIVE
1929	2019	CCHF BY PCR	KPK	Peshawar	NEGATIVE
1930	2019	CCHF BY PCR	KPK	Peshawar	NEGATIVE
1931	2019	CCHF BY PCR	KPK	Peshawar	NEGATIVE
1932	2019	CCHF BY PCR	KPK	Peshawar	NEGATIVE
1933	2019	CCHF BY PCR	KPK	Peshawar	NEGATIVE
1934	2019	CCHF BY PCR	KPK	Peshawar	NEGATIVE
1935	2019	CCHF BY PCR	KPK	Peshawar	NEGATIVE
1936	2019	CCHF BY PCR	KPK	Peshawar	NEGATIVE
1937	2019	CCHF BY PCR	KPK	Peshawar	NEGATIVE
1938	2019	CCHF BY PCR	KPK	Peshawar	NEGATIVE
1939	2019	CCHF BY PCR	SINDH	Karachi	POSITIVE
1940	2019	CCHF BY PCR	SINDH	Karachi	POSITIVE
1941	2019	CCHF BY PCR	SINDH	Karachi	NEGATIVE
1942	2019	CCHF BY PCR	SINDH	Karachi	POSITIVE
1943	2019	CCHF BY PCR	SINDH	Karachi	NEGATIVE
1944	2019	CCHF BY PCR	PUNJAB	Lahore	NEGATIVE
1945	2019	CCHF BY PCR	SINDH	Karachi	NEGATIVE
1946	2019	CCHF BY PCR	SINDH	Karachi	NEGATIVE
1947	2019	CCHF BY PCR	SINDH	Karachi	POSITIVE

1948	2019	CCHF BY PCR	Baluchistan	Quetta	NEGATIVE
1949	2019	CCHF BY PCR	Baluchistan	Quetta	NEGATIVE
1950	2019	CCHF BY PCR	Baluchistan	Quetta	NEGATIVE
1951	2019	CCHF BY PCR	SINDH	Karachi	NEGATIVE
1952	2019	CCHF BY PCR	Baluchistan	Quetta	NEGATIVE
1953	2019	CCHF BY PCR	Baluchistan	Quetta	POSITIVE
1954	2019	CCHF BY PCR	Baluchistan	Quetta	POSITIVE
1955	2019	CCHF BY PCR	SINDH	Karachi	NEGATIVE
1956	2019	CCHF BY PCR	SINDH	Karachi	NEGATIVE
1957	2019	CCHF BY PCR	SINDH	Karachi	NEGATIVE
1958	2019	CCHF BY PCR	Baluchistan	Quetta	POSITIVE
1959	2019	CCHF BY PCR	Baluchistan	Quetta	NEGATIVE
1960	2019	CCHF BY PCR	Baluchistan	Quetta	NEGATIVE
1961	2019	CCHF BY PCR	Baluchistan	Quetta	POSITIVE
1962	2019	CCHF BY PCR	PUNJAB	Lahore	NEGATIVE
1963	2019	CCHF BY PCR	SINDH	Karachi	NEGATIVE
1964	2019	CCHF BY PCR	KPK	Peshawar	POSITIVE
1965	2019	CCHF BY PCR	KPK	Peshawar	POSITIVE
1966	2019	CCHF BY PCR	Baluchistan	Quetta	POSITIVE
1967	2019	CCHF BY PCR	Baluchistan	Quetta	POSITIVE
1968	2019	CCHF BY PCR	KPK	Peshawar	NEGATIVE
1969	2019	CCHF BY PCR	KPK	Peshawar	POSITIVE
1970	2019	CCHF BY PCR	KPK	Timargarh	NEGATIVE
1971	2019	CCHF BY PCR	Baluchistan	Quetta	POSITIVE
1972	2019	CCHF BY PCR	Baluchistan	Quetta	POSITIVE
1973	2019	CCHF BY PCR	Baluchistan	Quetta	POSITIVE
1974	2019	CCHF BY PCR	KPK	Peshawar	NEGATIVE
1975	2019	CCHF BY PCR	Baluchistan	Quetta	POSITIVE
1976	2019	CCHF BY PCR	SINDH	Karachi	NEGATIVE
1977	2019	CCHF BY PCR	SINDH	Karachi	NEGATIVE
1978	2019	CCHF BY PCR	KPK	Peshawar	NEGATIVE
1979	2019	CCHF BY PCR	KPK	Haripur	NEGATIVE
1980	2019	CCHF BY PCR	Baluchistan	Quetta	POSITIVE

1981	2019	CCHF BY PCR	SINDH	Karachi	NEGATIVE
1982	2019	CCHF BY PCR	SINDH	Karachi	NEGATIVE
1983	2019	CCHF BY PCR	Baluchistan	Quetta	POSITIVE
1984	2019	CCHF BY PCR	KPK	Peshawar	POSITIVE
1985	2019	CCHF BY PCR	SINDH	Karachi	NEGATIVE
1986	2019	CCHF BY PCR	SINDH	Karachi	NEGATIVE
1987	2019	CCHF BY PCR	SINDH	Karachi	POSITIVE
1988	2019	CCHF BY PCR	SINDH	Karachi	POSITIVE
1989	2019	CCHF BY PCR	SINDH	Karachi	NEGATIVE
1990	2019	CCHF BY PCR	Baluchistan	Quetta	POSITIVE
1991	2019	CCHF BY PCR	SINDH	Karachi	NEGATIVE
1992	2019	CCHF BY PCR	KPK	Peshawar	NEGATIVE
1993	2019	CCHF BY PCR	PUNJAB	Lahore	NEGATIVE
1994	2019	CCHF BY PCR	SINDH	Karachi	NEGATIVE
1995	2019	CCHF BY PCR	SINDH	Karachi	NEGATIVE
1996	2019	CCHF BY PCR	Baluchistan	Quetta	NEGATIVE
1997	2019	CCHF BY PCR	SINDH	Karachi	NEGATIVE
1998	2019	CCHF BY PCR	SINDH	Karachi	POSITIVE
1999	2019	CCHF BY PCR	SINDH	Karachi	NEGATIVE
2000	2019	CCHF BY PCR	SINDH	Karachi	NEGATIVE
2001	2019	CCHF BY PCR	KPK	Peshawar	NEGATIVE
2002	2019	CCHF BY PCR	Baluchistan	Quetta	NEGATIVE
2003	2019	CCHF BY PCR	Baluchistan	Quetta	NEGATIVE
2004	2019	CCHF BY PCR	SINDH	Karachi	POSITIVE
2005	2019	CCHF BY PCR	SINDH	Karachi	NEGATIVE
2006	2019	CCHF BY PCR	SINDH	Karachi	NEGATIVE
2007	2019	CCHF BY PCR	Baluchistan	Quetta	POSITIVE
2008	2019	CCHF BY PCR	SINDH	Karachi	POSITIVE
2009	2019	CCHF BY PCR	KPK	Peshawar	NEGATIVE
2010	2019	CCHF BY PCR	KPK	Peshawar	NEGATIVE
2011	2019	CCHF BY PCR	KPK	Peshawar	NEGATIVE
2012	2019	CCHF BY PCR	SINDH	Karachi	NEGATIVE
2013	2019	CCHF BY PCR	Baluchistan	Quetta	NEGATIVE
2014	2019	CCHF BY PCR	KPK	Peshawar	NEGATIVE
2015	2019	CCHF BY PCR	SINDH	Karachi	POSITIVE
2016	2019	CCHF BY PCR	KPK	Peshawar	NEGATIVE
2017	2019	CCHF BY PCR	SINDH	Karachi	NEGATIVE
2018	2019	CCHF BY PCR	PUNJAB	Rawalpindi	NEGATIVE
2019	2019	CCHF BY PCR	SINDH	Karachi	NEGATIVE
2020	2019	CCHF BY PCR	SINDH	Karachi	POSITIVE
2021	2019	CCHF BY PCR	KPK	Peshawar	NEGATIVE

2022	2019	CCHF BY PCR	SINDH	Karachi	NEGATIVE
2023	2019	CCHF BY PCR	KPK	Peshawar	NEGATIVE
2024	2019	CCHF BY PCR	SINDH	Karachi	NEGATIVE
2025	2019	CCHF BY PCR	KPK	Peshawar	NEGATIVE
2026	2019	CCHF BY PCR	Baluchistan	Quetta	POSITIVE
2027	2019	CCHF BY PCR	KPK	Peshawar	POSITIVE
2028	2019	CCHF BY PCR	Baluchistan	Quetta	POSITIVE
2029	2019	CCHF BY PCR	SINDH	Karachi	NEGATIVE
2030	2019	CCHF BY PCR	KPK	Peshawar	POSITIVE
2031	2019	CCHF BY PCR	KPK	Peshawar	POSITIVE
2032	2019	CCHF BY PCR	SINDH	Karachi	NEGATIVE
2033	2019	CCHF BY PCR	KPK	Peshawar	POSITIVE
2034	2019	CCHF BY PCR	Baluchistan	Quetta	NEGATIVE
2035	2019	CCHF BY PCR	KPK	Peshawar	POSITIVE
2036	2019	CCHF BY PCR	SINDH	Karachi	NEGATIVE
2037	2019	CCHF BY PCR	SINDH	Karachi	NEGATIVE
2038	2019	CCHF BY PCR	KPK	Peshawar	NEGATIVE
2039	2019	CCHF BY PCR	SINDH	Karachi	NEGATIVE
2040	2019	CCHF BY PCR	KPK	Peshawar	NEGATIVE
2041	2019	CCHF BY PCR	PUNJAB	Mianwali	NEGATIVE
2042	2019	CCHF BY PCR	KPK	Peshawar	NEGATIVE
2043	2019	CCHF BY PCR	SINDH	Karachi	NEGATIVE
2044	2019	CCHF BY PCR	SINDH	Karachi	NEGATIVE
2045	2019	CCHF BY PCR	Baluchistan	Quetta	NEGATIVE
2046	2019	CCHF BY PCR	SINDH	Karachi	NEGATIVE
2047	2019	CCHF BY PCR	SINDH	Karachi	NEGATIVE
2048	2019	CCHF BY PCR	KPK	Peshawar	NEGATIVE
2049	2019	CCHF BY PCR	KPK	Peshawar	NEGATIVE
2050	2019	CCHF BY PCR	SINDH	Karachi	POSITIVE
2051	2019	CCHF BY PCR	KPK	Peshawar	NEGATIVE
2052	2019	CCHF BY PCR	SINDH	Karachi	NEGATIVE
2053	2019	CCHF BY PCR	KPK	Peshawar	NEGATIVE
2054	2019	CCHF BY PCR	PUNJAB	Sialkot	NEGATIVE
2055	2019	CCHF BY PCR	SINDH	Karachi	NEGATIVE
2056	2019	CCHF BY PCR	SINDH	Karachi	NEGATIVE
2057	2019	CCHF BY PCR	SINDH	Karachi	NEGATIVE
2058	2019	CCHF BY PCR	SINDH	Karachi	POSITIVE
2059	2019	CCHF BY PCR	KPK	Peshawar	POSITIVE
2060	2019	CCHF BY PCR	KPK	Peshawar	NEGATIVE
2061	2019	CCHF BY PCR	SINDH	Karachi	NEGATIVE
2062	2019	CCHF BY PCR	Baluchistan	Quetta	POSITIVE
2063	2019	CCHF BY PCR	PUNJAB	Mianwali	NEGATIVE

2064	2019	CCHF BY PCR	PUNJAB	Lahore	NEGATIVE
2065	2019	CCHF BY PCR	SINDH	Karachi	NEGATIVE
2066	2019	CCHF BY PCR	SINDH	Karachi	NEGATIVE
2067	2019	CCHF BY PCR	SINDH	Karachi	NEGATIVE
2068	2019	CCHF BY PCR	SINDH	Karachi	NEGATIVE
2069	2019	CCHF BY PCR	KPK	Peshawar	POSITIVE
2070	2019	CCHF BY PCR	KPK	Peshawar	NEGATIVE
2071	2019	CCHF BY PCR	SINDH	Karachi	POSITIVE
2072	2019	CCHF BY PCR	Baluchistan	Quetta	POSITIVE
2073	2019	CCHF BY PCR	SINDH	Karachi	NEGATIVE
2074	2019	CCHF BY PCR	KPK	Mansehra	NEGATIVE
2075	2019	CCHF BY PCR	SINDH	Karachi	NEGATIVE
2076	2019	CCHF BY PCR	KPK	Peshawar	POSITIVE
2077	2019	CCHF BY PCR	KPK	Peshawar	NEGATIVE
2078	2019	CCHF BY PCR	SINDH	Karachi	NEGATIVE
2079	2019	CCHF BY PCR	SINDH	Karachi	NEGATIVE
2080	2019	CCHF BY PCR	KPK	Peshawar	POSITIVE
2081	2019	CCHF BY PCR	SINDH	Karachi	NEGATIVE
2082	2019	CCHF BY PCR	SINDH	Hyderabad	POSITIVE
2083	2019	CCHF BY PCR	SINDH	Karachi	NEGATIVE
2084	2019	CCHF BY PCR	KPK	Peshawar	NEGATIVE
2085	2019	CCHF BY PCR	SINDH	Karachi	NEGATIVE
2086	2019	CCHF BY PCR	KPK	Peshawar	NEGATIVE
2087	2019	CCHF BY PCR	SINDH	Hyderabad	NEGATIVE
2088	2019	CCHF BY PCR	KPK	Peshawar	POSITIVE
2089	2019	CCHF BY PCR	KPK	Peshawar	POSITIVE
2090	2019	CCHF BY PCR	KPK	Peshawar	POSITIVE
2091	2019	CCHF BY PCR	KPK	Peshawar	NEGATIVE
2092	2019	CCHF BY PCR	Baluchistan	Quetta	NEGATIVE
2093	2019	CCHF BY PCR	SINDH	Karachi	NEGATIVE
2094	2019	CCHF BY PCR	KPK	Peshawar	NEGATIVE
2095	2019	CCHF BY PCR	SINDH	Karachi	NEGATIVE
2096	2019	CCHF BY PCR	SINDH	Karachi	NEGATIVE
2097	2019	CCHF BY PCR	SINDH	Karachi	NEGATIVE
2098	2019	CCHF BY PCR	KPK	Dera Ismail Khan	NEGATIVE
2099	2019	CCHF BY PCR	SINDH	Karachi	NEGATIVE
2100	2019	CCHF BY PCR	SINDH	Karachi	NEGATIVE
2101	2019	CCHF BY PCR	SINDH	Karachi	NEGATIVE
2102	2019	CCHF BY PCR	PUNJAB	Sadiqabad	NEGATIVE
2103	2019	CCHF BY PCR	SINDH	Karachi	NEGATIVE
2104	2019	CCHF BY PCR	SINDH	Karachi	NEGATIVE
2105	2019	CCHF BY PCR	SINDH	Karachi	NEGATIVE
2106	2019	CCHF BY PCR	KPK	Peshawar	NEGATIVE
2107	2019	CCHF BY PCR	KPK	Peshawar	NEGATIVE

2108	2019	CCHF BY PCR	SINDH	Karachi	NEGATIVE
2109	2019	CCHF BY PCR	SINDH	Hyderabad	POSITIVE
2110	2019	CCHF BY PCR	SINDH	Karachi	NEGATIVE
2111	2019	CCHF BY PCR	SINDH	Karachi	NEGATIVE
2112	2019	CCHF BY PCR	KPK	Peshawar	NEGATIVE
2113	2019	CCHF BY PCR	SINDH	Karachi	NEGATIVE
2114	2019	CCHF BY PCR	SINDH	Karachi	NEGATIVE
2115	2019	CCHF BY PCR	SINDH	Karachi	NEGATIVE
2116	2019	CCHF BY PCR	SINDH	Karachi	NEGATIVE
2117	2019	CCHF BY PCR	Baluchistan	Quetta	NEGATIVE
2118	2019	CCHF BY PCR	SINDH	Karachi	NEGATIVE
2119	2019	CCHF BY PCR	SINDH	Karachi	NEGATIVE
2120	2019	CCHF BY PCR	SINDH	Karachi	NEGATIVE
2121	2019	CCHF BY PCR	KPK	Peshawar	NEGATIVE
2122	2019	CCHF BY PCR	Baluchistan	Quetta	NEGATIVE
2123	2019	CCHF BY PCR	KPK	Peshawar	POSITIVE
2124	2019	CCHF BY PCR	SINDH	Karachi	NEGATIVE
2125	2019	CCHF BY PCR	SINDH	Karachi	NEGATIVE
2126	2019	CCHF BY PCR	PUNJAB	Islamabad	NEGATIVE
2127	2019	CCHF BY PCR	PUNJAB	Lahore	NEGATIVE
2128	2019	CCHF BY PCR	SINDH	Hyderabad	NEGATIVE
2129	2019	CCHF BY PCR	SINDH	Karachi	NEGATIVE
2130	2019	CCHF BY PCR	KPK	Peshawar	NEGATIVE
2131	2019	CCHF BY PCR	SINDH	Karachi	NEGATIVE
2132	2019	CCHF BY PCR	SINDH	Karachi	NEGATIVE
2133	2019	CCHF BY PCR	SINDH	Karachi	NEGATIVE
2134	2019	CCHF BY PCR	SINDH	Karachi	POSITIVE
2135	2019	CCHF BY PCR	SINDH	Karachi	NEGATIVE
2136	2019	CCHF BY PCR	SINDH	Karachi	NEGATIVE
2137	2019	CCHF BY PCR	SINDH	Karachi	NEGATIVE
2138	2019	CCHF BY PCR	SINDH	Umerkot	NEGATIVE
2139	2019	CCHF BY PCR	SINDH	Umerkot	NEGATIVE
2140	2019	CCHF BY PCR	SINDH	Umerkot	NEGATIVE
2141	2019	CCHF BY PCR	SINDH	Umerkot	NEGATIVE
2142	2019	CCHF BY PCR	SINDH	Umerkot	NEGATIVE
2143	2019	CCHF BY PCR	SINDH	Umerkot	NEGATIVE
2144	2019	CCHF BY PCR	SINDH	Umerkot	NEGATIVE
2145	2019	CCHF BY PCR	SINDH	Umerkot	NEGATIVE
2146	2019	CCHF BY PCR	SINDH	Umerkot	NEGATIVE
2147	2019	CCHF BY PCR	SINDH	Umerkot	NEGATIVE
2148	2019	CCHF BY PCR	SINDH	Umerkot	NEGATIVE
2149	2019	CCHF BY PCR	SINDH	Umerkot	NEGATIVE
2150	2019	CCHF BY PCR	SINDH	Umerkot	NEGATIVE
2151	2019	CCHF BY PCR	SINDH	Umerkot	NEGATIVE

2152	2019	CCHF BY PCR	SINDH	Umerkot	NEGATIVE
2153	2019	CCHF BY PCR	SINDH	Umerkot	NEGATIVE
2154	2019	CCHF BY PCR	SINDH	Karachi	NEGATIVE
2155	2019	CCHF BY PCR	SINDH	Karachi	NEGATIVE
2156	2019	CCHF BY PCR	SINDH	Karachi	NEGATIVE
2157	2019	CCHF BY PCR	SINDH	Karachi	NEGATIVE
2158	2019	CCHF BY PCR	SINDH	Karachi	NEGATIVE
2159	2019	CCHF BY PCR	SINDH	Karachi	NEGATIVE
2160	2019	CCHF BY PCR	KPK	Peshawar	NEGATIVE
2161	2019	CCHF BY PCR	KPK	Peshawar	NEGATIVE
2162	2019	CCHF BY PCR	SINDH	Karachi	NEGATIVE
2163	2019	CCHF BY PCR	Baluchistan	Quetta	NEGATIVE
2164	2019	CCHF BY PCR	SINDH	Karachi	NEGATIVE
2165	2019	CCHF BY PCR	SINDH	Karachi	NEGATIVE
2166	2019	CCHF BY PCR	Baluchistan	Quetta	NEGATIVE
2167	2019	CCHF BY PCR	Baluchistan	Quetta	NEGATIVE
2168	2020	CCHF BY PCR	Baluchistan	Quetta	NEGATIVE
2169	2020	CCHF BY PCR	SINDH	Karachi	NEGATIVE
2170	2020	CCHF BY PCR	KPK	Peshawar	NEGATIVE
2171	2020	CCHF BY PCR	SINDH	Karachi	NEGATIVE
2172	2020	CCHF BY PCR	SINDH	Karachi	NEGATIVE
2173	2020	CCHF BY PCR	SINDH	Karachi	NEGATIVE
2174	2020	CCHF BY PCR	KPK	Peshawar	NEGATIVE
2175	2020	CCHF BY PCR	SINDH	Karachi	NEGATIVE
2176	2020	CCHF BY PCR	SINDH	Karachi	NEGATIVE
2177	2020	CCHF BY PCR	Baluchistan	Quetta	NEGATIVE
2178	2020	CCHF BY PCR	SINDH	Karachi	NEGATIVE
2179	2020	CCHF BY PCR	SINDH	Karachi	NEGATIVE
2180	2020	CCHF BY PCR	SINDH	Karachi	NEGATIVE
2181	2020	CCHF BY PCR	Baluchistan	Quetta	NEGATIVE
2182	2020	CCHF BY PCR	Baluchistan	Quetta	NEGATIVE
2183	2020	CCHF BY PCR	SINDH	Karachi	POSITIVE
2184	2020	CCHF BY PCR	SINDH	Karachi	NEGATIVE
2185	2020	CCHF BY PCR	SINDH	Karachi	NEGATIVE
2186	2020	CCHF BY PCR	SINDH	Karachi	POSITIVE
2187	2020	CCHF BY PCR	SINDH	Karachi	NEGATIVE
2188	2020	CCHF BY PCR	SINDH	Mirpurkhas	NEGATIVE
2189	2020	CCHF BY PCR	SINDH	Mirpurkhas	NEGATIVE
2190	2020	CCHF BY PCR	SINDH	Mirpurkhas	NEGATIVE
2191	2020	CCHF BY PCR	SINDH	Mirpurkhas	NEGATIVE
2192	2020	CCHF BY PCR	SINDH	Mirpurkhas	NEGATIVE

2193	2020	CCHF BY PCR	SINDH	Mirpurkhas	NEGATIVE
2194	2020	CCHF BY PCR	SINDH	Mirpurkhas	NEGATIVE
2195	2020	CCHF BY PCR	PUNJAB	RAHIM YAR KHAN	NEGATIVE
2196	2020	CCHF BY PCR	SINDH	Karachi	NEGATIVE
2197	2020	CCHF BY PCR	Baluchistan	Quetta	NEGATIVE
2198	2020	CCHF BY PCR	Baluchistan	Quetta	NEGATIVE
2199	2020	CCHF BY PCR	KPK	Peshawar	NEGATIVE
2200	2020	CCHF BY PCR	Baluchistan	Quetta	NEGATIVE
2201	2020	CCHF BY PCR	Baluchistan	Quetta	NEGATIVE
2202	2020	CCHF BY PCR	Baluchistan	Quetta	NEGATIVE
2203	2020	CCHF BY PCR	KPK	Peshawar	POSITIVE
2204	2020	CCHF BY PCR	SINDH	Karachi	NEGATIVE
2205	2020	CCHF BY PCR	SINDH	Karachi	POSITIVE
2206	2020	CCHF BY PCR	SINDH	Karachi	NEGATIVE
2207	2020	CCHF BY PCR	SINDH	Karachi	NEGATIVE
2208	2020	CCHF BY PCR	SINDH	Karachi	NEGATIVE
2209	2020	CCHF BY PCR	SINDH	Karachi	NEGATIVE
2210	2020	CCHF BY PCR	SINDH	Karachi	POSITIVE
2211	2020	CCHF BY PCR	SINDH	Karachi	POSITIVE
2212	2020	CCHF BY PCR	SINDH	Karachi	NEGATIVE
2213	2020	CCHF BY PCR	SINDH	Karachi	NEGATIVE
2214	2020	CCHF BY PCR	SINDH	Karachi	POSITIVE
2215	2020	CCHF BY PCR	Baluchistan	Quetta	POSITIVE
2216	2020	CCHF BY PCR	SINDH	Karachi	NEGATIVE
2217	2020	CCHF BY PCR	SINDH	Karachi	NEGATIVE
2218	2020	CCHF BY PCR	SINDH	Karachi	NEGATIVE
2219	2020	CCHF BY PCR	SINDH	Karachi	NEGATIVE
2220	2020	CCHF BY PCR	Baluchistan	Quetta	POSITIVE
2221	2020	CCHF BY PCR	SINDH	Karachi	NEGATIVE
2222	2020	CCHF BY PCR	SINDH	Karachi	POSITIVE
2223	2020	CCHF BY PCR	SINDH	Karachi	POSITIVE
2224	2020	CCHF BY PCR	SINDH	Karachi	NEGATIVE
2225	2020	CCHF BY PCR	SINDH	Karachi	NEGATIVE
2226	2020	CCHF BY PCR	Baluchistan	Quetta	POSITIVE
2227	2020	CCHF BY PCR	SINDH	Karachi	POSITIVE
2228	2020	CCHF BY PCR	KPK	Peshawar	POSITIVE
2229	2020	CCHF BY PCR	SINDH	Karachi	NEGATIVE
2230	2020	CCHF BY PCR	SINDH	Karachi	NEGATIVE

2231	2020	CCHF BY PCR	Baluchistan	Quetta	NEGATIVE
2232	2020	CCHF BY PCR	KPK	Peshawar	NEGATIVE
2233	2020	CCHF BY PCR	Baluchistan	Quetta	NEGATIVE
2234	2020	CCHF BY PCR	SINDH	Karachi	POSITIVE
2235	2020	CCHF BY PCR	SINDH	Karachi	NEGATIVE
2236	2020	CCHF BY PCR	SINDH	Karachi	NEGATIVE
2237	2020	CCHF BY PCR	Baluchistan	Quetta	POSITIVE
2238	2020	CCHF BY PCR	SINDH	Karachi	NEGATIVE
2239	2020	CCHF BY PCR	Baluchistan	Quetta	NEGATIVE
2240	2020	CCHF BY PCR	Baluchistan	Quetta	POSITIVE
2241	2020	CCHF BY PCR	KPK	Peshawar	NEGATIVE
2242	2020	CCHF BY PCR	Baluchistan	Quetta	NEGATIVE
2243	2020	CCHF BY PCR	SINDH	Karachi	NEGATIVE
2244	2020	CCHF BY PCR	Baluchistan	Quetta	POSITIVE
2245	2020	CCHF BY PCR	Baluchistan	Quetta	POSITIVE
2246	2020	CCHF BY PCR	Baluchistan	Quetta	POSITIVE
2247	2020	CCHF BY PCR	SINDH	Karachi	NEGATIVE
2248	2020	CCHF BY PCR	Baluchistan	Quetta	POSITIVE
2249	2020	CCHF BY PCR	SINDH	Karachi	POSITIVE
2250	2020	CCHF BY PCR	Baluchistan	Quetta	POSITIVE
2251	2020	CCHF BY PCR	KPK	Peshawar	NEGATIVE
2252	2020	CCHF BY PCR	SINDH	Karachi	NEGATIVE
2253	2020	CCHF BY PCR	SINDH	Karachi	NEGATIVE
2254	2020	CCHF BY PCR	SINDH	Karachi	NEGATIVE
2255	2020	CCHF BY PCR	Baluchistan	Quetta	POSITIVE
2256	2020	CCHF BY PCR	SINDH	Karachi	NEGATIVE
2257	2020	CCHF BY PCR	SINDH	Karachi	NEGATIVE
2258	2020	CCHF BY PCR	SINDH	Karachi	NEGATIVE
2259	2020	CCHF BY PCR	SINDH	Karachi	NEGATIVE
2260	2020	CCHF BY PCR	SINDH	Karachi	NEGATIVE
2261	2020	CCHF BY PCR	Baluchistan	Quetta	NEGATIVE
2262	2020	CCHF BY PCR	Baluchistan	Quetta	NEGATIVE
2263	2020	CCHF BY PCR	SINDH	Karachi	NEGATIVE
2264	2020	CCHF BY PCR	SINDH	Karachi	NEGATIVE
2265	2020	CCHF BY PCR	SINDH	Karachi	NEGATIVE

2266	2020	CCHF BY PCR	Baluchistan	Quetta	NEGATIVE
2267	2020	CCHF BY PCR	SINDH	Karachi	NEGATIVE
2268	2020	CCHF BY PCR	PUNJAB	Islamabad	NEGATIVE
2269	2020	CCHF BY PCR	SINDH	Karachi	NEGATIVE
2270	2020	CCHF BY PCR	Baluchistan	Quetta	NEGATIVE
2271	2020	CCHF BY PCR	PUNJAB	Lahore	NEGATIVE
2272	2020	CCHF BY PCR	SINDH	Karachi	POSITIVE
2273	2020	CCHF BY PCR	Baluchistan	Quetta	POSITIVE
2274	2020	CCHF BY PCR	SINDH	Karachi	NEGATIVE
2275	2020	CCHF BY PCR	SINDH	Karachi	POSITIVE
2276	2020	CCHF BY PCR	KPK	Peshawar	POSITIVE
2277	2021	CCHF BY PCR	PUNJAB	Islamabad	NEGATIVE
2278	2021	CCHF BY PCR	KPK	Peshawar	NEGATIVE
2279	2021	CCHF BY PCR	SINDH	Karachi	NEGATIVE
2280	2021	CCHF BY PCR	Baluchistan	Quetta	NEGATIVE
2281	2021	CCHF BY PCR	SINDH	Karachi	POSITIVE
2282	2021	CCHF BY PCR	SINDH	Karachi	NEGATIVE
2283	2021	CCHF BY PCR	SINDH	Karachi	NEGATIVE
2284	2021	CCHF BY PCR	SINDH	Karachi	NEGATIVE
2285	2021	CCHF BY PCR	SINDH	Karachi	NEGATIVE
2286	2021	CCHF BY PCR	SINDH	Karachi	NEGATIVE
2287	2021	CCHF BY PCR	KPK	Peshawar	POSITIVE
2288	2021	CCHF BY PCR	SINDH	Karachi	NEGATIVE
2289	2021	CCHF BY PCR	KPK	Peshawar	POSITIVE
2290	2021	CCHF BY PCR	SINDH	Karachi	POSITIVE
2291	2021	CCHF BY PCR	Baluchistan	Quetta	NEGATIVE
2292	2021	CCHF BY PCR	Baluchistan	Quetta	NEGATIVE
2293	2021	CCHF BY PCR	SINDH	Karachi	NEGATIVE
2294	2021	CCHF BY PCR	SINDH	Karachi	NEGATIVE
2295	2021	CCHF BY PCR	SINDH	Karachi	NEGATIVE
2296	2021	CCHF BY PCR	SINDH	Karachi	NEGATIVE
2297	2021	CCHF BY PCR	Baluchistan	Quetta	NEGATIVE
2298	2021	CCHF BY PCR	Baluchistan	Quetta	POSITIVE
2299	2021	CCHF BY PCR	SINDH	Karachi	NEGATIVE
2300	2021	CCHF BY PCR	SINDH	Karachi	NEGATIVE
2301	2021	CCHF BY PCR	PUNJAB	Islamabad	NEGATIVE
2302	2021	CCHF BY PCR	Baluchistan	Quetta	POSITIVE
2303	2021	CCHF BY PCR	PUNJAB	RAHIM YAR KHAN	NEGATIVE

2304	2021	CCHF BY PCR	SINDH	Karachi	NEGATIVE
2305	2021	CCHF BY PCR	PUNJAB	Lahore	NEGATIVE
2306	2021	CCHF BY PCR	SINDH	Karachi	NEGATIVE
2307	2021	CCHF BY PCR	PUNJAB	MULTAN	NEGATIVE
2308	2021	CCHF BY PCR	KPK	Peshawar	POSITIVE
2309	2021	CCHF BY PCR	SINDH	Karachi	NEGATIVE
2310	2021	CCHF BY PCR	KPK	Peshawar	POSITIVE
2311	2021	CCHF BY PCR	SINDH	Karachi	NEGATIVE
2312	2021	CCHF BY PCR	SINDH	Karachi	NEGATIVE
2313	2021	CCHF BY PCR	SINDH	Karachi	NEGATIVE
2314	2021	CCHF BY PCR	SINDH	Hyderabad	NEGATIVE
2315	2021	CCHF BY PCR	KPK	Peshawar	POSITIVE
2316	2021	CCHF BY PCR	SINDH	Karachi	NEGATIVE
2317	2021	CCHF BY PCR	KPK	Peshawar	NEGATIVE
2318	2021	CCHF BY PCR	SINDH	Karachi	NEGATIVE
2319	2021	CCHF BY PCR	SINDH	Karachi	NEGATIVE
2320	2021	CCHF BY PCR	KPK	Peshawar	NEGATIVE
2321	2021	CCHF BY PCR	KPK	Peshawar	POSITIVE
2322	2021	CCHF BY PCR	Baluchistan	Quetta	POSITIVE
2323	2021	CCHF BY PCR	SINDH	Karachi	POSITIVE
2324	2021	CCHF BY PCR	SINDH	Karachi	NEGATIVE
2325	2021	CCHF BY PCR	KPK	Peshawar	NEGATIVE
2326	2021	CCHF BY PCR	PUNJAB	KHANEWAL	NEGATIVE
2327	2021	CCHF BY PCR	SINDH	Karachi	NEGATIVE
2328	2021	CCHF BY PCR	SINDH	Karachi	NEGATIVE
2329	2021	CCHF BY PCR	KPK	Peshawar	NEGATIVE
2330	2021	CCHF BY PCR	KPK	Peshawar	NEGATIVE
2331	2021	CCHF BY PCR	KPK	Peshawar	NEGATIVE
2332	2021	CCHF BY PCR	Baluchistan	Quetta	NEGATIVE
2333	2021	CCHF BY PCR	KPK	Peshawar	NEGATIVE
2334	2021	CCHF BY PCR	PUNJAB	Lahore	NEGATIVE
2335	2021	CCHF BY PCR	Baluchistan	Quetta	POSITIVE
2336	2021	CCHF BY PCR	SINDH	Karachi	NEGATIVE
2337	2021	CCHF BY PCR	SINDH	Karachi	NEGATIVE
2338	2021	CCHF BY PCR	KPK	Peshawar	POSITIVE
2339	2021	CCHF BY PCR	KPK	Peshawar	POSITIVE
2340	2021	CCHF BY PCR	Baluchistan	Quetta	NEGATIVE
2341	2021	CCHF BY PCR	Baluchistan	Quetta	NEGATIVE
2342	2021	CCHF BY PCR	SINDH	Karachi	POSITIVE
2343	2021	CCHF BY PCR	SINDH	Karachi	NEGATIVE
2344	2021	CCHF BY PCR	SINDH	Karachi	NEGATIVE
2345	2021	CCHF BY PCR	KPK	Peshawar	POSITIVE

2346	2021	CCHF BY PCR	KPK	Peshawar	NEGATIVE
2347	2021	CCHF BY PCR	SINDH	Karachi	NEGATIVE
2348	2021	CCHF BY PCR	KPK	Peshawar	NEGATIVE
2349	2021	CCHF BY PCR	KPK	Peshawar	NEGATIVE
2350	2021	CCHF BY PCR	SINDH	Karachi	POSITIVE
2351	2021	CCHF BY PCR	SINDH	Karachi	NEGATIVE
2352	2021	CCHF BY PCR	SINDH	Karachi	NEGATIVE
2353	2021	CCHF BY PCR	Baluchistan	Quetta	NEGATIVE
2354	2021	CCHF BY PCR	Baluchistan	Quetta	POSITIVE
2355	2021	CCHF BY PCR	KPK	Peshawar	NEGATIVE
2356	2021	CCHF BY PCR	Baluchistan	Quetta	NEGATIVE
2357	2021	CCHF BY PCR	KPK	Peshawar	NEGATIVE
2358	2021	CCHF BY PCR	SINDH	Karachi	NEGATIVE
2359	2021	CCHF BY PCR	KPK	Peshawar	POSITIVE
2360	2021	CCHF BY PCR	KPK	Peshawar	NEGATIVE
2361	2021	CCHF BY PCR	KPK	Peshawar	NEGATIVE
2362	2021	CCHF BY PCR	SINDH	Karachi	NEGATIVE
2363	2021	CCHF BY PCR	KPK	Peshawar	NEGATIVE
2364	2021	CCHF BY PCR	SINDH	Karachi	POSITIVE
2365	2021	CCHF BY PCR	SINDH	Karachi	NEGATIVE
2366	2021	CCHF BY PCR	SINDH	Karachi	NEGATIVE
2367	2021	CCHF BY PCR	SINDH	Karachi	NEGATIVE
2368	2021	CCHF BY PCR	SINDH	Karachi	NEGATIVE
2369	2021	CCHF BY PCR	SINDH	Karachi	NEGATIVE
2370	2021	CCHF BY PCR	SINDH	Karachi	NEGATIVE
2371	2021	CCHF BY PCR	PUNJAB	MULTAN	NEGATIVE
2372	2021	CCHF BY PCR	SINDH	Karachi	NEGATIVE
2373	2021	CCHF BY PCR	SINDH	Karachi	NEGATIVE
2374	2021	CCHF BY PCR	SINDH	Karachi	NEGATIVE
2375	2021	CCHF BY PCR	KPK	Peshawar	NEGATIVE
2376	2021	CCHF BY PCR	SINDH	Karachi	NEGATIVE
2377	2021	CCHF BY PCR	SINDH	Karachi	NEGATIVE
2378	2021	CCHF BY PCR	KPK	Peshawar	NEGATIVE
2379	2021	CCHF BY PCR	PUNJAB	Rawalpindi	NEGATIVE
2380	2021	CCHF BY PCR	SINDH	Karachi	NEGATIVE
2381	2021	CCHF BY PCR	SINDH	Karachi	POSITIVE
2382	2021	CCHF BY PCR	KPK	Peshawar	NEGATIVE
2383	2021	CCHF BY PCR	Baluchistan	Quetta	NEGATIVE
2384	2021	CCHF BY PCR	SINDH	Karachi	NEGATIVE
2385	2021	CCHF BY PCR	Baluchistan	Quetta	NEGATIVE
2386	2021	CCHF BY PCR	SINDH	Karachi	NEGATIVE
2387	2021	CCHF BY PCR	SINDH	Karachi	NEGATIVE

2388	2021	CCHF BY PCR	SINDH	Karachi	POSITIVE
2389	2021	CCHF BY PCR	Baluchistan	Quetta	NEGATIVE
2390	2022	CCHF BY PCR	SINDH	Karachi	NEGATIVE
2391	2022	CCHF BY PCR	SINDH	Karachi	NEGATIVE
2392	2022	CCHF BY PCR	SINDH	Karachi	NEGATIVE
2393	2022	CCHF BY PCR	Baluchistan	Quetta	NEGATIVE
2394	2022	CCHF BY PCR	Baluchistan	Quetta	NEGATIVE
2395	2022	CCHF BY PCR	SINDH	Karachi	NEGATIVE
2396	2022	CCHF BY PCR	SINDH	Karachi	NEGATIVE
2397	2022	CCHF BY PCR	SINDH	Karachi	NEGATIVE
2398	2022	CCHF BY PCR	SINDH	Karachi	NEGATIVE
2399	2022	CCHF BY PCR	Baluchistan	Quetta	NEGATIVE
2400	2022	CCHF BY PCR	KPK	Peshawar	NEGATIVE
2401	2022	CCHF BY PCR	SINDH	Karachi	NEGATIVE
2402	2022	CCHF BY PCR	SINDH	Karachi	POSITIVE
2403	2022	CCHF BY PCR	Baluchistan	Quetta	POSITIVE
2404	2022	CCHF BY PCR	SINDH	Karachi	NEGATIVE
2405	2022	CCHF BY PCR	SINDH	Karachi	NEGATIVE
2406	2022	CCHF BY PCR	SINDH	Karachi	NEGATIVE
2407	2022	CCHF BY PCR	KPK	Peshawar	NEGATIVE
2408	2022	CCHF BY PCR	KPK	Peshawar	NEGATIVE
2409	2022	CCHF BY PCR	Baluchistan	Quetta	NEGATIVE
2410	2022	CCHF BY PCR	SINDH	Karachi	POSITIVE
2411	2022	CCHF BY PCR	SINDH	Karachi	NEGATIVE
2412	2022	CCHF BY PCR	Baluchistan	Quetta	NEGATIVE
2413	2022	CCHF BY PCR	SINDH	Karachi	NEGATIVE
2414	2022	CCHF BY PCR	KPK	Peshawar	NEGATIVE
2415	2022	CCHF BY PCR	Baluchistan	Quetta	NEGATIVE
2416	2022	CCHF BY PCR	Baluchistan	Quetta	NEGATIVE
2417	2022	CCHF BY PCR	Baluchistan	Quetta	NEGATIVE
2418	2022	CCHF BY PCR	KPK	Peshawar	NEGATIVE
2419	2022	CCHF BY PCR	SINDH	Karachi	NEGATIVE
2420	2022	CCHF BY PCR	KPK	Peshawar	NEGATIVE
2421	2022	CCHF BY PCR	Baluchistan	Quetta	NEGATIVE
2422	2022	CCHF BY PCR	Baluchistan	Quetta	NEGATIVE
2423	2022	CCHF BY PCR	Baluchistan	Quetta	NEGATIVE

2424	2022	CCHF BY PCR	SINDH	Karachi	NEGATIVE
2425	2022	CCHF BY PCR	KPK	Peshawar	POSITIVE
2426	2022	CCHF BY PCR	Baluchistan	Quetta	POSITIVE
2427	2022	CCHF BY PCR	Baluchistan	Quetta	POSITIVE
2428	2022	CCHF BY PCR	KPK	Peshawar	NEGATIVE
2429	2022	CCHF BY PCR	Baluchistan	Quetta	NEGATIVE
2430	2022	CCHF BY PCR	Baluchistan	Quetta	NEGATIVE
2431	2022	CCHF BY PCR	Baluchistan	Quetta	POSITIVE
2432	2022	CCHF BY PCR	Baluchistan	Quetta	NEGATIVE
2433	2022	CCHF BY PCR	Baluchistan	Quetta	NEGATIVE
2434	2022	CCHF BY PCR	Baluchistan	Quetta	NEGATIVE
2435	2022	CCHF BY PCR	SINDH	Karachi	NEGATIVE
2436	2022	CCHF BY PCR	KPK	Peshawar	NEGATIVE
2437	2022	CCHF BY PCR	KPK	Peshawar	NEGATIVE
2438	2022	CCHF BY PCR	Baluchistan	Quetta	NEGATIVE
2439	2022	CCHF BY PCR	KPK	Peshawar	POSITIVE
2440	2022	CCHF BY PCR	KPK	Peshawar	NEGATIVE
2441	2022	CCHF BY PCR	Baluchistan	Quetta	POSITIVE
2442	2022	CCHF BY PCR	KPK	Peshawar	POSITIVE
2443	2022	CCHF BY PCR	KPK	Peshawar	POSITIVE
2444	2022	CCHF BY PCR	KPK	Peshawar	NEGATIVE
2445	2022	CCHF BY PCR	KPK	Peshawar	NEGATIVE
2446	2022	CCHF BY PCR	KPK	Peshawar	POSITIVE
2447	2022	CCHF BY PCR	SINDH	Karachi	NEGATIVE
2448	2022	CCHF BY PCR	SINDH	Karachi	NEGATIVE
2449	2022	CCHF BY PCR	Baluchistan	Quetta	POSITIVE
2450	2022	CCHF BY PCR	SINDH	Karachi	NEGATIVE
2451	2022	CCHF BY PCR	KPK	Peshawar	NEGATIVE
2452	2022	CCHF BY PCR	Baluchistan	Quetta	NEGATIVE
2453	2022	CCHF BY PCR	SINDH	Karachi	NEGATIVE
2454	2022	CCHF BY PCR	Baluchistan	Quetta	NEGATIVE
2455	2022	CCHF BY PCR	SINDH	Hyderabad	NEGATIVE
2456	2022	CCHF BY PCR	SINDH	Karachi	NEGATIVE
2457	2022	CCHF BY PCR	SINDH	Karachi	POSITIVE
2458	2022	CCHF BY PCR	SINDH	Karachi	POSITIVE
2459	2022	CCHF BY PCR	SINDH	Hyderabad	NEGATIVE

2460	2022	CCHF BY PCR	SINDH	Karachi	NEGATIVE
2461	2022	CCHF BY PCR	KPK	Peshawar	POSITIVE
2462	2022	CCHF BY PCR	SINDH	Hub	NEGATIVE
2463	2022	CCHF BY PCR	SINDH	Hub	NEGATIVE
2464	2022	CCHF BY PCR	SINDH	Hub	NEGATIVE
2465	2022	CCHF BY PCR	SINDH	Hub	NEGATIVE
2466	2022	CCHF BY PCR	SINDH	Hub	NEGATIVE
2467	2022	CCHF BY PCR	SINDH	Karachi	NEGATIVE
2468	2022	CCHF BY PCR	SINDH	Karachi	POSITIVE
2469	2022	CCHF BY PCR	SINDH	Karachi	NEGATIVE
2470	2022	CCHF BY PCR	SINDH	Karachi	POSITIVE
2471	2022	CCHF BY PCR	KPK	BANNU	POSITIVE
2472	2022	CCHF BY PCR	SINDH	Karachi	POSITIVE
2473	2022	CCHF BY PCR	SINDH	Karachi	POSITIVE
2474	2022	CCHF BY PCR	SINDH	Karachi	NEGATIVE
2475	2022	CCHF BY PCR	SINDH	Karachi	NEGATIVE
2476	2022	CCHF BY PCR	SINDH	Karachi	NEGATIVE
2477	2022	CCHF BY PCR	Baluchistan	Quetta	NEGATIVE
2478	2022	CCHF BY PCR	Baluchistan	Quetta	NEGATIVE
2479	2022	CCHF BY PCR	KPK	Peshawar	NEGATIVE
2480	2022	CCHF BY PCR	SINDH	Karachi	NEGATIVE
2481	2022	CCHF BY PCR	SINDH	Karachi	NEGATIVE
2482	2022	CCHF BY PCR	SINDH	Karachi	NEGATIVE
2483	2022	CCHF BY PCR	SINDH	Karachi	POSITIVE
2484	2022	CCHF BY PCR	SINDH	Karachi	NEGATIVE
2485	2022	CCHF BY PCR	SINDH	Karachi	NEGATIVE
2486	2022	CCHF BY PCR	KPK	BANNU	NEGATIVE
2487	2022	CCHF BY PCR	SINDH	Karachi	NEGATIVE
2488	2022	CCHF BY PCR	KPK	Peshawar	NEGATIVE
2489	2022	CCHF BY PCR	SINDH	Karachi	NEGATIVE
2490	2022	CCHF BY PCR	SINDH	Karachi	NEGATIVE
2491	2022	CCHF BY PCR	SINDH	Karachi	NEGATIVE
2492	2022	CCHF BY PCR	KPK	Peshawar	NEGATIVE
2493	2022	CCHF BY PCR	KPK	BANNU	NEGATIVE
2494	2022	CCHF BY PCR	SINDH	Karachi	NEGATIVE
2495	2022	CCHF BY PCR	Baluchistan	Quetta	NEGATIVE
2496	2022	CCHF BY PCR	KPK	Peshawar	NEGATIVE
2497	2022	CCHF BY PCR	Baluchistan	Quetta	NEGATIVE
2498	2022	CCHF BY PCR	KPK	Peshawar	NEGATIVE
2499	2022	CCHF BY PCR	SINDH	Karachi	NEGATIVE
2500	2022	CCHF BY PCR	SINDH	Karachi	NEGATIVE
2501	2022	CCHF BY PCR	KPK	Peshawar	NEGATIVE
2502	2022	CCHF BY PCR	SINDH	Karachi	NEGATIVE

2503	2022	CCHF BY PCR	SINDH	Karachi	NEGATIVE
2504	2022	CCHF BY PCR	KPK	Mardan	NEGATIVE
2505	2022	CCHF BY PCR	SINDH	Karachi	NEGATIVE
2506	2022	CCHF BY PCR	KPK	Peshawar	NEGATIVE
2507	2022	CCHF BY PCR	SINDH	Karachi	POSITIVE
2508	2022	CCHF BY PCR	KPK	Peshawar	NEGATIVE
2509	2022	CCHF BY PCR	SINDH	Karachi	NEGATIVE
2510	2022	CCHF BY PCR	SINDH	Karachi	NEGATIVE
2511	2022	CCHF BY PCR	SINDH	Karachi	NEGATIVE
2512	2022	CCHF BY PCR	PUNJAB	Lahore	NEGATIVE
2513	2022	CCHF BY PCR	Baluchistan	Quetta	NEGATIVE
2514	2022	CCHF BY PCR	SINDH	Karachi	NEGATIVE
2515	2022	CCHF BY PCR	SINDH	Karachi	NEGATIVE
2516	2022	CCHF BY PCR	PUNJAB	RAHIM YAR KHAN	NEGATIVE
2517	2022	CCHF BY PCR	SINDH	Hub	NEGATIVE
2518	2022	CCHF BY PCR	SINDH	Karachi	NEGATIVE
2519	2022	CCHF BY PCR	SINDH	Karachi	NEGATIVE
2520	2022	CCHF BY PCR	KPK	Peshawar	NEGATIVE
2521	2022	CCHF BY PCR	Baluchistan	Quetta	NEGATIVE
2522	2022	CCHF BY PCR	SINDH	Karachi	NEGATIVE
2523	2022	CCHF BY PCR	SINDH	Karachi	NEGATIVE
2524	2022	CCHF BY PCR	SINDH	Karachi	POSITIVE
2525	2022	CCHF BY PCR	SINDH	Karachi	NEGATIVE
2526	2022	CCHF BY PCR	KPK	Peshawar	NEGATIVE
2527	2022	CCHF BY PCR	Baluchistan	Quetta	NEGATIVE
2528	2022	CCHF BY PCR	KPK	Peshawar	NEGATIVE
2529	2022	CCHF BY PCR	SINDH	Karachi	NEGATIVE
2530	2023	CCHF BY PCR	Baluchistan	Quetta	NEGATIVE
2531	2023	CCHF BY PCR	Baluchistan	Quetta	POSITIVE
2532	2023	CCHF BY PCR	KPK	Peshawar	NEGATIVE
2533	2023	CCHF BY PCR	Baluchistan	Quetta	NEGATIVE
2534	2023	CCHF BY PCR	KPK	Peshawar	NEGATIVE
2535	2023	CCHF BY PCR	SINDH	Sukkur	NEGATIVE
2536	2023	CCHF BY PCR	Baluchistan	Quetta	NEGATIVE
2537	2023	CCHF BY PCR	SINDH	Karachi	NEGATIVE
2538	2023	CCHF BY PCR	SINDH	Karachi	NEGATIVE
2539	2023	CCHF BY PCR	SINDH	Karachi	NEGATIVE
2540	2023	CCHF BY PCR	Baluchistan	Quetta	NEGATIVE
2541	2023	CCHF BY PCR	SINDH	Karachi	NEGATIVE

2542	2023	CCHF BY PCR	Baluchistan	Quetta	NEGATIVE
2543	2023	CCHF BY PCR	SINDH	Karachi	POSITIVE
2544	2023	CCHF BY PCR	Baluchistan	Quetta	NEGATIVE
2545	2023	CCHF BY PCR	Baluchistan	Quetta	NEGATIVE
2546	2023	CCHF BY PCR	SINDH	Karachi	NEGATIVE
2547	2023	CCHF BY PCR	SINDH	Karachi	NEGATIVE
2548	2023	CCHF BY PCR	Baluchistan	Quetta	POSITIVE
2549	2023	CCHF BY PCR	SINDH	Karachi	NEGATIVE
2550	2023	CCHF BY PCR	KPK	Peshawar	POSITIVE
2551	2023	CCHF BY PCR	SINDH	Karachi	POSITIVE
2552	2023	CCHF BY PCR	SINDH	Karachi	NEGATIVE
2553	2023	CCHF BY PCR	Baluchistan	Quetta	NEGATIVE
2554	2023	CCHF BY PCR	KPK	Peshawar	NEGATIVE
2555	2023	CCHF BY PCR	Baluchistan	Quetta	NEGATIVE
2556	2023	CCHF BY PCR	KPK	Peshawar	NEGATIVE
2557	2023	CCHF BY PCR	KPK	Peshawar	NEGATIVE
2558	2023	CCHF BY PCR	SINDH	Karachi	NEGATIVE
