# Supplementary material for: Spatio-temporal distribution of Crimean-Congo Hemorrhagic Fever and its relationship with climate factors in Pakistan: A decade-long experience from tertiary care laboratory network
Source: PLoS One. 2025 May 12;20(5):e0320495. doi: 10.1371/journal.pone.0320495 (PMC12068632; doi:10.1371/journal.pone.0320495)
Supplement: S2 File — https://ds.data.jma.go.jp/tcc/tcc/products/climate/climatview/list.php?r=0&y=2025&m=1&s=1&e=0&k=0 (PDF) [file pone.0320495.s002.pdf]

Supplementary File 2

Monthly mean temperature and precipitation for the cities of Karachi, Quetta and Peshawar extracted from the online climate database of Tokyo Climate Center of the Japan Meteorologic Agency, part of the World Meteorologic Organization (WMO) Regional Climate Center in RA II.

https://ds.data.jma.go.jp/tcc/tcc/products/climate/climatview/list.php?r=0&y=2025&m=1&s=1&e=0&k=0

		KARACHI		QUETTA		PESHAWAR	
Year	Month	Mean Temperature	Precipitation	Mean Temperature	Precipitation	Mean Temperature	Precipitation
		[degC]	[mm]	[degC]	[mm]	[degC]	[mm]
2012	1	19.1	0	3	31	11	27
2012	2	20.6	0	4.1	35	14.1	50.1
2012	3	25.5	0	13	14	18.8	83.7
2012	4	29.2	0	17.6	108	23.9	52.1
2012	5	30.9	0	23.1	5	29.1	19.7
2012	6	30.7	0	26.4	0	32.5	26.1
2012	7	30.1	0	29.9	0	32.1	67.2
2012	8	29.3	7	28.2	0	31.3	92
2012	9	29.6	105	22.8	6	27.5	113
2012	10	28.8	0	16.1	0	22.6	13
2012	11	26	0	10.8	10	16.7	3
2012	12	21.4	23	6.1	32	12.6	77
2013	1	19.8	0	5.6	4	11.1	4
2013	2	22.2	20	6.8	103	13.7	181
2013	3	26.5	3	12.8	33	19.7	160
2013	4	28.7	30	16.7	63	23.5	83
2013	5	31	0	23.3	3	29.7	12
2013	6	32.3	0	29.6	1	32.4	21
2013	7	30.5	5	30.2		32.2	31
2013	8	29.2	105	26.9	15	30.3	75
2013	9	29.8	5	24.9	0	29.2	5
2013	10	30.1	1	18.8	0	24.6	18
2013	11	25.3	0	10.8	17	16	27
2013	12	21	0	5.5	0	12.4	1
2014	1	18.7	0	5.3	6	12.3	5
2014	2	21.5	0	4.9	32	13.6	40
2014	3	25.7	12	11.2	78	17	128
2014	4	29.2	0	18	26	23.2	53
2014	5	31.1	1	22	136	27.6	14
2014	6	32.4	0	28.3	2	33.5	30
2014	7	30.9	1	29.8	0	32.2	47

2014	8	30.1	10	27.9	0	30.9	62
2014	9	29.8	1	25		29.4	17
2014	10	29.6	0	18.9	3	23.2	43
2014	11	26.6	5	11.3	7	16.3	1
2014	12	21.3	0	6.3	0	11.1	0
2015	1	20.1		6.8	19	11.4	34
2015	2	23.3	2	10.3	34	15.3	69
2015	3	26	3	12.5	80	18.1	117
2015	4	30.5	0	19.5	38	24	121
2015	5	31.6	0	24.7	4	28.7	24
2015	6	33.6	0	29.3		32.4	2
2015	7	31	47	29.2	0	31.3	111
2015	8	29.4	1	28.4	1	30.1	155
2015	9	30.3	0	22.5	1	28.1	53
2015	10	30.2	0	18.7	4	23.4	54
2015	11	25.8	0	11.3	27	16.7	32
2015	12	20.9	0	6.1	3	11.7	8
2016	1	21.8	3	7.7	56	11.5	12
2016	2	23.2	0	8.9	0	15.3	26
2016	3	27.5	0	14	89	19.4	88
2016	4	29.2	0	19.1	4	24.2	61
2016	5	31.3	0	26	13	30.8	12
2016	6	31.7	65	28.6	5	33	40
2016	7	30.6	0	30.4	3	32.3	36
2016	8	29.6	99	27.3	0	30.9	42
2016	9	29.2	0	25.3	0	30.2	0
2016	10	28.8	0	17.7	0	24.7	
2016	11	25.4	0	10.8	0	17.3	0
2016	12	23.4	0	10.1	0	13	3
2017	1	19.2	40	5.2	67	11.4	54
2017	2	23.3		7.4	33	15.9	31
2017	3	26.7	0	13	55	19.7	22
2017	4	29.9	0	19.6	0	25.8	34
2017	5	31.7	0	24.8	1	30.8	2
2017	6	32.1	63	29.3	0	32.2	43
2017	7	30.2	33	29.4	0	31.8	92
2017	8	30.1	66	27.5	0	30.9	83
2017	9	29.6	26	23.4	0	28.8	10
2017	10	30.1	0	18.3	0	24.3	0
2017	11	25.2	0	10.9	5	16.4	61
2017	12	21	6	6	1	12.9	12

2018	1	21.3	0	6.6	2	11.5	0
2018	2	23.8	0	9.7	16	15.3	37
2018	3	27.9	0	16	49	20.8	26
2018	4	30.4	0	19.8	55		
2018	5	32.7	0	23.7	0	28.1	53
2018	6	31.7	0	29.2	0	32.8	18
2018	7	30.6	0	30	0	31.5	122
2018	8	29	0	28.8	0	31.5	24
2018	9	28.9	0	23.3		28.9	19
2018	10	30	0	16.3	0	21.8	29
2018	11	26.9	0	12	2	18.2	4
2018	12	21.2	0	7	0	10.8	18
2019	1	20.2	39	6.5	49	10.7	19
2019	2	21.6	0	6.2	67	12.8	57
2019	3						
2019	4	29.6	0			24.8	58
2019	5	31	0	22.5	9	27.8	36
2019	6	32.9	2	27.3	0	31.8	14
2019	7	31.4	66	30.3	5	32.9	37
2019	8	29.7	205	28.1	0	30.9	71
2019	9	31	52	25.8	0	30.5	1
2019	10	30.2	1	18.4	8	23.6	8
2019	11	25.5	0	9.6	39	17.1	31
2019	12	20.5	0	6.7	26	10.6	2
2020	1	18.4	0	2.8	101	10.3	47
2020	2	23.4	3	9	46	15	35
2020	3	25.6		12.1	22	17.3	151
2020	4	30.1	0	18	16	22.8	61
2020	5	31.9	0	23.1	17	28.5	8
2020	6	33.2	0	28.5	0	31.6	10
2020	7	32.6	101	29.4	1	33	25
2020	8	30.9	367	28	20	32.2	39
2020	9	31	0	22.4	0	28.5	56
2020	10	29.6	0	15	0	23.3	
2020	11	24	3	10.1	13	15.2	64
2020	12	20.6	0	5.8	6	12.2	9
2021	1	18.6	0	3.9	0	11.9	0
2021	2	23.7	0	11.4	1	16.4	23
2021	3	28.1	0	16	28	20.8	79
2021	4	30.9	0	20.5	4	24.3	20
2021	5	32.6	0	24	13	29.3	9

2021	6	32.2	0	28.8	0	33	0
2021	7	31	45	29.5	4	32.6	80
2021	8	29.5	0	28	0	31.3	41
2021	9	31.3	88	26.4	0	30.4	37
2021	10	28.6	17	17	1	24	7
2021	11	25.6	0	10.7	0	16.2	0
2021	12	21	17	7.2	1	11.3	
2022	1	19.2	29	5.7	74	10.9	111
2022	2	23.4	0	9.1	3	14.2	36
2022	3	28.1	0	17.4	6	22.9	5
2022	4	30.9	0	22.2	0	28.4	5
2022	5	31.7	0	25.4	0	30.6	20
2022	6	31.6	22	28.1	0	32.3	60
2022	7	29.9	348	27.2	63	30.9	68
2022	8	29.2	128	25	208	30.4	56
2022	9	29.7	43	24.3	0	29.9	5
2022	10	29.1	0	17.5	0	23.7	18
2022	11	25.9	3	12.3	4	16.9	51
2022	12	21.5	0	7.2	14	11.8	8
2023	1	18.5	0	2.8	51	11	11
2023	2	23.5	0	12.2	0	16.4	16
2023	3	27.1	6	14.1	80	19.9	61
2023	4	28.8	2	18.6	9	23.9	25
2023	5	31.1	15	23.8	13	27.4	27
2023	6	32.2	4	28.8	4	31.9	49
2023	7	31.4	40	29	7	31.3	90
2023	8	28.8	0	28.7	0	31.6	4
